# Genomic and Transcriptomic Analysis of the Polyploidy Cyst Nematode, *Heterodera trifolii*, and *Heterodera schachtii*

**DOI:** 10.3390/ijms26030948

**Published:** 2025-01-23

**Authors:** Parthiban Subramanian, Daegwan Kim, Hyoung-Rai Ko, Joon-Soo Sim, Vimalraj Mani, Chang-Muk Lee, Seon-Kyeong Lee, Soyoung Park, Dong-Gwan Kim, Yeisoo Yu, Bum-Soo Hahn

**Affiliations:** 1 National Agrobiodiversity Center, National Institute of Agricultural Sciences, Rural Development Administration, Jeonju 54874, Republic of Korea; parthi@korea.kr; 2 Department of Research and Development, DNACARE Co., Ltd., Seoul 06126, Republic of Korea; gardener@dnacare.co.kr (D.K.); yeisooyu@dnacare.co.kr (Y.Y.); 3 Crop Protection Division, National Institute of Agricultural Sciences, Rural Development Administration, Wanju 55365, Republic of Korea; reachsg@korea.kr; 4 Department of Agricultural Biotechnology, National Institute of Agricultural Sciences, Rural Development Administration, Jeonju 54874, Republic of Korea; jssim@korea.kr (J.-S.S.); vimal872@korea.kr (V.M.); changmuk@korea.kr (C.-M.L.); lsk220@korea.kr (S.-K.L.); psy0203@korea.kr (S.P.); 5 Department of Bioindustry and Bioresource Engineering, Sejong University, Seoul 05006, Republic of Korea; kimdg@sejong.ac.kr; 6 Plant Engineering Research Institute, Sejong University, Seoul 05006, Republic of Korea

**Keywords:** cyst nematode, *Heterodera*, genome assembly, transcriptome

## Abstract

Cyst nematodes remain a major threat to global agricultural production, causing huge losses. To understand the parasitism of the cyst nematodes *Heterodera trifolii* (HT) and *Heterodera schachtii* (HS), we constructed whole-genome assemblies using short- and long-read sequencing technologies. The nematode genomes were 379 Mb and 183 Mb in size, with the integrated gene models predicting 40,186 and 18,227 genes in HT and HS, respectively. We found more than half of the genes predicted in HT (64.7%) and HS (53.2%) were collinear to their nearest neighbor *H*. *glycines* (HG). Large-scale duplication patterns in HT and segmental duplications of more than half of the orthologous genes indicate that the genome of HT is polyploid in nature. Functional analysis of the genes indicated that 65.6% of the HG genes existed within the HT genome. Most abundant genes in HT and HS were involved in gene regulation, DNA integration, and chemotaxis. Differentially expressed genes showed upregulation of cuticle structural constituent genes during egg and female stages and cytoskeletal motor activity-related genes in juvenile stage 2 (J2). Horizontal gene transfer analyses identified four new vitamin biosynthesis genes, *pdxK*, *pdxH*, *pdxS*, and *fabG*, of bacterial origin, to be first reported in HT and HS. Mitogenomes of HT, HS, and HG showed similar structure, composition, and codon usage. However, rates of substitution of bases in the gene nad4l were significantly different between HT and HS. The described genomes, transcriptomes, and mitogenomes of plant-parasitic nematodes HT and HS are potential bio-resources used to identify several strategies of control of the nematode.

## 1. Introduction

Current agricultural production is stuck in a vicious loop between the need for large-scale monocropping for profitability and the high vulnerability of such single-crop systems to diseases and pests [1]. Among the major threats to agricultural productivity, plant-parasitic nematodes (PPNs) remain a formidable challenge extending across crop and geographical barriers, causing losses to the tune of billions of dollars globally [2,3]. The ability of nematodes to parasitize plants is independently emerged in the Nematoda [4]. With over 4000 represented species, these widespread nematodes can be considered one of the major factors that influence agricultural productivity on a global scale. The lifestyle of PPNs can be both ecto-parasitic where they live outside the host, are migratory, and feed on root cells (mostly from clade 1), and endo-parasitic where they move through the plant tissues or form permanent feeding structures, thus getting attached to the plant until the end of their lifecycle (clades 10b and 12) [4]. Among these, most damaging to agricultural productivity are the nematodes that have a sedentary endo-parasitic lifestyle, feeding on plant hosts, leading to loss of nutrients, thereby stunting, wilting, and abnormal development of roots, eventually leading to loss of yield. Most common of such PPNs are the root-knot nematodes (RKNs, *Meloidogyne* spp.), as well as cyst nematodes (CNs, *Globodera*, and *Heterodera* spp.), which command a high level of attention among researchers, as they have been the major PPNs worldwide for the past 20 years [5].

Compared to RKNs, cyst nematodes are more difficult to control, as the female worms typically form resilient structures called cysts, which protect the eggs as well as juveniles, making them hard to eradicate from the soil. CN members of genus *Heterodera* are significant as they are reported to infect large-scale monocrops such as potato, sugarbeet, soybean, and tobacco [6]. In South Korea, *Heterodera trifolii* (HT) and *Heterodera schachtii* (HS) have been reported to infect and cause agricultural losses in Chinese cabbage crops, which are a major crop of the country [7,8]. Crop rotation has been a conventionally used strategy against *Heterodera*, but its efficacy is limited by the ability of the cysts to remain dormant for a long period. Other conventional strategies for control of these PPNs have been incorporating native resistance through breeding, crop rotation, and treatment of seeds with nematicidal compounds with newer strategies such as raising genetically modified (GM) plants that express nematode-controlling biomolecules and use of microbial antagonists [9,10]. In recent times, acquired nematode ability to infect overused resistant crop varieties has also emphasized the need for their control [11]. Effective disease management is essential. Species of *Heterodera* are obligate biotrophs, which hinders their mass multiplication, leading to impractical sample sizes for laboratory-based studies. Combined, these factors have been huge barriers for sampling and have hindered progress in understanding parasitism of *Heterodera* at a genetic or molecular level until the last decade. For rigorous disease management, newer studies must aim at elucidating molecular clues from their genome to efficiently target these plant pests. Genome data can serve as a fundamental source of information to understand genes shared with other species of the genus, horizontally transferred genes, plant-infection-facilitating effectors, etc. [12,13,14]. Genomic information of a plant-parasitic nematode can provide several potentially useful information such as vital molecular factors, molecules, or proteins produced by the nematode for several important biological functions such as growth, pathogenesis, and reproduction, which can be used as targets for its control. Comparison of pathogenic and non-pathogenic nematode genomes can help us understand the molecular basis of pathogenicity. Moreover, genomic information can also be used to study the relationships between nematodes belonging to the same genus or family. Owing to the advancement in genome analyses techniques, several of these economically important plant-parasitic nematodes with genome sizes ranging from 50 to 100 Mb can be easily sequenced and studied upon [15]. Moreover, these nematodes exhibit different levels of ploidy [15]. Until now, among the *Heterodera* species, *H. glycines* has been well studied for its genomic characteristics and attributes [16,17]. Though hitherto published reports include partial data from the cyst nematode *H. schachtii* [1,18], there are not dedicated reports that describe in detail its genome structure and the genome of *H. trifolii* and remain to be studied yet. In the current study, genomes of cyst nematodes *Heterodera trifolii* and *Heterodera schachtii* are described in detail using short- (Illumina, San Diego, CA, USA) and long-read sequencing (PacBio (Menlo Park, CA, USA), Oxford Nanopore (Oxford, UK)), RNA-Seq, Iso-Seq, and mitochondrial genome sequencing to elucidate several aspects of their genome structure and function. Using this resource, we tried to identify genetic components, relationships with other plant-parasitic nematode genomes, transcription factors, effectors, and other secreted proteins of these two nematodes.

## 2. Results

### 2.1. Genome Sequencing

Use of multiple sequencing strategies can significantly improve the overall quality of the genome assembly. Here, preliminary sequencing data obtained from Illumina short reads, Oxford Nanopore, and PacBio Sequel are provided in Supplementary Tables S1 and S2. In brief, the highest number of reads was obtained using PacBio long-read sequencing, wherein HT generated 2.7 million reads with an average length of 11.1 kb, and in the case of HS, resulted in 2.8 million reads with a mean read length of 10.2 kb (Table S2). The N_50_ lengths were found to be highest in PacBio sequencing data, which for HT and HS were 18,750 bp and 17,250 bp, respectively. Assembly and polishing of HT and HS genomes from short- and long-read data indicated a genome size of 380 Mb constituted by 1409 scaffolds in the case of *H. trifolii*, followed by a 183 Mb genome constituted by 458 scaffolds in the case of *H. schachtii*. Genome completeness analyses using BUSCO indicated 58.3% and 57.8% of complete sequences for *H. trifolii* and *H. schachtii*, respectively (Table 1 and Table 2). The genome assemblies are available at the NCBI-Genome database under the accessions JBICBT000000000 (*Heterodera trifolii*) and JBICCN000000000 (*Heterodera schachtii*).

### 2.2. Genome Structure

#### 2.2.1. Gene Prediction, Synteny, Duplications, and Orthologous Genes

**Figure 1:**
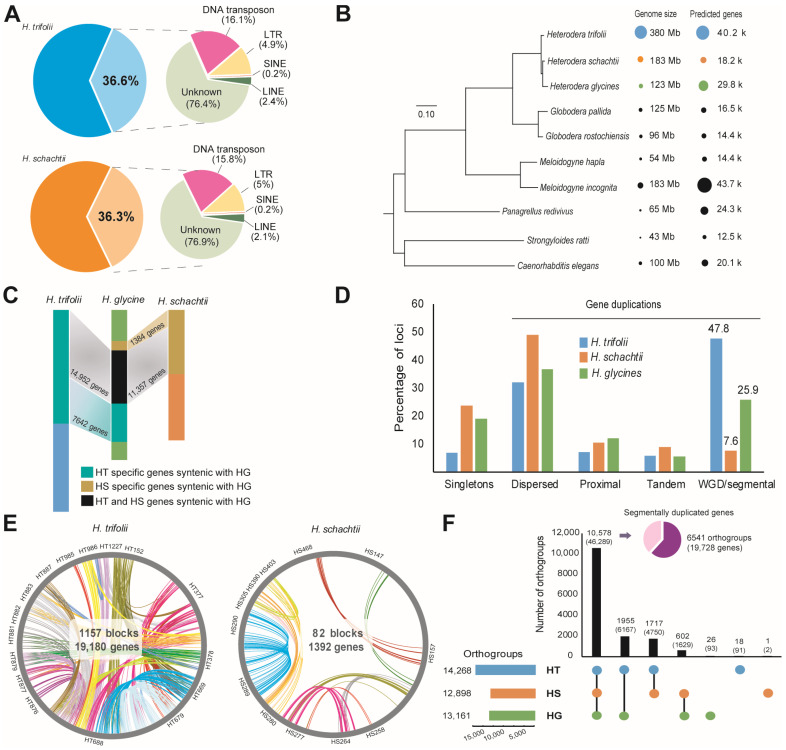
Genome structure of cyst nematode *Heterodera trifolii* and *H. schachtii* (**A**) Statistics of repeats observed in the assemblies of HT and HS genomes (**B**) A phylogenetic tree derived from horizontal gene transfer data indicating genome sizes of the studied nematodes and their nearest neighbors along with the number of genes predicted in each assembly. (**C**) Synteny of HT and HS genomes along with soybean cyst nematode *H. glycines* genome assembly. (**D**) Duplication patterns of genes observed in HT and HS. (**E**) Overview of duplications observed in the HT and HS genome assemblies. The labels are contigs identified in respective nematodes. (**F**) Orthogroups of genes identified in HT, HS, and HG genome assemblies. The inset chart shows the extent of whole-genome or segmental duplications (WGD/SD) observed within the orthologous genes.

Comparison of genome size and predicted genes indicated that HT possesses the largest genome among the currently reported *Heterodera* species and can even be considered the largest in the clade *Tylenchida*. Repeats made up the largest portion of the genomes of HT (36.6%) and HS (36.3%) (Figure 1A; Tables S3 and S4). Total repeats in the HT genome were 139 Mb in size, with unknown repeats being the highest (76.4%), followed by DNA transposons (16.2%), LTR (long terminal repeats) (4.9%), LINE (long interspersed nuclear elements) (2.4%), and SINE (short interspersed nuclear elements) (0.2%) (Table S3). In HS, the predicted repeats were 66 Mb, of which unknown repeats were the most common (77%), followed by DNA transposons (15.8%), LTR (5%), LINE (2.1%), and SINE (0.2%) (Figure 1A, Table S4). Motifs of microsatellites, or simple sequence repeats (SSRs), indicated that the high number of SSRs were 3–4 nucleotides long in both HT (62.3%) and HS (59.6%) (Table S5).

For gene prediction, results from integrated gene models for both nematodes were further filtered to remove any overlap with the repeat regions, and candidate genes with RNA-Seq read count values above zero or homology with the RefSeq vertebrate database and Uniprot database for *H. glycines* or *C. elegans* proteins were selected. The final gene model for HT consisted of 40,186 genes, and HS contained 18,227 genes (Figure 1B, Table S6). The exons per gene ratio in HS was 8.5 exons/gene compared to 7.7 exons/gene in HT (Table S6). Synteny analysis for studying the physically conserved order of genes comparing HT and HG indicated the presence of 1534 syntenic blocks (678 HT contigs vs. 430 HG contigs), which contained 22,594 genes in HT (64.7% of total predicted genes) (Table S7). The largest syntenic block identified contained 155 genes (Figure S1). In HS and HG, 699 syntenic blocks (188 HS contigs vs. 420 HG contigs) were identified that contained 12,741 genes in HS (53.2% of its predicted genes) (Table S7). The largest syntenic block identified contained 281 genes (Figure S1). A major portion of the collinear genes in HT (14,952 out of 22,594) and HS (11,357 out of 12,741) were syntenic in the HG genome, indicating close evolutionary relationships among the three nematode species (Figure 1C). Moreover, in both the studied nematode genomes, HG sequences were highly duplicated and dispersed among the HT and HS genomes. A dot plot alignment of the contigs identified between HG, HT, and HS confirmed the global duplication pattern, especially in the HT genome (Figure S2).

A detailed analysis of gene duplications in the nematodes indicated that in HG and HS, a majority of duplicate genes were dispersed, ranging at 36.9% and 48.9%, respectively (Figure 1D, Table S8). However, in the case of HT, we observed large-scale WGD/SD (47.8%) (Figure 1D, Table S8). In HT, 1157 blocks containing 19,180 (47.8% of total predicted genes) genes were found to be segmentally duplicated, and in the case of HS, this remained at 82 blocks containing 1392 genes (7.6% of total predicted genes) (Figure 1E). Orthologous gene search was performed by pair wise homology search for 244,350 protein sequences of a total of 10 nematode species (8 published nematode genomes from WormBase (WBPS15) and 2 nematode genomes from the current work). A total of 12,581 single-copy orthogroups were identified by Orthofinder analysis, and the phylogenetic tree based on the results indicated that HG possesses a close relationship with HS and also formed a distinct clade with HT (Figure 1B). Orthologous gene analysis to identify shared genes among the ten nematode species indicated that more than half of the predicted genes in HT (23,668 out of 40,186 genes; 58.9%) were orthologous in nature, whereas in the case of HS it was as high as 89% (16,286 of 18,227 genes), and in HG, the orthologous gene count was 19,067 genes, accounting for 64.2% of the predicted genes (Table S9). Further analysis of orthologous genes among members of the genus *Heterodera* including HG, HT, and HS, revealed that a majority of the predicted genes in HT and HS were shared with HG (10,578 orthogroups containing 46,289 genes) or among themselves (HT-HS, 1717 orthogroups, 4750 genes) (Figure 1F). This large-scale sharing of orthogroups (10,578) and the presence of only a few species-specific orthogroups in HT (18) and HS (1) indicate close evolutionary origin among the three *Heterodera* species. Furthermore, we found that the genes classified as segmentally duplicated to be abundant among the common orthogroups (HT-HS-HG) (6541/10,578 orthogroups), indicating that more than half of the segmentally duplicated genes were present in the last common ancestor of the genus *Heterodera* (Figure 1F).

#### 2.2.2. Transcriptome, Isoforms, and Differentially Expressed Genes

**Figure 2:**
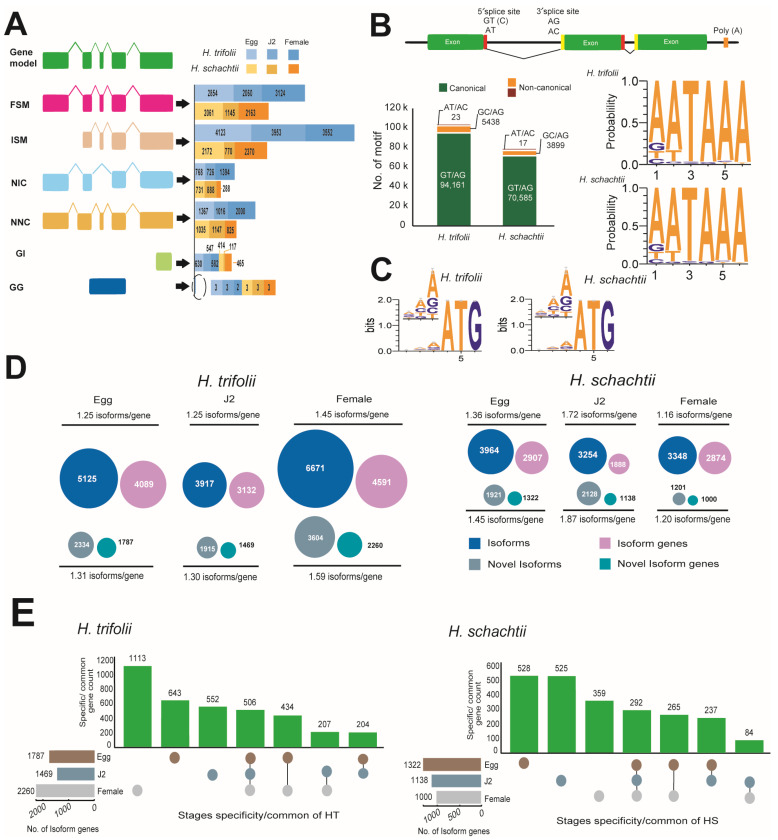
Transcriptome sequencing of HT and HS. (**A**) Classification of isoforms identified at various stages of growth in the nematodes HT and HS. Splice variants included full splice match (FSM), where all splice junctions perfectly matched with the reference transcript; incomplete splice match (ISM), with partial splice junction matches; novel in catalog (NIC), which are novel isoforms with a new combination of splice sites; novel not in catalog (NNC), which were also novel isoforms and contained at least one new splice site; genic intron (GI), which were within an intron; and genic genomic (GG), which overlapped introns and exons. (**B**) Types and statistics of different splice sites identified in HT and HS (**C**) Nucleotide bias in positions that precede the start codon in Kozak sequences identified in the transcripts of HT and HS. The bit at each position means the information content and the heights of individual bases are in proportion to their frequencies. (**D**) Statistics of isoforms and novel isoforms identified at various stages of the life cycle of the nematodes HT and HS. (**E**) Isoform gene statistics and stage-specificity observed in nematodes HT and HS.

Transcriptomes of the cyst nematodes HT and HS were sequenced bp or less. The overall HT assembly was 32.9 Gb in size with 246,325 isoforms, and in the case of HS, the assembly remained at 14.3 Gb with 83,761 predicted isoforms (Table S10). RNA-Seq sequencing in both HT and HS resulted in 16.8 Gb and 18.4 Gb of reads, respectively (Table S11). Upon mapping with RNA-Seq reads, the predicted isoforms were initially classified based on their splice sites as full splice match (FSM), where all splice junctions perfectly matched with the reference transcript; incomplete splice match (ISM), with partial splice junction matches; novel in catalog (NIC), which are novel isoforms with a new combination of splice sites; novel not in catalog (NNC), which were also novel isoforms and contained at least one new splice site; genic intron (GI), which were within an intron; and genic genomic (GG), which overlapped introns and exons (Figure 2A). Also, during the mapping of isoforms with the RNA-Seq data, we observed that the majority of splice sites to be canonical (GT/AG) in nature in both HT (94.5%) and HS (94.7%). Other non-canonical splice sites, in order of their abundance, were GC/AG: HT (5.46%); HS (5.23%) and AT/AC: HT (0.02%); HS (0.02%) in both HT and HS (Figure 2B).

**Figure 3:**
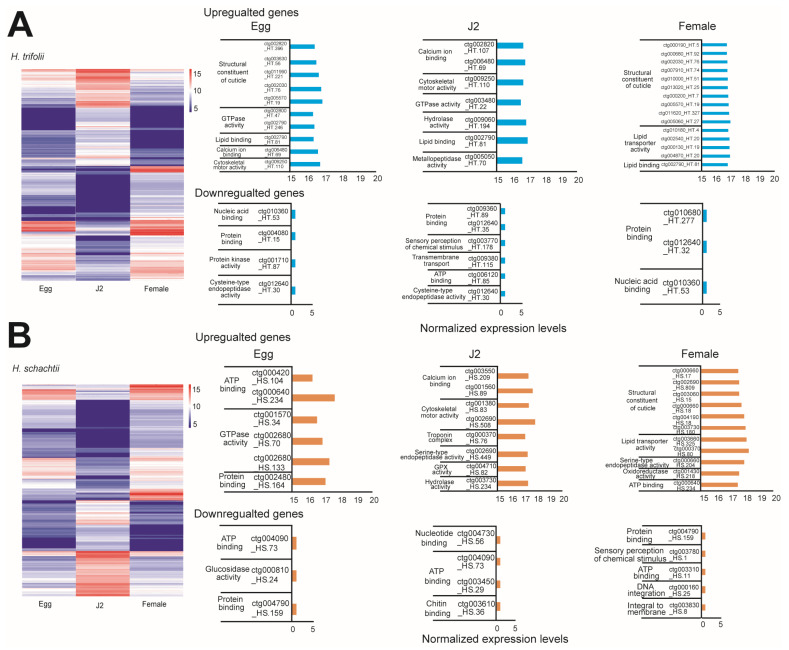
Differential gene expression at various stages of the life cycle of HT and HS. (**A**) A heatmap displaying differentially expressed genes based on different life stages of HT, along with highly upregulated as well as downregulated genes at specific stages of the HT life cycle. (**B**) Heatmap displaying differentially expressed genes based on different life stages of HS along with highly upregulated as well as downregulated genes at specific stages of the HS life cycle.

Comparison of poly A-motifs among the egg, J2, and female stages, as well as in both nematodes, indicated that the most common motif was 5′-AATAAA-3′ and the least common motif was 5′-AACAAG-3′. Other common motifs observed were consistently the same in both HT and HS and were 5′-ATTAAA-3′ and 5′-GATAAA-3′ (Figure 2B). Analysis of the Kozak sequence identified that the variability in the bases in positions preceding the start codon were similar in both HT and HS (Figure 2C). In both nematodes, the most and least preferred bases in the −1 position were A/T, in the −2 position were A/G, and in the −3 position, A/T. Overall, adenine was most preferred as the base that precedes the start codon. Stage-wise comparison of spliced and novel isoforms, as well as gene counts, indicated HT possessed a high number of spliced and novel isoforms compared to HS (Table S12). Though HS possessed a fewer number of isoforms compared to HT, isoform lengths were longer. More commonly, in both nematodes, novel isoforms were diverse, which can be seen by the high ratios of isoforms/gene (Figure 2D). Stage-specific isoform gene counts indicated that in HT, stage-specific genes were high during the female stage, followed by the egg and J2 stages. Whereas, in the case of HS, the highest number of isoform genes was observed in the egg stage, and the female stage had the least number of specific isoform genes (Figure 2E). Analysis of differentially expressed genes was carried out for 32,958 genes in HT and 16,804 genes in HS (Figure 3). DESeq2 normalized (vsd) expression values indicated that in HT, a high number of genes were upregulated (vsd > 15) during the female (212 genes) and J2 stages (196 genes) compared to the egg stage (143 genes) (Figure 3A). A similar trend was observed in HS, where a high number of upregulated genes (vsd > 15) during J2 stages (236 genes) in females (310 genes) was compared to the egg stage (135 genes) (Figure 3B). As the juvenile and female stages are active stages of the nematode, where the worm is metabolically active, either seeking a host for survival or involved in producing eggs, upregulation of several can be anticipated in both these stages. Comparison of DEG using log_2_ fold change values between stages indicated distinct differences in expression levels in both the nematodes (Figure S3). A large number of genes were up- and downregulated when comparing the egg vs. J2 stages as well as the J2 vs. female stages in both HT and HS, where the increase or decrease in expression levels was between 1- and 3-fold. When egg vs. female expression levels were compared, a large number of genes were upregulated in female stages. Correlation analysis of DEG across various life stages of HS and HT indicated that a majority of the genes were shared between egg and female stages on both the nematodes (Figure S4). The functional aspects of the up and downregulated (<10 × log_2_ fold change) genes that had hits in the Uniprot and GO databases are provided in Figure 3.

### 2.3. Genome: Functional Annotation of Genes

**Figure 4:**
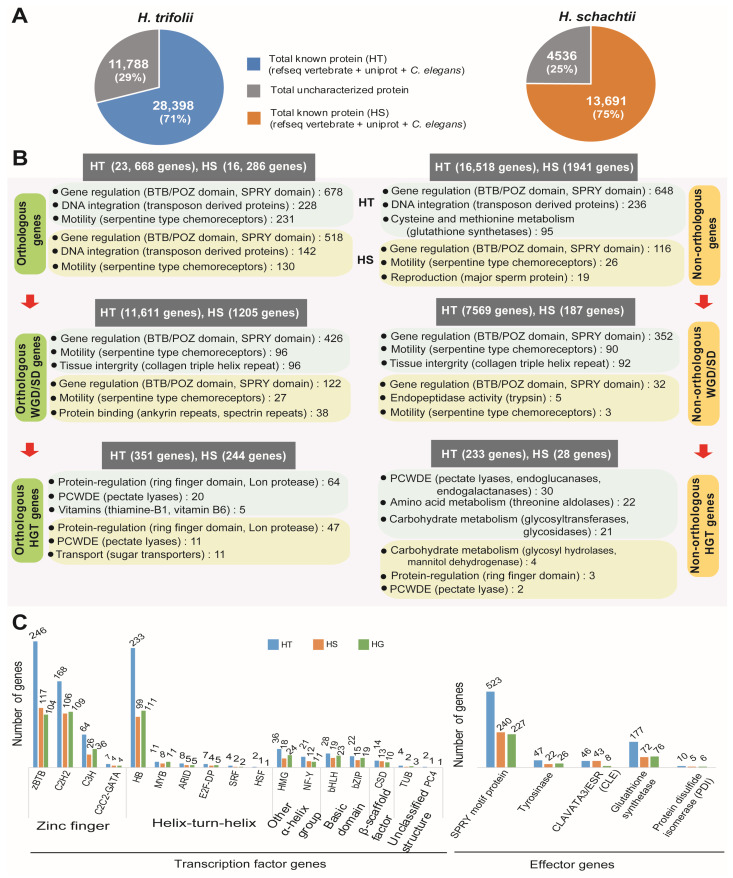
Functional analysis of genes identified in the genome assemblies of HT and HS. (**A**) Statistics of genes with functions matched in the analyzed databases for HT and HS. (**B**) Overview of major metabolism/functions in the identified genes based on the number of hits in the databases. Numbers given in parentheses indicate the number of genes with similar function identified during the database search. (**C**) Identification of transcription factor genes and major families of effector genes in HT, HS, and HG genome assemblies.

The BLASTp program was used to search for gene functions for the integrated gene models of two nematodes. For gene function search, NCBI vertebrate RefSeq database (10,677,468 sequences), Uniprot DB (557,491 sequences), *C. elegans*, and *H. glycines* annotation from Wormbase were used as references. Search of the conserved protein domain, gene ontology, and metabolic pathways was performed on the Pfam, GO, and KEGG databases using the InterProScan program (v5.38-76.0). Overall gene function of 28,398 genes (70.7% of 40,186 genes) (Figure 4A) were identified in HT. In the case of HS 13,691 genes, there was a hit in at least one of the referred databases, which accounted for a 75.11% match of a total of 18,227 genes (Table S13). Identifying the functional aspects of syntenic genes in HT and HS using gene ontology enrichment indicated conservation of thiamine biosynthesis and metabolism (Table S14). Syntenic genes in HT had diverse functions, including several basic metabolic processes and structural processes, whereas in the case of HS, identified syntenic genes were mainly involved in cellular respiratory processes (Table S14).

Functional annotation of predicted genes in HT and HS are given in detail in Supplementary Materials (Data S1 and Data S2, respectively). The most common gene functions identified in both nematodes were protein binding, DNA integration, and motility. In HGT genes, we observed several plant cell wall degrading enzymes (PCWDEs), carbohydrate metabolism-related enzymes, and vitamin biosynthesis-related genes (Figure 4). In HT, overrepresented GO terms included protein, ATP, and DNA-binding proteins, which include the presence of BTB/POZ, SPRY domains, 14-3-3 proteins, and several transposon proteins. Whereas in the KEGG database, glutathione metabolism, cysteine, and methionine metabolism were highly represented in the predicted genes, which included several copies of glutathione synthases (Figure 4B, Table S15). In HS, we found a similar pattern of gene function, but among the HS-specific genes (non-orthologous genes), we found enrichment of genes related to motility, which included serpentine-type chemoreceptors and genes related to reproduction (major sperm protein) (Figure 4, Table S16). A list of orthologous genes detected in HT and HS is provided in Supplementary Data S3. Species-specific orthogroups detected in HS contained 2 genes (1 orthogroup) coding for BTB/POZ domain-containing protein 6-A. In HT, 91 genes were detected (18 orthogroups) that coded for a PDZ domain-containing protein, C52A11.3, and a receptor-type guanylate cyclase, gcy-13, in addition to uncharacterized proteins (Table S17). The whole genome or segmental duplications (WGD/SD) followed a similar enrichment of protein and nucleic acid-binding proteins, which were majorly BTB/POZ domain-containing proteins involved in gene regulation and motility-related serpentine-type chemoreceptors (Figure 4, Table S18). In HT, we also observed proteolysis (70 genes) and the ubiquitin-dependent protein catabolic process (37 genes), which might have a regulative function (Table S18). GO enrichment analysis indicated that, in the segmental duplicated loci of both the cyst nematodes, protein, ATP, and DNA binding were the major molecular functions (MF) identified (Supplementary Data S4). While transmembrane transport, proteolysis, and amino acid glycosylation were the major biological processes (BP) detected in HT, transmembrane transport, protein secretion, and signal transduction were the major BPs detected in HS (Table S18). In HT, the largest segmental duplication contained 107 genes and occurred between blocks HT189 (722 kb, 184 genes) and HT204 (504 kb, 120 genes). The GO enrichment of these genes was predicted to have ATP, calmodulin, heme, nucleic acid, and protein-binding activities; acetylgalactosaminyltransferase, cytochrome-c oxidase, ubiquinol-cytochrome-c reductase, oxidoreductase, respiratory electron chain, and extracellular ligand-gated ion channel activity localized to the membrane and mitochondrial inner membrane. In HS, the largest segmental duplication block contained 40 genes and occurred between tandem blocks HS289 (800 kb, 69 genes) and HS290 (849 kb, 72 genes). GO enrichment of these 40 genes indicated functions such as DNA and protein binding activities; glutaminase, hydroxyethylthiazole kinase, and metalloendopeptidase activities; structural constituent of cuticle; and cytoskeletal anchoring at the plasma membrane. Identification and comparison of transcription factor (TF) genes indicated the presence of a high number of zinc-finger TF genes, followed by helix-turn-helix TF genes, in both the nematodes (Figure 4C, Table S19). Effector gene families have been widely reported in parasitic nematodes, and in the current study, we identified commonly reported effector genes in the genomes of HT and HS. We identified a total of 803 effector genes in HT, which was more than double the number of effector genes identified in HS (382 genes) and HG (343 genes). Among the reported effector families, SPRY motif proteins were the most common in both HT (65.1%) and HS (62.8%), whereas protein disulfide isomerases were found to be the least common (HT—1.2%; HS—1.7%) (Figure 4). Other detected effector genes included tyrosinases, Clavata3, and glutathione synthases.

In the case of the isoform genes, in HT, the most common gene ontology terms observed were protein binding (398 genes), ATP binding (277 genes), followed by nucleic acid binding (200 genes). The most common KEGG pathways were the mTOR signaling pathway and the PI3K-Akt signaling pathway. The common Pfam domains identified were the nematode cuticle collagen N-terminal domain and the protein kinase domain (Supplementary Data S5, Table S20). This was similar also in HS, but the most common Pfam domains identified were the protein kinase domain and RNA recognition motif. (a.k.a. RRM, RBD, or RNP domain) (Table S21). Further, differential expression of genes (DEG) analyses indicated changes in expression patterns of several genes across the life stages of the nematodes. In HT the highest number of changes in expression was observed when comparing juvenile and female stages of the nematode compared to juvenile vs. female and egg vs. female (Supplementary Data S6). In HS, the highest number of changes in expression was observed when comparing egg and juvenile stages (1615 genes) of the nematode compared to juvenile vs. female and egg vs. female. Analysis of genes that were highly up- and downregulated (>10 × fold log_2_ change) indicated that HT-upregulated genes in the egg stage were involved in cuticle collagen synthesis, actin genes, and elongation factor 1-alpha, whereas highly downregulated genes were involved in nucleic acid and protein binding activities (Figure 3, Table S22). In the J2 stage of HT, we observed the production of plant cell wall degrading enzymes (PCWDE) and chemotaxis-related proteins to be upregulated and transport as well as protein-binding genes to be downregulated. In female stages we found several genes related to the structural constituents of the cuticle to be upregulated, whereas protein and nucleic acid-binding proteins were highly downregulated (Figure 3). This trend was also similar in HS, except for its egg stage, where we did not detect any upregulation of genes related to structural constituents of the cuticle (Figure 3, Table S23).

**Figure 5:**
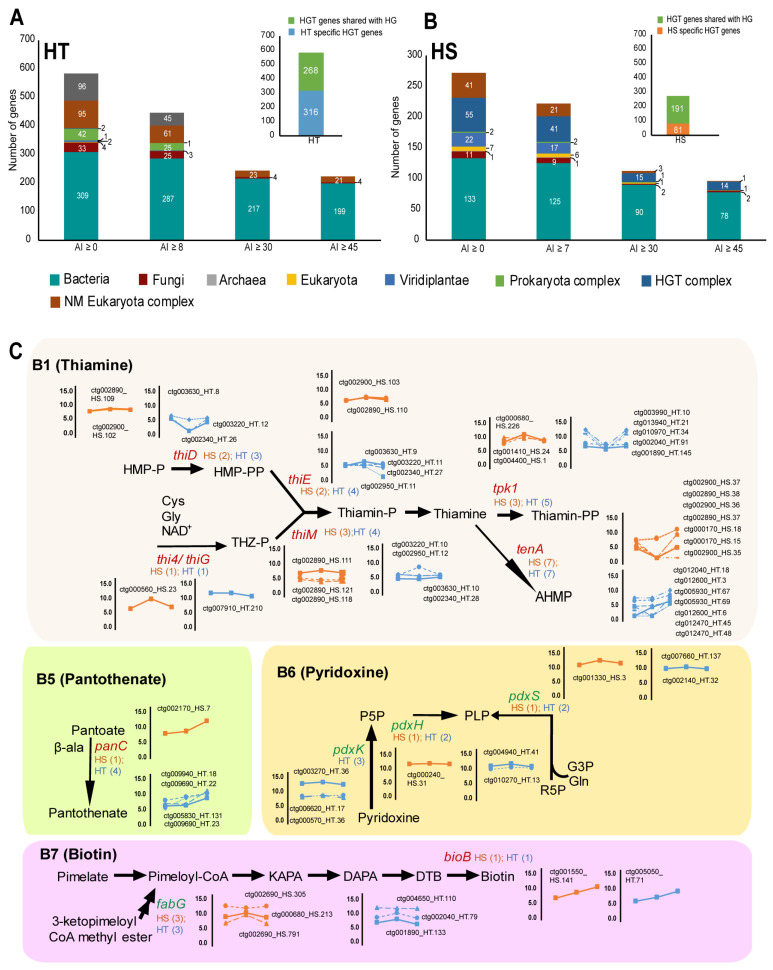
Evidence of horizontal gene transfer (HGT) observed in genome assemblies of HT and HS. (**A**,**B**) Horizontally transferred genes were identified in HT and HS using the AvP program by a survey in various databases. AI—alien values and numbers indicate the number of gene hits in respective databases. (**C**) Identification of vitamin biosynthesis genes among the HGT genes involved in the synthesis of vitamins B1, B5, B6, and B7 in HT and HS. The genes in red font indicate genes identified in HT and HS that have been already reported in soybean cyst nematode HG, and genes mentioned in green (*pdxK*, *pdxH*, *pdxS*, and *fabG*) are first reported in *Heterodera* in this study. Line graphs adjacent to the pathway show the expression levels of the genes at various stages of the nematode life cycle.

Environmentally acquired genes, or horizontal gene transfer (HGT), have been extensively reported in parasitic nematodes. On comparison of the protein sequences of the nematodes in the Uniprot database for candidate horizontally transferred genes, 584 putative HGT genes were observed in HT and 272 putative HGT genes were observed in HS with alien index values above zero (AI > 0) (Supplementary Data S7, Figure 5). Calculating F1 scores to balance precision and sensitivity of detection, the putative HGT gene candidates were reduced to 447 in HT (AI ≥ 8, F1 score 8) and 222 genes in HS (AI ≥ 7, F1 score 7) (Supplementary Data S7). In both HT and HS, most of the candidate HGT genes were found to be acquired from bacteria and overlapped with HGT genes of HG (Figure 5A,B). Among the HGT genes, we found several plant cell wall degrading enzyme-coding genes, protein regulation genes, carbohydrate metabolism genes, and genes involved in vitamin synthesis (Figure 5, Supplementary Data S7). Several HGT genes involved in vitamin synthesis have been reported in nematodes. We identified several genes in HT and HS involved in the biosynthesis of vitamins B1, B5, B6, and B7, which have been previously reported in HG. Additionally, we report for the first time several copies of *pdxK*, *pdxH*, and *pdxS* involved in vitamin B6 (pyridoxine) biosynthesis and the *fabG* gene involved in vitamin B7 (biotin) biosynthesis in the genomes of nematodes HS and HT (Figure 5C).

### 2.4. Mitochondrial Genome

**Figure 6:**
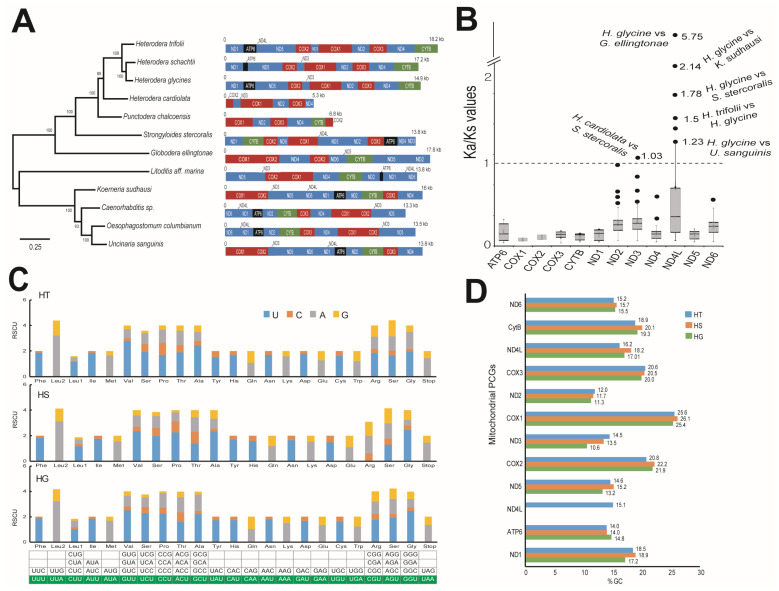
Mitochondrial genomes of nematodes HT and HS. (**A**) A phylogenetic tree derived from comparing the mitochondrial genome sequences of the nematodes. On the right are various genes identified and indicative sizes of the mitochondrial sequences. (**B**) Molecular evolution and selection in mitochondrial genes interpreted through the ratio of nonsynonymous substitutions (Ka) to synonymous substitutions (Ks). The genes, values observed, and compared nematodes are given in the graph. A threshold value of 1 was taken to identify positive selection. (**C**) Relative synonymous codon usage (RSCU) statistics among the nematodes HT, HS, and HG. The codons with RSCU values of >1 are highlighted in the table below and were found to be consistent among the three nematodes. (**D**) Comparison of the GC % in various protein-coding genes (PCGs) identified in the mitochondrial sequence of HT, HS, and HG. The PCGs ND—NADH dehydrogenase; CytB—cytochrome B oxidase; COX—cytochrome C oxidase; ATP—ATP synthase.

The assembled mtDNA was 18,235 bp long in the case of HT and 17,175 bp long in HS (Figure S5). NCBI-BLAST analysis indicated the presence of protein-coding genes (PCGs), transfer RNA, and ribosomal RNA sequences in both HT and HS (Tables S24 and S25). The identified PCGs in HT and HS have been consistently reported in nematode mitochondria and included several subunits of NADH dehydrogenases, ATP synthases, and cytochrome oxidases, which are related to respiratory metabolism (Figure S5, Tables S24 and S25). The order of the PCGs in the mitogenome was similar in HG, HT, and HS but was distinct from other neighboring mitogenomes. Phylogenetic analysis using the mtDNA indicated a close relationship between HS and HG, where HT formed a distinct but neighboring clade (Figure 6). Evolutionary analyses using the Ka/Ks ratio indicated that among the 12 PCGs, *nd4l* and *nd3* had a higher non-synonymous substitution rate compared to their counterpart species (Ka/Ks > 1) (Figure 6B, Table S26). We observed that HG had a higher Ka/Ks ratio in *nd4l* against several other nematodes such as *Globodera ellingtonae*, *Koerneria sudhausi*, *Strongyloides stercoralis*, and *Uncinaria sanguinis*. Surprisingly, the HG *nd4l* gene was significantly higher than the *nd4l* gene of HT (Figure 6B). In the case of *nd3*, HG showed a higher Ka/Ks ratio against *S. stercoralis.* Codon usage analysis using the relative synonymous codon usage (RSCU) values indicated that the codon usage was highly conserved among the three nematodes, HG, HT, and HS, where the optimal third base of the codon used was consistently similar and significant (>1) for all the amino acids in all three species (Figure 6C). Comparison of GC content in the PCGs was also found to be consistent and had a significant A-T bias (>70%), which is common in most reported nematode mitogenomes (Figure 6D).

## 3. Discussion

The development of parasitism to plants among nematodes is of great interest because plant-parasitic nematodes are found to be distributed in four distinct clades among the twelve clades of nematodes, suggesting the occurrence of independent evolutionary events on at least four separate occasions [4,19]. Genomes of PPNs from order *Tylenchida*, which include genera *Globodera*, *Heterodera*, *Meloidogyne*, *Pratylenchus*, and *Radopholus*, have been extensively sequenced [4,19]. Information from these whole genomes can give us a glimpse of the life of these PPNs, including their life cycle, conserved metabolic pathways, parasitism towards hosts, and evolution. The cyst nematode *Heterodera* is one of the most important PPN, causing substantial economic losses and proving difficult to eradicate from the soil [20]. Genomic studies on the members of the genus have been initiated since the last decade, and the whole genomes of hitherto published cyst nematodes from the genus *Heterodera* have been from the soybean cyst nematode *H. glycines* and sugarbeet nematode *H. schachtii* [1,16,17,18]. In the current study, we have generated genome models of *H. trifolii* and *H. schachtii* using a combination of genomic as well as life stage-specific transcriptome sequencing data.

Earlier reports have suggested the genome of HS to be at a range of 179–190 Mb with 395–705 predicted scaffolds (Table 2). The more recent data from [1,18] have given a final assembly statistic of 179 Mb, 395 scaffolds with an N_50_ length of 0.2 Mb at 192-fold coverage. Currently, this assembly is the largest among reported HS genomes, also with longer scaffolds and higher genome completeness (Table 2). On the other hand, this is a primary report on the genome of the cyst nematode *H. trifolii.* The assembled HT genome length was 1.9 times larger than HS; completeness was found to be 58.3%, and this assembly of *H. trifolii* is now the largest genome among the *Heterodera* as well as the plant-parasitic cyst nematodes reported so far. Genomes of phylogenetically associated plant-parasitic nematodes such as *Globodera* and *Meloidogyne* were found to be much smaller in size, ranging at 54–183 Mb (Figure 1).

Considering the genus *Heterodera*, repeat sequences in both HS (66.4 Mb) and HT (139.1 Mb) were much higher compared to a recent robust genome assembly of HG (61.4 Mb), which was the highest reported earlier [17]. Repeats in other PPNs were estimated at 17 Mb for *G. pallida* [21] and 4 Mb for *Hoplolaimus galeatus* [22]. During gene prediction, we observed an exon-to-gene ratio of 7.7 (HT) and 8.5 (HS), which is much less compared to 11.2 in *Meloidogyne graminicola* (11.2), *G. rostochiensis* (8.7), and *G. pallida* (8.04) [14,17]. A low number of exons per gene indicates a simpler structure of the genome with fewer interruptions by introns, improving efficiency.

Gene duplications can help us understand the evolutionary origins of genes and are understood to be a common way for newer genes to originate [18,23]. The segmental duplications in HG and HS are in a similar trend as described earlier and were orthologous in nature [17]. GO enrichment of these segmentally duplicated genes indicated that a majority were localized to the membrane and were found to be involved in transmembrane transport and protein binding (Table S18). Synteny analyses confirmed duplication of the HG genome in HT, indicating that the duplicated genes may be shared from a common ancestor (Figure S2). Synteny and collinear gene analysis among the *Heterodera* species indicated that HT and HS contained more than 500 syntenic blocks with HG; more than half of the predicted genes in HT (64.7%) and 53.2% of the genes in HS were collinear to the genes of HG. Extended sharing and duplication of genes among the three *Heretodera* species was further confirmed by studying the gene orthologues, which identified 10,578 orthogroups containing 46,289 genes common to HG, HT, and HS. This is much higher than orthologous genes detected in PPNs of *Globodera* and *Meloidogyne* [21]. Ortholog-based phylogenetic analysis indicated HG to have a close phylogenetic relationship with HS, whereas HT formed a separate but close clade. Therefore, it could be understood that the genomes of HS and HT have a majority of their elements shared with the *H. glycine* genome, and there is proof of a high degree of conservation of genes among the members of *Heterodera.*

On studying the gene function, generally, the number of protein-coding genes in plant-parasitic nematodes is reported to occur at a range of 14 k to 19.2 k [19]. The HT genome proposed here consisted of 40.2 k genes, and HS was predicted to contain 18.2 k genes. Elucidating gene function in the highly conserved syntenic genes indicated that thiamine metabolism-related genes were found in high numbers in both HS and HT. Thiamine, or vitamin B1, through its derivative thiamine pyrophosphate (TPP), acts as a cofactor for enzymes in oxidative pathways after glycolysis and plays different roles in glycolysis, the Krebs cycle, and the pentose phosphate pathway [24]. Also, 18 HS and 43 HT annexin-coding genes were identified. This protein is localized to the eggshell and helps hatching by modifying the permeability of the eggshell in response to the presence of host root exudates [25]. We also observed several putative arabinanases, invertases, and ubiquitins among the annotated genes, which have been reported to be produced in the dorsal gland of cyst nematodes [4,19].

Effectors play a crucial role in pathogenicity, and we detected the presence of several effector genes in both HS and HT. Among the detected effectors were several glutathione peroxidases and peroxiredoxins that have been reported to metabolize ROS produced by plants in response to infection in *G. rostochinensis* [19]. Horizontal gene transfer in nematodes compared to prokaryotes is rare (around 1% of genes in *Meloidogyne* originated from HGT). Obtained from microbes, they support the parasitic lifestyle of nematodes [26], and the occurrence of PPNs in different nematode clades indicates the independent evolution of parasitism to plants, which can be only explained through HGT, and several PPNs are closely related to fungivorous nematodes [4]. Earlier reports on HGT genes in HS reported 263 genes [18], and other reports include HG (82 genes, AI ≥ 0), *M. graminicola* (67 genes, AI ≥ 14), and *G. rostochiensis* (519 genes, AI ≥ 0). From the current report, among the members of the genus *Heterodera*, HT can be seen to have the highest number of reported HGT genes. We found the HGT genes of HT and HS to be significantly shared with HG (Figure 5). Several of these common putative HGT genes were involved in transport (major myo-inositol transporter) and oxidoreductase activity (mannitol 2-dehydrogenase), whereas putative HGT genes specific to HT were rich in plant cell wall degrading pectate lyases and *N*-glycosidases, whereas in the case of HS, probable arabinogalactan endo-beta-1,4 galactanases and pectate lyases were observed (Supplementary Data S7). Several HGT vitamin pathway genes involved in the synthesis of vitamins B1 (thiamine), B5 (pantothenate), B6 (pyridoxine), and B7 (biotin) have been reported in the past from HG [27,28]. Recently, the importance of the putative HGT vitamin B5 pathway gene in the pathogenicity of nematode HS was elaborated in detail [18]. In our study we identified several genes for vitamin biosynthesis, namely *pdxK*, *pdxH*, and *pdxS* (vitamin B6) and *fabG* (vitamin B7), which have been earlier reported in microorganisms but not PPNs. Studies on the transcriptome of the nematodes indicate that, earlier, the genus *Globodera* was reported to have the highest frequency of non-canonical GC/AG introns among nematodes with *G. rostochiensis* (3.46%) and *G. pallida* (3.53%) [29]. The percentage of non-canonical GC/AG introns among other PPNs were reported to be 0.45% in *M. graminicola* and 2.36% in *Rotylenchulus reniformis* [14,29]. However, we observed both the nematodes to have much higher frequency, 5.4% (HT) and 5.2% (HS) of GC/AG introns, which can improve the proteome diversity in these nematodes and also provide potential targets for control [30].

Nematode mitogenomes are characterized by recurrent rearrangement of their genes, unique initiation codons, and frequent rates of nucleotide substitution, which make them ideal candidates to study their evolution [30]. Most of the nematode mitochondria are characterized by 12 protein-coding genes (PCGs), and we observed that the order of arrangement of the PCGs were consistent in the nematodes HT, HS, and HG (Figure 6A). Studying the evolution of these mitogenome genes through their Ka/Ks ratio indicated high substitutions occurring in HG in the NADH dehydrogenase subunit 4L (*nad4l*). There was a significant (>1) mutation rate in the *nd4l* gene even between HG and HT (Figure 6B). This indicated a positive selection of the gene in HG when compared with HT. Overall, an A+T bias was observed in the studied nematodes HT and HS (Figure 6D). This is common in nematode mitochondria, where G+C content of genes is often found to be below 30% [31]. In the current study, all PCGs, including those of HG, showed G+C content at a range of 10.6 to 26.1% (Figure 6D). Studying the relative synonymous codon usage (RSCU) showed that the *Heterodera* species in this study were consistent in their codon usage for all the amino acids (Figure 6C).

We now understand that the genome of HT is by far the largest among the *Heterodera* species, with high duplication patterns of genes that are collinear with HG but phylogenetically distant from HG among the three nematodes. The genome of HT harbored almost 1.5 times more genes compared to HS, but the gene density in HT was much lower (106.4 genes/Mb) than HS (131.6 genes/Mb) compared to closely related *Globodera* species (132.2–149.9 genes/Mb) [19]. Overall, considering global duplication of HG genes in the HT genome and large-scale whole-genome duplications (WGD) pattern, we suspect the genome of HT to be polyploidy in nature. Polyploidy has also been observed in *Meloidogyne* (*M. incognita* 3 n = 40–48; *M. arenaria* 3 n = 50–56) [19]. Generally, parthenogenic nematodes are susceptible to polyploidization, and species with smaller effective population sizes have larger genomes as they tend to accumulate repetitive DNA and genome duplications [26,32]. Among the studied nematodes, the HT genome fits into this and can be suspected to be polyploidy in nature.

Cyst nematodes are a major class of nematodes crossing geographical barriers and causing huge losses in agriculture each year, especially in monocultivated fields. In a country such as South Korea, the cyst nematodes HT and HS have caused major agricultural damage in Chinese cabbage crops, which are predominantly used to make kimchi, a famous dish in the Korean cuisine. In the current study we sequenced the genome, mitochondrial genome, and transcriptome of cyst nematodes HT and HS and assembled genomes using a combined approach to improve the quality of the final assembly. The assembled genome was found to have close synteny with soybean cyst nematode *H. glycines*, share collinearity indicating the close physical relatedness of the genomes and identify several effector genes, orthologous genes, and horizontally transferred genes, which contributed to the parasitism of the nematodes. Increased understanding of the interactions that occur between plants and nematodes can help us find more ways to control infection of plants by nematodes [33]. These include several approaches, such as understanding the physiology of nematodes, their genetic components, such as effectors, and regulatory molecules for the development of effective control strategies.

## 4. Materials and Methods

### 4.1. Nematode DNA Extraction

The CNs HT and HS were propagated in Chinese cabbage plants grown in a sterilized sand + clay mixture (80% + 20% by volume) maintained at 25 °C and 70% relative humidity and harvested at egg, juvenile, and female stages. DNA samples for short- and long-read sequencing were isolated using the CTAB method and high-salt phenol extraction methods, respectively [34]. Total RNA isolation was carried out at three respective stages (egg, juveniles, and female) using TRIzol reagent (ThermoFisher, Waltham, MA, USA). Preparation of mitochondrial DNA was carried out using a rapid miniprep protocol [35].

### 4.2. Genome Sequencing and Assembly

Illumina short read, Oxford Nanopore, and PacBio sequencing were performed at DNACARE Co., Ltd., Seoul, Republic of Korea. Paired-end short-read sequences were produced using the Illumina NovaSeq 6000 sequencing platform. The adapter and low-quality nucleotide sequence in the fastq files were removed using the Trimmomatic v0.38 program [36] before proceeding with genome assembly using the conditions of a sliding window (4:20), average quality (Q30), and minimum read size (36 bp). Two methods were employed for long-read sequencing to compensate for quality and quantity. Initially, sequencing was carried out on MinION (Oxford Nanopore) followed by Single Molecule Real-Time (SMRT) sequencing on a PacBio Sequel platform. Genome assembly was performed with the long-read sequencing data using NextDenovo (v2.4.0) [37], and gaps in the assembly were covered with short-read Illumina data using NextPolish (v1.3.1). The completeness of the assembled genome was confirmed using the Benchmarking Universal Single-Copy Orthologs (BUSCO) v4.1.4 program [38] with nematoda_od10 as a database (n = 3131, https://busco-data.ezlab.org/v4/data/lineages/nematoda_odb10.2020-08-05.tar.gz, accessed on 5 September 2020). For transcriptome sequencing of HT and HS, mRNA was collected from three developmental stages (egg, J2, and female). RNA sequencing was carried out using the Illumina NovaSeq 6000 platform and PacBio Iso-Seq sequencing using the PacBio Sequel instrument. The transcript data were used for gene annotation, analysis of differentially expressed genes, and identification of alternative splicing isoforms. The raw data from genomic sequencing were submitted to NCBI-Sequence Read Archive with bioproject PRJNA1114691 for HT containing SRR29127238, SRR29127237, and SRR29127236. For HS, the data was submitted under bioproject PRJNA1111823, containing SRR29056519, SRR29056518, and SRR29056517.

### 4.3. Transcriptome Sequencing at Various Life Stages

For HT and HS transcriptome analysis, mRNA was collected from three developmental stages (the egg, J2, and female) and subjected to Illumina RNA-Seq and PacBio Iso-Seq sequencing. In the case of Iso-Seq, high-quality isoforms by sample were generated with the Iso-Seq3 pipeline. Each of the HT and HS isoforms was mapped to their corresponding genome using the minimap2 program (v2.25) [39] with a maximum intron size of 2000 nt (parameters: -ax splice:hq, -uf, and -G 2000) and the TAMA program (v1.0.3) [40] in non-capped mode with a threshold of 100 nt and coverage of 95% (parameters: -x no_cap, -d merge_dup, -e largest_ends, -a 100, and -c 95) was used to collapse the redundant or fragmented isoforms. The SQANTI3 program (v5.2.2) [41] (parameters: -polyA_motif_list, -short_reads, and -filter_mono_exonic TRUE) was used to identify and classify splice variants by comparing the alignment at the splicing junction between PacBio non-redundant isoforms and annotated genes of HT and HS in the presence of RNA-Seq reads. Of the SQANTI3 quality results, the isoforms showing a full-splice match (FSM), intron retention, exon skipping, and novel splicing junctions were selected for splice variants. For differentially expressed genes (DEGs) analysis, initially the RNA-Seq reads were aligned to *H. trifolii* and *H. schachtii* genomes using the HISAT2 (v2.1.0) program, and the read count of each transcript was calculated using the StringTie v1.3.4d program. The DEG analysis was performed using the DESeq (v1.38.0) package in R with the normalized read count in consideration of size factor and dispersion, and the genes were selected as differentially expressed genes (DEGs) when the absolute log_2_ fold change ≥ 1 and *p*-value ≤ 0.05. The RNA sequencing data was submitted to NCBI-Sequence Read Archive with bioproject PRJNA1114691 for HT containing SRR29127235 and SRR29127234, and for HS, the data was submitted under bioproject PRJNA1111823 containing SRR29056516 and SRR29056515.

### 4.4. Annotation and Gene Prediction

The characterization of repeat sequences was performed simultaneously using homology-based and de novo-based methods. The RepeatMasker program (v4.0.5) with Repbase was used for homology-based repeat analysis, and RepeatModeler (v1.0.4) was used for de novo repeat characterization. The repeat results from both analyses were combined, and the assembled genome was masked for repeat sequences prior to gene annotation. Two approaches were applied to produce the integrated gene models for each nematode. The first approach was ab initio gene prediction, and the second was an evidence-based gene prediction, which made use of transcriptome data obtained from HT and HS as well as protein sequences of the reference genome, *H. glycines*. A species-specific training model for gene prediction was created using the GeneMark-ES/EST (v4.48_3.60_lic) in the presence of RNA-Seq mapping information generated by HISAT2 (v2.1.0) [42], and then ab initio gene prediction was performed with AUGUSTUS (v3.2.2) [43] using the training model in BRAKER (v2.0) [44]. The assembled transcripts, PacBio isoforms, and Trinity de novo assembly (v2.11) were aligned to the assembled genome using blast in the PASA program (v2.4.1) to provide transcriptome evidence for gene annotation. Additionally, 32,171 protein sequences of *H. glycines* were mapped to the nematode genome using Exonerate (v 2.2.0), and the results were utilized for gene prediction. Finally, the results from ab initio gene prediction, transcript evidence, and protein homology were collectively considered to select a consensus gene model in EVidenceModeler (v1.1.1) [45] with the weights of 8, 10, and 8 for ab initio prediction, transcript evidence, and protein homology, respectively. For functional annotations of the selected gene models, a BLASTp (v2.9.0+) search was carried out against NCBI’s RefSeq protein and Uniprot databases with an E-value of 1 × 10^−3^. In addition, InterProScan (v5.38-76.0) was performed to add information of conserved protein domains, Gene Ontology (GO), and Kyoto Encyclopedia of Genes and Genomes (KEGG) to the final gene models. The annotated protein sequences of HT and HS were subjected to BLASTp against the protein-coding genes of *H. glycines* and *Caenorhabditis elegans*.

### 4.5. Genome Alignment, Gene Duplication, Synteny, and Ortholog Analysis

The HT and HS genomes were aligned to *H. glycines* (HG) using nucmer (v4.0) (default parameter), and mummerplot was used to visualize the alignment with the -filter option (select one-to-one best alignment). Genome duplication and synteny between HT, HS, and HG were identified using MCScanX analysis (v0.8) [46]. A collinear block was defined to have a match size equal to 5 (at least 5 genes present consecutively in both query and reference genomes) and a gap of less than 10 genes between collinear genes, which was 2.5 times more stringent than the default condition (default = 25). Orthofinder (v2.3.1) [47] (parameters: -M msa, -S diamond, -A mafft, -T fasttree, and -I 1.5) was used to carry out an orthologous gene search by performing pair wise homology search in a combined dataset of 244,350 protein sequences from HT, HS, HG, *Meloidogyne incognita*, *M. hapla*, *Globodera pallida*, *G. rostochiensis*, *Panagrellus redivivus*, *Strongyloides ratti*, and *Caenorhabditis elegans*.

### 4.6. Identification of Transcription Factors (TF), Effector Genes, and Genes Related to Horizontal Gene Transfer (HGT)

The annotated protein-coding genes of HT, HS, and HG were analyzed for TF using iTAK (v1.7) against the pfam Hidden Markov Model (HMM) library with an e-value cutoff of 1 × 10^−3^. The selected genes containing pfam domains were classified to TF families according to the AnimalTFDB (https://ngdc.cncb.ac.cn/databasecommons/database/id/8, accessed on 26 November 2019). For effector gene identification, Spla and Ryanodine receptor (SPRY) motif (PF00622)-containing proteins were selected from pfam results, and the other effector genes (tyrosinase, CLE or CLAVATA3/ESR, glutathione synthetase, and protein disulfide isomerase) were detected by BLAST against the UniProt database with 1 × 10^−4^ as a cutoff. For horizontal gene transfer (HGT) analysis, homology search of HT and HS protein sequences was carried out against the UniRef 90 protein database using the DIAMOND BLASTp program (v2.1.9.163) with a cutoff of 1 × 10^−3^ and 500 as the max number of hits. The Metazoan was selected for in-group taxa, and *Tylenchida* was used for taxonomic groups to exclude (EGP) from the HGT results in the AvP program. The alien index (AI) was calculated using the calculate_ai.py script provided by the AvP program (v1.0.3) [48]. The genes with AI > 0 were selected and clustered based on the shared BLAST hits (70%). The FASTA sequences for each group were aligned with MAFFT, and FastTree was used for phylogenetic inference in the AvP program. The genes classified as HGT and HGT_NT types were considered as HGT candidates.

### 4.7. Mitochondrial Genome Assembly and Annotation

The paired-end reads for HT and HS were generated using Illumina NovaSeq 6000, and quality trimming was performed using Trimmomatic (v0.38) with the conditions of a sliding window (4:20), average quality (Q20), and minimum read size (75 bp). The trimmed reads were mapped to the mitochondrial genome (NCBI accession HM640930.1) of *H. glycines* using the BWA-mem (v0.7.17) to extract the mitochondrial sequences, and the initial mitochondrial contigs were generated using the SPAdes (v3.11.1) assembler [49]. To construct a complete mitochondrial genome, a contig-joining by detecting the overlapping sequences, multiple contig extensions, and gap-fillings were performed manually using paired-end Illumina sequences in the Newbler (v2.9) program [50]. For protein-coding genes, ORF (open reading frame) prediction was carried out with the mitochondrial assembly and the predicted coding sequences (CDS) were edited in the Artems program (v17.0.1), followed by a BLAST search against the NCBI nr database to annotate gene function. For ribosomal RNA (rRNA) and transfer RNA (tRNA) detection, nematoda rRNA and tRNA data were downloaded from RNAcentral (https://rnacentral.org/, (accessed on 20 January 2025)) and a BLAST search was conducted on the assembled mitochondrial genome. The OGDraw program (v1.3.1) was used to visualize the mitochondrial genome and annotation. Ten nearest similar nematoda mitochondrial sequences to HS and HT were searched from NCBI, multiple sequence alignment was carried out using MAFFT (v7.407), and a phylogenetic tree was constructed with the NJ method in MEGA10 (v10.0.5) [51]. Used information of nucleotide sequences is as follows: *Punctodera chalcoensis* (GenBank accession no. HM640928.1), *Heterodera cardiolata* (HM640929.1), *Heterodera glycines* (HM640930.1), *Oesophagostomum columbianum* (KC715827.1), *Koerneria sudhausi* (NC_029233.1), *Caenorhabditis*_sp. (KY552903.1), *Uncinaria sanguinis* haplotype 45 (KF924757.1), *Litoditis* aff. marina (KR815450.1), *Globodera ellingtonae*_chromosome I (KU726971.1), and *Strongyloides stercoralis* (LC050212.1).

## Figures and Tables

**Table 1:** Whole genome assembly of the nematodes *H. schachtii* and *H. trifolii*.

	Total Scaffolds	Total Scaffold Length	N_50_ Length (bp)	Largest Scaffold Length (bp)	Shortest Scaffold Length (bp)	Average Scaffold Length (bp)
*H. trifolii*	1409	379,751,420	416,337	2,191,713	16,653	269,518
*H. schachtii*	458	183,156,359	782,540	3,671,058	9677	399,904

**Table 2:** Comparative genome statistics of cyst nematode genome assemblies.

Nematode Species Population	Size (Mb)	Number of Scaffolds	N_50_ (Mb)	BUSCO
*H. trifolii* (this study)	380 *	1409 *	0.4 *	C: 58.3% [S: 36.8%, D: 21.5%], F: 2.1%, M: 39.6%, n: 3131 *
*H. schachtii* (this study)	183 *	458 *	0.7 *	C: 57.8% [S: 55.3%, D: 2.5%], F: 2.7%, M: 39.5%, n: 3131 *
*H. glycines*, TN10	123 ^a^	738 ^a^	0.3 ^a^	C: 55.1% [S: 47.4%, D: 7.7%], F: 2.0%, M: 42.9%, n: 3131 ^a^
*H. schachtii*, IRS	190 ^b^	705 ^b^	0.5 ^b^	C: 86.3% [S: 80.8%, D: 5.5%], F: 7.1%, M: 6.6%, n: 255 ^b^
*H. schachtii*, Bonn	179 ^c^	395 ^c^	0.2 ^c^	C: 56.7% [S: 55.3%, D: 1.4%], F: 2.1%, M: 41.2%, n: 3131 ^c^

^a^ [16]; ^b^ [1]; ^c^ [18]; * our analyses. C: complete gene, S: single-copy gene, D: duplicated gene, M: missing gene, F: fragmented gene, n: number of gene.

## Data Availability

The original data presented in the study are openly available in the NCBI data base with accessions Bioprojects: PRJNA1114691 (HT) and PRJNA1111823 (HS). The genome assemblies are available at NCBI with accessions JBICBT000000000 (*Heterodera trifolii*) and JBICCN000000000 (*Heterodera schachtii*).

## Supplementary Materials

The following supporting information can be downloaded at: https://www.mdpi.com/article/10.3390/ijms26030948/s1.

## Abbreviations

HT: *Heterodera trifolii*; HS: *Heterodera schachtii*; HG: *Heterodera glycines*; PPN: plant-parasitic nematode; RKN: root-knot nematode; CN: cyst nematode; GM: genetically modified; TF: transcription factor; J2: juvenile stage 2.

## References

[1] van Steenbrugge J.J.M., van den Elsen S., Holterman M., Lozano-Torres J.L., Putker V., Thorpe P., Goverse A., Sterken M.G., Smant G., Helder J. (2023). Comparative genomics among cyst nematodes reveals distinct evolutionary histories among effector families and an irregular distribution of effector-associated promoter motifs. Mol. Ecol..

[2] Coyne D.L., Cortada L., Dalzell J.J., Claudius-Cole A.O., Haukeland S., Luambano N., Talwana H. (2018). Plant-Parasitic Nematodes and Food Security in Sub-Saharan Africa. Annu. Rev. Phytopathol..

[3] Nicol J.M., Turner S.J., Coyne D.L., den Nijs L., Hockland S., Maafi Z.T., Jones J., Gheysen G., Fenoll C. (2011). Current Nematode Threats to World Agriculture. Genomics and Molecular Genetics of Plant-Nematode Interactions.

[4] Bird D.M., Jones J.T., Opperman C.H., Kikuchi T., Danchin E.G.J. (2015). Signatures of adaptation to plant parasitism in nematode genomes. Parasitology.

[5] Vieira P., Gleason C. (2019). Plant-parasitic nematode effectors—Insights into their diversity and new tools for their identification. Curr. Opin. Plant Biol..

[6] Abd-Elgawad M.M., Askary T.H. (2015). Impact of phytonematodes on agriculture economy. Biocontrol Agents of Phytonematodes.

[7] Kim J., Kim T., Lee Y.-C., Chun J.-Y., Kern E.M.A., Jung J., Park J.-K. (2016). Characterization of 15 microsatellite loci and genetic analysis of *Heterodera schachtii* (Nematoda: Heteroderidae) in South Korea. Biochem. Syst. Ecol..

[8] Mwamula A.O., Ko H.-R., Kim Y., Kim Y.H., Lee J.-K., Lee D.W. (2018). Morphological and Molecular Characterization of *Heterodera schachtii* and the Newly Recorded Cyst Nematode, *H*. trifolii Associated with Chinese Cabbage in Korea. Plant Pathol. J..

[9] Ajjappala H., Chung H.Y., Sim J.-S., Choi I., Hahn B.-S. (2015). Disruption of prefoldin-2 protein synthesis in root-knot nematodes via host-mediated gene silencing efficiently reduces nematode numbers and thus protects plants. Planta.

[10] Kahn T.W., Duck N.B., McCarville M.T., Schouten L.C., Schweri K., Zaitseva J., Daum J. (2021). A *Bacillus thuringiensis* Cry protein controls soybean cyst nematode in transgenic soybean plants. Nat. Commun..

[11] Niblack T.L., Lambert K.N., Tylka G.L. (2006). A Model Plant Pathogen from the Kingdom Animalia: *Heterodera glycines*, the Soybean Cyst Nematode. Annu. Rev. Phytopathol..

[12] Koutsovoulos G.D., Marques E., Arguel M.-J., Duret L., Machado A.C.Z., Carneiro R.M.D.G., Kozlowski D.K., Bailly-Bechet M., Castagnone-Sereno P., Albuquerque E.V.S. (2020). Population genomics supports clonal reproduction and multiple independent gains and losses of parasitic abilities in the most devastating nematode pest. Evol. Appl..

[13] Koutsovoulos G.D., Poullet M., Elashry A., Kozlowski D.K.L., Sallet E., Da Rocha M., Perfus-Barbeoch L., Martin-Jimenez C., Frey J.E., Ahrens C.H. (2020). Genome assembly and annotation of *Meloidogyne enterolobii*, an emerging parthenogenetic root-knot nematode. Sci. Data.

[14] Phan N.T., Orjuela J., Danchin E.G.J., Klopp C., Perfus-Barbeoch L., Kozlowski D.K., Koutsovoulos G.D., Lopez-Roques C., Bouchez O., Zahm M. (2020). Genome structure and content of the rice root-knot nematode (*Meloidogyne graminicola*). Ecol. Evol..

[15] Montarry J., Mimee B., Danchin E.G.J., Koutsovoulos G.D., Ste-Croix D.T., Grenier E. (2021). Recent Advances in Population Genomics of Plant-Parasitic Nematodes. Phytopathology.

[16] Masonbrink R., Maier T.R., Muppirala U., Seetharam A.S., Lord E., Juvale P.S., Schmutz J., Johnson N.T., Korkin D., Mitchum M.G. (2019). The genome of the soybean cyst nematode (*Heterodera glycines*) reveals complex patterns of duplications involved in the evolution of parasitism genes. BMC Genom..

[17] Masonbrink R.E., Maier T.R., Hudson M., Severin A., Baum T. (2021). A chromosomal assembly of the soybean cyst nematode genome. Mol. Ecol. Resour..

[18] Siddique S., Radakovic Z.S., Hiltl C., Pellegrin C., Baum T.J., Beasley H., Bent A.F., Chitambo O., Chopra D., Danchin E.G.J. (2022). The genome and life stage-specific transcriptomes of a plant-parasitic nematode and its host reveal susceptibility genes involved in trans-kingdom synthesis of vitamin B5. Nat. Commun..

[19] Kikuchi T., Eves-van den Akker S., Jones J.T. (2017). Genome Evolution of Plant-Parasitic Nematodes. Annu. Rev. Phytopathol..

[20] Jones J.T., Haegeman A., Danchin E.G.J., Gaur H.S., Helder J., Jones M.G.K., Kikuchi T., Manzanilla-López R., Palomares-Rius J.E., Wesemael W.M.L. (2013). Top 10 plant-parasitic nematodes in molecular plant pathology. Mol. Plant Pathol..

[21] Cotton J.A., Lilley C.J., Jones L.M., Kikuchi T., Reid A.J., Thorpe P., Tsai I.J., Beasley H., Blok V., Cock P.J.A. (2014). The genome and life-stage specific transcriptomes of *Globodera pallida* elucidate key aspects of plant parasitism by a cyst nematode. Genome Biol..

[22] Ma X., Agudelo P., Richards V.P., Baeza J.A. (2022). Genome survey sequencing of the phyto-parasitic nematode *Hoplolaimus galeatus*. PeerJ.

[23] Siddique S., Coomer A., Baum T., Williamson V.M. (2022). Recognition and Response in Plant–Nematode Interactions. Annu. Rev. Phytopathol..

[24] de Jong L., Meng Y., Dent J., Hekimi S. (2004). Thiamine pyrophosphate biosynthesis and transport in the nematode *Caenorhabditis elegans*. Genetics.

[25] Price J.A., Ali M.F., Major L.L., Smith T.K., Jones J.T. (2023). An eggshell-localised annexin plays a key role in the coordination of the life cycle of a plant-parasitic nematode with its host. PLOS Pathog..

[26] Coghlan A. (2005). Nematode genome evolution. WormBook.

[27] Craig J.P., Bekal S., Hudson M., Domier L., Niblack T., Lambert K.N. (2008). Analysis of a horizontally transferred pathway involved in vitamin B6 biosynthesis from the soybean cyst nematode *Heterodera glycines*. Mol. Biol. Evol..

[28] Craig J.P., Bekal S., Niblack T., Domier L., Lambert K.N. (2009). Evidence for horizontally transferred genes involved in the biosynthesis of vitamin B(1), B(5), and B(7) in *Heterodera glycines*. J. Nematol..

[29] Eves-van den Akker S., Laetsch D.R., Thorpe P., Lilley C.J., Danchin E.G.J., Da Rocha M., Rancurel C., Holroyd N.E., Cotton J.A., Szitenberg A. (2016). The genome of the yellow potato cyst nematode, *Globodera rostochiensis*, reveals insights into the basis of parasitism and virulence. Genome Biol..

[30] Sibley C.R., Blazquez L., Ule J. (2016). Lessons from non-canonical splicing. Nat. Rev. Genet..

[31] Zou H., Jakovlić I., Chen R., Zhang D., Zhang J., Li W.-X., Wang G.-T. (2017). The complete mitochondrial genome of parasitic nematode *Camallanus cotti*: Extreme discontinuity in the rate of mitogenomic architecture evolution within the Chromadorea class. BMC Genom..

[32] Adams P.E., Bubrig L.T., Fierst J.L. (2020). Genome Evolution: On the Road to Parasitism. Curr. Biol..

[33] Eves-van den Akker S. (2021). Plant–nematode interactions. Curr. Opin. Plant Biol..

[34] Flores M. (2020). A Modified Protocol for Rapid HMW DNA Isolation from Plant Tissues Using CTAB.

[35] Peloquin J.J., Bird D.M., Platzer E.G. (1993). Rapid miniprep isolation of mitochondrial DNA from metacestodes, and free-living and parasitic nematodes. J. Parasitol..

[36] Bolger A.M., Lohse M., Usadel B. (2014). Trimmomatic: A flexible trimmer for Illumina sequence data. Bioinformatics.

[37] Hu J., Wang Z., Sun Z., Hu B., Ayoola A.O., Liang F., Li J., Sandoval J.R., Cooper D.N., Ye K. (2024). NextDenovo: An efficient error correction and accurate assembly tool for noisy long reads. Genome Biol..

[38] Manni M., Berkeley M.R., Seppey M., Zdobnov E.M. (2021). BUSCO: Assessing Genomic Data Quality and Beyond. Curr. Protoc..

[39] Li H. (2018). Minimap2: Pairwise alignment for nucleotide sequences. Bioinformatics.

[40] Kuo R.I., Cheng Y., Zhang R., Brown J.W.S., Smith J., Archibald A.L., Burt D.W. (2020). Illuminating the dark side of the human transcriptome with long read transcript sequencing. BMC Genom..

[41] Pardo-Palacios F.J., Arzalluz-Luque A., Kondratova L., Salguero P., Mestre-Tomás J., Amorín R., Estevan-Morió E., Liu T., Nanni A., McIntyre L. (2024). SQANTI3: Curation of long-read transcriptomes for accurate identification of known and novel isoforms. Nat. Methods.

[42] Kim D., Paggi J.M., Park C., Bennett C., Salzberg S.L. (2019). Graph-based genome alignment and genotyping with HISAT2 and HISAT-genotype. Nat. Biotechnol..

[43] Keller O., Kollmar M., Stanke M., Waack S. (2011). A novel hybrid gene prediction method employing protein multiple sequence alignments. Bioinformatics.

[44] Brůna T., Hoff K.J., Lomsadze A., Stanke M., Borodovsky M. (2021). BRAKER2: Automatic eukaryotic genome annotation with GeneMark-EP+ and AUGUSTUS supported by a protein database. NAR Genom. Bioinform..

[45] Haas B.J., Salzberg S.L., Zhu W., Pertea M., Allen J.E., Orvis J., White O., Buell C.R., Wortman J.R. (2008). Automated eukaryotic gene structure annotation using EVidence Modeler and the Program to Assemble Spliced Alignments. Genome Biol..

[46] Wang Y., Tang H., Debarry J.D., Tan X., Li J., Wang X., Lee T.H., Jin H., Marler B., Guo H. (2012). MCScanX: A toolkit for detection and evolutionary analysis of gene synteny and collinearity. Nucleic Acids Res..

[47] Emms D.M., Kelly S. (2019). OrthoFinder: Phylogenetic orthology inference for comparative genomics. Genome Biol..

[48] Koutsovoulos G.D., Noriot S.G., Bailly-Bechet M., Danchin E.G.J., Rancurel C. (2022). AvP: A software package for automatic phylogenetic detection of candidate horizontal gene transfers. PLoS Comput. Biol..

[49] Bankevich A., Nurk S., Antipov D., Gurevich A.A., Dvorkin M., Kulikov A.S., Lesin V.M., Nikolenko S.I., Pham S., Prjibelski A.D. (2012). SPAdes: A new genome assembly algorithm and its applications to single-cell sequencing. J. Comput. Biol..

[50] Silva G.G., Dutilh B.E., Matthews T.D., Elkins K., Schmieder R., Dinsdale E.A., Edwards R.A. (2013). Combining de novo and reference-guided assembly with scaffold_builder. Source Code Biol. Med..

[51] Kumar S., Stecher G., Li M., Knyaz C., Tamura K. (2018). MEGA X: Molecular Evolutionary Genetics Analysis across Computing Platforms. Mol. Biol. Evol..

