# Supplementary material for: Genomic and Transcriptomic Analysis of the Polyploidy Cyst Nematode, Heterodera trifolii, and Heterodera schachtii
Source: Int J Mol Sci. 2025 Jan 23;26(3):948. doi: 10.3390/ijms26030948 (PMC11817004; doi:10.3390/ijms26030948)
Supplement: Supplementary file 1 [file ijms-26-00948-s001.zip › Supplementary revised file.pdf]

**Supplementary tables**

Table S1. Trimmed sequencing data for HT and HS using three sequencing methods

| | Ilumina sequencing | | Nanopore sequencing | | PacBio sequencing | |
|---|---|---|---|---|---|---|
| | Total read length (bp) | No. of reads | Total read length (bp) | No. of reads | Total read length (bp) | No. of reads |
| *H. trifolii* | 63,522,624,132 | 546,144,246 | 6,400,735,226 | 737,512 | 30,422,696,271 | 2,741,662 |
| *H.schachtii* | 36,783,789,522 | 291,708,128 | 2,642,252,532 | 355,026 | 28,996,119,854 | 2,832,361 |

Table S2. Long reads: mean read length & $N_{50}$ read length

| | Oxford nanopore sequencing | | PacBio Sequencing | |
|---|---|---|---|---|
| | Mean read length | $N_{50}$ read length | Mean read length | $N_{50}$ read length |
| *H. trifolii* | 8822 | 15,338 | 11,096 | 18,750 |
| *H. schachtii* | 7442 | 10,568 | 10,237 | 17,250 |

Table S3. Repeats composition of the cyst nematode *H. trifolii*

| Class | Element | Count | Size (bp) | % in total repeats | % in genome |
|---|---|---|---|---|---|
| DNA transposons | DNA/Crypton | 1669 | 310,796 | 0.37 | 0.08 |
| | DNA/EnSpm | 5311 | 1,442,406 | 1.18 | 0.38 |
| | DNA/Ginger | 396 | 83,117 | 0.09 | 0.02 |
| | DNA/Harbinger | 152 | 51,772 | 0.03 | 0.01 |
| | DNA/hAT | 28,079 | 7,647,311 | 6.22 | 2.01 |
| | DNA/Mariner | 2857 | 736,340 | 0.63 | 0.19 |
| | DNA/Maverick | 6950 | 11,354,195 | 1.54 | 2.99 |
| | DNA/Merlin | 564 | 123,274 | 0.13 | 0.03 |
| | DNA/MuDR | 5382 | 1,798,129 | 1.19 | 0.47 |
| | DNA/Sola | 14,970 | 3,460,712 | 3.32 | 0.91 |
| | DNA/unknown | 6554 | 1,636,790 | 1.45 | 0.43 |
| Total DNA transposons | | 72,884 | 28,644,842 | 16.16 | 7.54 |
| LTR | LTR/Copia | 230 | 152,166 | 0.05 | 0.04 |
| | LTR/ERV | 1287 | 402,573 | 0.29 | 0.11 |
| | LTR/Gypsy | 16,646 | 11,047,161 | 3.69 | 2.91 |
| | LTR/Pao | 2956 | 2,779,701 | 0.66 | 0.73 |
| | LTR/unknown | 1034 | 347,719 | 0.23 | 0.09 |
| Total LTR | | 22,153 | 14,729,320 | 4.91 | 3.88 |
| LINE | LINE/CR1 | 3479 | 1,762,006 | 0.77 | 0.46 |
| | LINE/Jockey | 1627 | 296,967 | 0.36 | 0.08 |
| | LINE/L1 | 3412 | 1,006,299 | 0.76 | 0.26 |
| | LINE/L2 | 110 | 6168 | 0.02 | 0 |

| | | | | | |
|---|---|---|---|---|---|
| | LINE/others | 1793 | 634,115 | 0.4 | 0.17 |
| | LINE/R1 | 171 | 79,276 | 0.04 | 0.02 |
| | LINE/RTE | 68 | 4036 | 0.02 | 0 |
| Total LINE | | 10,660 | 3,788,867 | 2.36 | 1 |
| SINE | SINE/MIR | 30 | 1718 | 0.01 | 0 |
| | SINE/rRNA-derived | 430 | 49,114 | 0.1 | 0.01 |
| | SINE/tRNA-derived | 62 | 4953 | 0.01 | 0 |
| | SINE/unknown | 325 | 30,438 | 0.07 | 0.01 |
| Total SINE | | 847 | 86,223 | 0.19 | 0.02 |
| Others | Others/Helitron | 7775 | 2,710,216 | 1.72 | 0.71 |
| | Others/Satellite | 297 | 63,701 | 0.07 | 0.02 |
| | Others/unknown | 285,726 | 87,387,226 | 63.33 | 23.01 |
| | Others/Simple_repeat | 50,807 | 1,697,645 | 11.26 | 0.45 |
| Total Others | | 344,605 | 91,858,788 | 76.38 | 24.19 |
| Sum | | 451,149 | 139,108,040 | 100 | 36.63 |

Table S4. Repeats composition of the cyst nematode *H. schachtii*

| Class | Element | Count | Size (bp) | % in total repeats | % in genome |
|---|---|---|---|---|---|
| DNA transposons | DNA/Crypton | 404 | 61,546 | 0.19 | 0.03 |
| | DNA/EnSpm | 2453 | 643,985 | 1.13 | 0.35 |
| | DNA/Harbinger | 73 | 35,031 | 0.03 | 0.02 |
| | DNA/hAT | 11,812 | 2,858,860 | 5.44 | 1.56 |
| | DNA/Mariner | 4105 | 1,788,001 | 1.89 | 0.98 |
| | DNA/Maverick | 4357 | 5,905,226 | 2.01 | 3.22 |
| | DNA/Merlin | 364 | 72,628 | 0.17 | 0.04 |
| | DNA/MuDR | 2499 | 812,245 | 1.15 | 0.44 |
| | DNA/Sola | 4082 | 749,819 | 1.88 | 0.41 |
| | DNA/unknown | 4112 | 824,624 | 1.89 | 0.45 |
| Total DNA transposons | | 34,261 | 13,751,965 | 15.77 | 7.51 |
| LTR | LTR/Copia | 1250 | 411,762 | 0.58 | 0.22 |
| | LTR/ERV | 149 | 25,824 | 0.07 | 0.01 |
| | LTR/Gypsy | 7176 | 5,020,800 | 3.3 | 2.56 |
| | LTR/Ngaro | 71 | 17,462 | 0.03 | 0.01 |
| | LTR/Pao | 1882 | 1,754,613 | 0.87 | 0.96 |
| | LTR/unknown | 346 | 45,425 | 0.16 | 0.02 |
| Total LTR | | 10,874 | 7,275,886 | 5.01 | 3.97 |
| LINE | LINE/CR1 | 1394 | 667,722 | 0.64 | 0.36 |
| | LINE/CRE | 125 | 24,910 | 0.06 | 0.01 |
| | LINE/DRE | 660 | 442,042 | 0.3 | 0.24 |
| | LINE/Jockey | 5 | 360 | 0 | 0 |
| | LINE/L1 | 1312 | 340,818 | 0.6 | 0.19 |

| Class | Element | Count | Size (bp) | % in total repeats | % in genome |
|---|---|---|---|---|---|
| | LINE/L2 | 257 | 81,399 | 0.12 | 0.04 |
| | LINE/others | 9 | 658 | 0 | 0 |
| | LINE/R1 | 764 | 237,079 | 0.35 | 0.13 |
| | LINE/RTE | 30 | 1717 | 0.01 | 0 |
| Total LINE | | 4556 | 1,795,705 | 2.1 | 0.98 |
| SINE | SINE/MIR | 13 | 939 | 0.01 | 0 |
| | SINE/rRNA-derived | 253 | 62,160 | 0.12 | 0.03 |
| | SINE/tRNA-derived | 26 | 2170 | 0.02 | 0 |
| | SINE/unknown | 100 | 43,011 | 0.05 | 0.02 |
| Total SINE | | 392 | 108,280 | 0.18 | 0.06 |
| Others | Others/Helitron | 5225 | 2,198,554 | 2.41 | 1.20 |
| | Others/unknown | 134,806 | 40,279,652 | 62.07 | 21.99 |
| | Others/Simple_repeat | 27,075 | 1,007,659 | 12.47 | 0.55 |
| Total Others | | 167,106 | 43,485,865 | 76.94 | 23.74 |
| Sum | | 217,189 | 66,417,701 | 100 | 36.26 |

Table S5. Simple sequence repeats (SSR) composition of the cyst nematodes HS and HT

| Nematode | Di-nucleotide | Tri-nucleotides | Tetra-nucleotide | Penta-nucleotide | Hexa-nucleotide |
|---|---|---|---|---|---|
| *H. trifolii* | 4929 (13.6%) | 11,748 (32.4%) | 10,849 (29.9%) | 5300 (14.6%) | 3462 (9.5%) |
| *H. schachtii* | 2682 (14.5%) | 5434 (29.4%) | 5572 (30.2%) | 3003 (16.3%) | 1782 (9.6%) |

Table S6. Gene prediction from the assembled genomes of cyst nematodes HS and HT

| Species | Genes | Exons | Introns | Exon /gene | Avg. exon (bp) | Avg. intron (bp) | Avg. transcript length (bp) | Avg. Gene length (bp) |
|---|---|---|---|---|---|---|---|---|
| *H. trifolii* | 40,186 | 309,259 | 269,073 | 7.7 | 167.9 | 244.6 | 1292.1 | 2929.9 |
| *H. schachtii* | 18,227 | 155,518 | 137,291 | 8.5 | 160 | 234 | 1367 | 3132 |

Table S7. Synteny analysis of the genomes of HS and HT compared to the reference genome of *H. glycines*

| Comparison | Synteny blocks | Collinear genes | % Genome collinearity |
|---|---|---|---|
| HT (vs HG) | 1534 | 22,594 | 64.7% |
| HS (vs HG) | 699 | 12,741 | 53.2% |

Table S8. Duplication patterns in the genomes of cyst nematodes HT and HS

| | *H. glycine* | | *H. trifolii* | | *H. schachtiii* | |
|---|---|---|---|---|---|---|
| Gene type | No. of genes | % | No. of genes | % | No. of genes | % |
| Singleton | 5716 | 19.3 | 2581 | 6.9 | 4335 | 23.8 |
| Dispersed | 10,956 | 36.9 | 13,104 | 32.2 | 8917 | 48.9 |
| Proximal | 3634 | 12.2 | 2927 | 7.2 | 1925 | 10.6 |
| Tandem | 1680 | 5.7 | 2394 | 5.9 | 1658 | 9.1 |
| WGD/segmental | 7693 | 26.9 | 19,180 | 47.8 | 1392 | 7.6 |
| Total | 29,679 | | 40,186 | | 18,227 | |

Table S9. Statistics of orthologous genes detected in the genomes of cyst nematodes HT and HS

| **Description** | **HT** | **HS** | **HG** |
|---|---|---|---|
| Total predicted genes | 40,186 | 18,227 | 29,679 |
| Number of orthologous genes (in orthogroup) | 23,668 | 6,286 | 19,067 |
| Percentage (%) genes in orthogroup | 58.9 | 89.0 | 64.2 |
| Unassigned genes | 16,518 | 1,941 | 10,612 |
| Percentage (%) unassigned genes | 41.1 | 11.0 | 35.8 |
| Species-specific orthogroups | 18 | 1 | 26 |
| Genes in species-specific orthogroups | 91 | 2 | 93 |
| % genes in species-specific orthogroups | 0.2 | 0 | 0.3 |

Table S10. ISO-Seq data obtained by sequencing performed at different stages of the nematode life cycle

| Species | Type | Total reads | Total read bases | Mean Read Length | Overall assembly (bp) | No. of isoforms |
|---|---|---|---|---|---|---|
| *H. trifolii* | Egg | 102,057 | 105,805,985 | 1037 | 329,202,811 | 246,325 |
| | J2 | 86,250 | 77,270,479 | 896 | | |
| | Female | 94,597 | 154,146,399 | 1630 | | |
| *H. schachtii* | Egg | 35,073 | 47,834,827 | 1364 | 143,170,623 | 83,761 |
| | J2 | 21,902 | 58,164,189 | 2655 | | |
| | Female | 37,374 | 39,420,267 | 1055 | | |

Table S11. RNA sequencing statistics

| Species | Sample | Read No. | Total length (Mb) | % retained |
|---|---|---|---|---|
| | Egg | 63,943,172 | 6272 | 93 |
| *H. trifolii* | J2 | 56,182,454 | 5522 | 94.4 |
| | Female | 47,452,196 | 4687 | 93.1 |
| Total | | 167,577,822 | 16,481 | 93.5 |
| *H. schachtii* | Egg | 56,111,240 | 5639 | 97.2 |
| | J2 | 68,220,654 | 6839 | 98.3 |
| | Female | 60,036,520 | 6023 | 96.9 |
| Total | | 184,368,414 | 18,501 | 97.5 |

Table S12. Statistics of spliced and novel isoform s predicted in the genomes of HT and HS at various stages of their life cycle

| Samples | No. spliced isoforms | Avg. spliced isoform length (bp) | No. spliced isoform genes | (Spliced) isoform/ genes | No. novel isoforms | Avg. novel isoform length (bp) | No. novel isoform genes | (novel) isoforms /genes |
|---|---|---|---|---|---|---|---|---|
| HT Egg stage | 5125 | 1471.8 | 4089 | 1.25 | 2334 | 1587.4 | 1787 | 1.31 |
| HT J2 stage | 3917 | 1323.1 | 3132 | 1.25 | 1915 | 1454.9 | 1469 | 1.3 |
| HT Female stage | 6671 | 2072.9 | 4591 | 1.45 | 3604 | 2345 | 2260 | 1.59 |
| Average | 5238 | 1622.6 | 3937 | 1.32 | 2618 | 1795.8 | 1839 | 1.4 |
| HS Egg stage | 3964 | 1796 | 2907 | 1.36 | 1921 | 2016.7 | 1322 | 1.45 |
| HS J2 stage | 3254 | 2541.3 | 1888 | 1.72 | 2128 | 2635.7 | 1138 | 1.87 |
| HS Female stage | 3348 | 1493.3 | 2874 | 1.16 | 1201 | 1597 | 1000 | 1.2 |
| Average | 3522 | 1944.5 | 2556 | 1.41 | 1750 | 2083.1 | 1153 | 1.51 |

Table S13. Functional gene annotation of the genes predicted in the two cyst nematode genomes of HT and HS

| Classified gene function | *H. trifolii* | | *H. schachtii* | |
|---|---|---|---|---|
| | Gene count | Percent | Gene count | Percent |
| Total known protein (refseq vertebrate + uniprot + *C. elegans* hit) | 28,398 | 70.70% | 13,691 | 75.11% |
| Total uncharacterized protein | 11,788 | 29.30% | 4,536 | 24.89% |
| BLASTP with *H. glycine* protein & RNAseq expression | 8777 | | 3880 | |
| BLASTP with *H. glycine* protein & no RNAseq expression | 3011 | | 656 | |
| Total gene model | 40,186 | 100% | 18,227 | 100% |

Table S14. Gene ontology enrichment analysis of the syntenic genes in the nematodes HS and HT.

| Gene type | GO category | GO-ID | p-value | Corr. p-value | Description |
|---|---|---|---|---|---|
| HG, HS & HT | BP | 9228 | 2.93E-04 | 3.78E-02 | Thiamin biosynthetic process |
| | BP | 6772 | 2.93E-04 | 3.78E-02 | Thiamin metabolic process |
| | MF | 4417 | 2.42E-04 | 4.15E-02 | Hydroxyethylthiazole kinase activity |
| HG & HT | BP | 5975 | 2.48E-05 | 1.04E-02 | Carbohydrate metabolic process |
| | BP | 51179 | 4.94E-05 | 1.04E-02 | Localization |
| | BP | 51234 | 6.36E-05 | 1.04E-02 | Establishment of localization |
| | BP | 6810 | 6.36E-05 | 1.04E-02 | Transport |
| | BP | 6396 | 6.64E-05 | 1.04E-02 | RNA processing |
| | BP | 16052 | 1.23E-04 | 1.46E-02 | Carbohydrate catabolic process |
| | BP | 44281 | 1.30E-04 | 1.46E-02 | Small molecule metabolic process |
| | BP | 15672 | 1.96E-04 | 1.93E-02 | Monovalent inorganic cation transport |
| | BP | 6096 | 2.68E-04 | 2.34E-02 | Glycolysis |
| | BP | 6022 | 3.21E-04 | 2.52E-02 | Aminoglycan metabolic process |
| | BP | 31123 | 4.11E-04 | 2.94E-02 | RNA 3'-end processing |
| | BP | 55114 | 5.63E-04 | 3.46E-02 | Oxidation reduction |
| | BP | 5976 | 5.73E-04 | 3.46E-02 | Polysaccharide metabolic process |
| | BP | 6030 | 7.06E-04 | 3.96E-02 | Chitin metabolic process |
| | BP | 9308 | 9.46E-04 | 4.95E-02 | Amine metabolic process |
| | MF | 42302 | 1.67E-08 | 9.37E-06 | Structural constituent of cuticle |
| | MF | 5198 | 5.90E-06 | 1.65E-03 | Structural molecule activity |
| | MF | 16491 | 9.94E-05 | 1.86E-02 | Oxidoreductase activity |
| HG & HS | CC | 45275 | 1.48E-03 | 3.84E-02 | Respiratory chain complex III |
| | CC | 5750 | 1.48E-03 | 3.84E-02 | Mitochondrial respiratory chain complex III |

Table S15. Functional annotation of highly expressed genes of *H. trifolii*

| GO | No. of genes | KEGG | No. of genes | Pfam | No. of genes |
|---|---|---|---|---|---|
| Protein binding | 3412 | Glutathione metabolism | 217 | BTB/POZ domain | 840 |
| ATP binding | 1594 | | 214 | SPRY domain | 523 |
| Integral to membrane | 1250 | Starch and sucrose metabolism | 99 | MATH domain | 511 |
| DNA binding | 950 | Purine metabolism | 96 | BTB And C-terminal Kelch | 497 |
| Nucleic acid binding | 907 | Glycolysis;Gluconeogenesis | 82 | Integrase core domain | 486 |
| Membrane | 723 | Amino sugar and nucleotide sugar metabolism | 74 | Protein kinase domain | 473 |
| Proteolysis | 721 | Fructose and mannose metabolism | 60 | Ankyrin repeats (3 copies) | 299 |
| Protein amino acid phosphorylation | 685 | Galactose metabolism | 59 | Serpentine type 7TM GPCR chemoreceptor Srsx | 296 |
| Protein kinase activity | 684 | Aminoacyl-tRNA biosynthesis | 53 | AAA domain | 273 |
| Zinc ion binding | 629 | mTOR signaling pathway | 53 | Ring finger domain | 270 |
| DNA integration | 601 | PI3K-Akt signaling pathway | 53 | Collagen triple helix repeat (20 copies) | 239 |
| Transmembrane transport | 489 | Human cytomegalovirus infection | 53 | Nematode cuticle collagen N-terminal domain | 233 |
| Oxidation reduction | 489 | Human papillomavirus infection | 53 | Homeodomain | 229 |
| Nucleus | 452 | Human immunodeficiency virus 1 infection | 53 | DNA polymerase type B, organellar and viral | 229 |
| Regulation of transcription, DNA-dependent | 448 | Pentose and glucuronate interconversions | 52 | Transcription factor TFIID (or TATA-binding protein, TBP) | 192 |
| Transcription factor activity | 319 | Thermogenesis | 49 | Ubiquitin family | 186 |
| Calcium ion binding | 297 | Relaxin signaling pathway | 49 | Eukaryotic glutathione synthase, ATP binding domain | 185 |
| Nucleotide binding | 291 | Ether lipid metabolism | 46 | Zinc finger, C4 type (two domains) | 183 |
| DNA replication | 258 | Lysine degradation | 43 | 7 transmembrane receptor (rhodopsin family) | 182 |
| Sequence-specific DNA binding | 244 | Glycerolipid metabolism | 42 | Protein tyrosine kinase | 178 |

Table S16. Functional annotation of highly expressed genes of *H. schachtii*

| GO | No. of genes | KEGG | No. of genes | Pfam | No. of genes |
|---|---|---|---|---|---|
| Protein binding | 1866 | Glutathione metabolism | 153 | BTB/POZ domain | 412 |
| ATP binding | 1352 | Cysteine and methionine metabolism | 123 | ABC transporter | 281 |
| Integral to membrane | 1071 | Purine metabolism | 114 | LysR substrate binding domain | 268 |
| Membrane | 1009 | Aminoacyl-tRNA biosynthesis | 91 | MATH domain | 262 |
| Oxidation reduction | 995 | Carbon fixation pathways in prokaryotes | 70 | Protein kinase domain | 250 |
| Regulation of transcription, DNA-dependent | 980 | Starch and sucrose metabolism | 65 | Bacterial regulatory helix-turn-helix protein, lysR family | 249 |
| Transmembrane transport | 893 | Glycolysis;Gluconeogenesis | 60 | SPRY domain | 240 |
| DNA binding | 821 | Propanoate metabolism | 55 | BTB And C-terminal Kelch | 233 |
| Transcription factor activity | 642 | Pyrimidine metabolism | 54 | Major Facilitator Superfamily | 219 |
| Nucleic acid binding | 542 | Pyruvate metabolism | 52 | Integrase core domain | 192 |
| Catalytic activity | 537 | Oxidative phosphorylation | 47 | Ankyrin repeats (3 copies) | 165 |
| Oxidoreductase activity | 536 | Lysine degradation | 46 | Serpentine type 7TM GPCR chemoreceptor Srsx | 160 |
| Proteolysis | 441 | Methane metabolism | 45 | Response regulator receiver domain | 144 |
| Protein kinase activity | 342 | Pantothenate and CoA biosynthesis | 44 | Tripartite tricarboxylate transporter family receptor | 139 |
| Protein amino acid phosphorylation | 341 | Amino sugar and nucleotide sugar metabolism | 43 | AAA domain | 134 |
| Zinc ion binding | 329 | Ether lipid metabolism | 42 | DNA polymerase type B, organellar and viral | 119 |
| Transmembrane transporter activity | 307 | Folate biosynthesis | 41 | Ring finger domain | 119 |
| Hydrolase activity | 294 | Tryptophan metabolism | 41 | Binding-protein-dependent transport system inner membrane component | 117 |
| ATPase activity | 290 | Citrate cycle (TCA cycle) | 39 | Histidine kinase-, DNA gyrase B-, and HSP90-like ATPase | 116 |
| DNA integration | 244 | Glycerophospholipid metabolism | 39 | 7 transmembrane receptor (rhodopsin family) | 111 |

Table S17. BLAST analysis of species-specific orthologous genes in HT, HS and HG

| Genome | Orthogroup(s) | Number of genes in orthogroup | BLAST results | Respective genes |
|---|---|---|---|---|
| HT | 18 | 91 | Uncharacterized protein F54H12.2 | 31 |
| | | | Lon protease homolog | 3 |
| | | | Receptor-type guanylate cyclase gcy-13 | 3 |
| | | | PDZ domain-containing protein C52A11.3 | 3 |
| | | | Regulator of nonsense transcripts 1 | 3 |
| | | | Putative uncharacterized transposon-derived protein F54H12.3 | 5 |
| | | | no blast hit | 43 |
| HS | 1 | 2 | BTB/POZ domain-containing protein 6-A | 2 |
| HG | 26 | 93 | Testis-specific serine/threonine-protein kinase 6 | 8 |
| | | | Sentrin-specific protease 1, Putative ubiquitin-like-specific protease 1B | 4 |
| | | | Uncharacterized protein K02A2.6 | 7 |
| | | | Pro-Pol polyprotein | 1 |
| | | | Retrovirus-related Pol polyprotein from transposon 17.6 | 2 |
| | | | no blast hit | 71 |

Table S18. Top ten results of the gene ontology analysis of the segmental duplication genes in the nematodes HS and HT. Number of genes identified in each segment is given in parentheses.

| Nematode | Biological process (BP) | Cellular components (CC) | Molecular function (MF) |
|---|---|---|---|
| *H. trifolii* | Transmembrane transport (75) | Integral to membrane (367) | Protein binding (1695) |
| | Proteolysis (70) | Membrane (114) | Nucleic acid binding (390) |
| | Protein amino acid glycosylation (63) | Nucleus (51) | Protein kinase activity (379) |
| | Sensory perception of chemical stimulus (54) | Extracellular space (44) | DNA binding (257) |
| | Ubiquitin-dependent protein catabolic process (37) | Extracellular region (41) | Transcription factor activity (189) |
| | Lipid metabolic process (37) | Nucleosome (41) | Structural constituent of cuticle (168) |
| | DNA integration (30) | Mitochondrion (14) | ATP binding (145) |
| | Signal transduction (30) | Endoplasmic reticulum membrane (13) | Calcium ion binding (135) |
| | Carbohydrate metabolic process (29) | Exocyst (13) | Metalloendopeptidase activity (126) |
| | Cell redox homeostasis (27) | Cytoplasm (12) | GTPase activity (123) |
| *H. schachtii* | Transmembrane transport (20) | Membrane (56) | Protein binding (267) |
| | Protein secretion (17) | Integral to membrane (50) | ATP binding (118) |
| | Two-component signal transduction system (phosphorelay) (16) | Extracellular space (8) | DNA binding (85) |
| | Biosynthetic process (13) | Cytoplasm (12) | Catalytic activity (85) |
| | Pseudouridine synthesis (10) | Integral to plasma membrane (2) | Transcription factor activity (56) |
| | Protein amino acid glycosylation (10) | Bacterial-type flagellum (2) | Structural constituent of ribosome (54) |
| | Oxidation reduction (9) | Ribosome (1) | Nucleic acid binding (43) |
| | Proteolysis (9) | Nucleus (2) | Oxidoreductase activity (42) |
| | Histidine biosynthetic process (9) | Mitochondrial respiratory chain complex III (3) | RNA binding (33) |
| | Protein targeting (7) | Nucleosome (6) | Hydrolase activity (30) |

Table S19. Highly detected transcription factor genes in the genome of HT and HS when predicted with reference to *H. glycine*

| Domain group | TF family | Description | Pfam ID | TF gene count | | | ratio | |
|---|---|---|---|---|---|---|---|---|
| | | | | HG | HT | HS | HT/HG | HS/HG |
| Zinc finger | zBTB | BTB/POZ domain | PF00651 | 104 | 246 | 117 | 2.37 | 1.13 |
| | C2H2 | Zinc finger, C2H2 type | PF00096 | 109 | 168 | 106 | 1.54 | 0.97 |
| | C3H | Zinc finger C-x8-C-x5-C-x3-H type | PF00642 | 36 | 64 | 26 | 1.78 | 0.72 |
| | C2C2-GATA | GATA zinc finger | PF00320 | 4 | 7 | 4 | 1.75 | 1 |
| Helix-turn-helix | HB | Homeobox domain | PF00046 | 111 | 233 | 99 | 2.1 | 0.89 |
| | MYB | Myb-like DNA-binding domain | PF00249 | 11 | 11 | 8 | 1 | 0.73 |
| | ARID | ARID/BRIGHT DNA binding domain | PF01388 | 5 | 8 | 5 | 1.6 | 1 |
| | E2F-DP | E2F/DP family winged-helix DNA-binding domain | PF02319 | 5 | 7 | 4 | 1.4 | 0.8 |
| | SRF | SRF-type transcription factor (DNA-binding and dimerisation domain) | PF00319 | 2 | 4 | 2 | 2 | 1 |
| | HSF | HSF-type DNA-binding | PF00447 | 1 | 2 | 1 | 2 | 1 |
| Other alpha-helix | HMG | HMG (high mobility group) box | PF00505 | 24 | 36 | 18 | 1.5 | 0.75 |
| | NF-Y | CCAAT-binding transcription factor; NF-YA, NF-YB, NF-YC | PF02045, NF-YB, NF-YC | 11 | 21 | 12 | 1.91 | 1.09 |
| Basic domain | bHLH | Basic helix-loop-helix DNA-binding domain | PF00010 | 23 | 28 | 19 | 1.22 | 0.83 |
| | bZIP | bZIP transcription factor, Basic Leucine zipper domain | PF00170, PF03131 | 19 | 22 | 15 | 1.16 | 0.79 |
| Beta-scaffold factor | CSD | Cold-shock DNA-binding domain | PF00313 | 10 | 14 | 13 | 1.4 | 1.3 |
| Unclassified structure | TUB | Tub family | PF01167 | 3 | 4 | 2 | 1.33 | 0.67 |
| | PC4 | Transcriptional Coactivator p15 (PC4) | PF02229 | 1 | 2 | 1 | 2 | 1 |
| Total | | | | 479 | 877 | 452 | 1.83 | 0.94 |

Table S20. Functional annotation of highly expressed isoform genes in nematode in *H. trifolii* (top 10)

| GO | No. of genes | KEGG | No. of genes | Pfam | No. of genes |
|---|---|---|---|---|---|
| protein binding | 398 | mTOR signaling pathway | 16 | Nematode cuticle collagen N-terminal domain | 74 |
| ATP binding | 277 | PI3K-Akt signaling pathway | 16 | Protein kinase domain | 71 |
| nucleic acid binding | 200 | Human cytomegalovirus infection | 16 | Helicase conserved C-terminal domain | 68 |
| integral to membrane | 140 | Human papillomavirus infection | 16 | RNA recognition motif. (a.k.a. RRM, RBD, or RNP domain) | 65 |
| protein kinase activity | 100 | Human immunodeficiency virus 1 infection | 16 | Collagen triple helix repeat (20 copies) | 65 |
| protein amino acid phosphorylation | 100 | Thermogenesis | 13 | DEAD/DEAH box helicase | 42 |
| proteolysis | 74 | Relaxin signaling pathway | 13 | Ankyrin repeats (3 copies) | 37 |
| structural constituent of cuticle | 74 | Lysine degradation | 13 | Fibronectin type III domain | 28 |
| DNA binding | 73 | Glutathione metabolism | 12 | SPRY domain | 25 |
| regulation of transcription, DNA-dependent | 72 | Purine metabolism | 11 | Immunoglobulin I-set domain | 24 |

Table S21. Functional annotation of highly expressed isoform genes in nematode in *H. schachtii* (top 10)

| GO | No. of genes | KEGG | No. of genes | Pfam | No. of genes |
|---|---|---|---|---|---|
| protein binding | 283 | mTOR signaling pathway | 9 | Protein kinase domain | 56 |
| ATP binding | 186 | PI3K-Akt signaling pathway | 9 | RNA recognition motif. (a.k.a. RRM, RBD, or RNP domain) | 42 |
| nucleic acid binding | 130 | Thermogenesis | 9 | Helicase conserved C-terminal domain | 38 |
| integral to membrane | 100 | Relaxin signaling pathway | 9 | Nematode cuticle collagen N-terminal domain | 25 |
| protein kinase activity | 75 | Human cytomegalovirus infection | 9 | DEAD/DEAH box helicase | 23 |

| | | | | | |
|---|---|---|---|---|---|
| protein amino acid phosphorylation | 75 | Human papillomavirus infection | 9 | Ankyrin repeats (3 copies) | 22 |
| membrane | 52 | Human immunodeficiency virus 1 infection | 9 | SPRY domain | 20 |
| DNA binding | 50 | Glycolysis;Gluconeogenesis | 7 | Collagen triple helix repeat (20 copies) | 19 |
| regulation of transcription, DNA-dependent | 47 | Phosphatidylinositol signaling system | 6 | WD domain, G-beta repeat | 19 |
| zinc ion binding | 44 | Glycerolipid metabolism | 6 | PDZ domain | 18 |

Table S22. Differentially expressed genes (DEG) exhibiting stage-specific high up/downregulation ($log_2$change >10) in *H. trifolii*

| | Highly Upregulated (log2 change >10) | | Highly Downregulated (log2 change >10) | |
|---|---|---|---|---|
| | Protein description | GO description | Protein description | GO description |
| J2 (compared to egg stage) | Phosphatidylinositol 4-phosphate 5-kinase type-1 alpha-like (3)<br>Tartrate-resistant acid phosphatase type 5 isoform X2 (3)<br>Endoglucanase (1)<br>Tartrate-resistant acid phosphatase type 5 precursor (1)<br>Electron transfer flavoprotein subunit alpha, mitochondrial (1)<br>Dimethylaniline monooxygenase (1)<br>Uncharacterized protein LOC106587709, partial (1)<br>Uncharacterized protein LOC112587105 (1)<br>Annexin A11 (1)<br>Tartrate-resistant acid phosphatase type 5 (1)<br>DNA-directed RNA polymerase II subunit RPB1-like, partial (1)<br>Serine/threonine-protein kinase Nek9 | BP:carbohydrate metabolic process; methylation; oxidation reduction; phosphatidylinositol metabolic process; positive regulation of transcription DNA-dependent; protein amino acid phosphorylation<br><br>CC:nucleus<br><br>MF:ATP binding; calcium ion binding; calcium-dependent phospholipid binding; DNA binding; FAD binding; flavin- | Cuticle collagen 6-like (8)<br>Collagen alpha-2(IV) chain-like, partial (6)<br>Collagen alpha-1(III) chain (5)<br>Collagen alpha-1(XI) chain-like (3)<br>Collagen alpha-1(XI) chain isoform X2 (3)<br>Collagen alpha-1(XVII) chain-like (2)<br>Collagen alpha-1(XVII) chain isoform X4 (2)<br>Collagen alpha-1(V) chain isoform X1 (1)<br>BTB/POZ domain-containing protein 6-B-like (1)<br>Collagen alpha-1(I) chain (1)<br>Ubiquitin-like modifier-activating enzyme 1 (1)<br>Peptidyl-prolyl cis-trans isomerase FKBP9 isoform X2 (1) | BP:ion transport; methylation; oxidation reduction; proteolysis<br><br>CC:integral to membrane; membrane; mitochondrion<br><br>MF:carboxypeptidase activity; DNA binding; extracellular ligand-gated ion channel activity; metallocarboxypeptidase activity; metalloendopeptidase activity; methyltransferase activity; |

| | Highly Upregulated (log2 change >10) | | Highly Downregulated (log2 change >10) | |
|---|---|---|---|---|
| | Protein description | GO description | Protein description | GO description |
| | isoform X1 (1)<br>Leucine carboxyl methyltransferase 1 isoform X2 (1)<br>F-box/LRR-repeat protein 7 (1)<br>ADP-dependent glucokinase, partial (1)<br>Nicotinate phosphoribosyltransferase-like (1)<br>Ubiquitin-associated and SH3 domain-containing protein A, partial (1)<br>Homeobox containing 1 L homeolog isoform X4 (1)<br>UDP-glucuronosyltransferase 2A1-like isoform X2 (1)<br>Mucin-17-like (1)<br>BTB/POZ domain-containing protein 6 (1) | containing monooxygenase activity; hydrolase activity; hydrolase activity hydrolyzing O-glycosyl compounds; methyltransferase activity; NADP or NADPH binding; phosphatidylinositol phosphate kinase activity; protein binding; protein kinase activity; transferase activity transferring hexosyl groups | Chymotrypsinogen 2 isoform X2 (1)<br>Sulfide:quinone oxidoreductase, mitochondrial (1)<br>Leucine carboxyl methyltransferase 1 isoform X2 (1)<br>Cytochrome c oxidase assembly factor 6 homolog (1)<br>Mitotic spindle assembly checkpoint protein MAD2A (1)<br>Homeobox protein Hox-A7 (1)<br>Collagen alpha-1(VI) chain (1)<br>Conserved oligomeric Golgi complex subunit 4 (1)<br>Ankyrin repeat and MYND domain-containing protein 2 (1)<br>Zinc metalloproteinase nas-14-like (1)<br>Ran-binding protein 10-like isoform X2 (1)<br>Collagen alpha-1(XI) chain isoform X1 (1)<br>Collagen alpha-1(II) chain isoform X1 (1)<br>Carboxypeptidase B (1)<br>Uncharacterized protein LOC112587105 (1)<br>Collagen alpha-1(XI) chain isoform X3 (1)<br>Aspartyl protease inhibitor (1)<br>Collagen alpha-1(IX) chain-like (1) | oxidoreductase activity; peptidyl-prolyl cis-trans isomerase activity; protein binding; serine-type endopeptidase activity; small protein activating enzyme activity; structural constituent of cuticle; zinc ion binding |

| | Highly Upregulated (log2 change >10) | | Highly Downregulated (log2 change >10) | |
|---|---|---|---|---|
| | Protein description | GO description | Protein description | GO description |
| Female (compared to J2 stage) | Collagen alpha-1(IV) chain-like (14)<br>Cuticle collagen 6-like (13)<br>Collagen alpha-2(IV) chain-like, partial (6)<br>Cathepsin D (4)<br>Collagen alpha-1(III) chain (3)<br>Collagen alpha-1(XI) chain-like (3)<br>Collagen alpha-1(XI) chain isoform X2 (3)<br>Low quality protein: collagen alpha-3(VI) chain (2)<br>Uncharacterized protein LOC100176471 (2)<br>Macrophage mannose receptor 1-like (2)<br>Mucin-2-like (2)<br>Collagen alpha-1(XVII) chain isoform X4 (2)<br>Collagen alpha-1(XVII) chain-like (2)<br>Ran-binding protein 10-like isoform X1 (2)<br>Carbonic anhydrase 4-like (2)<br>Transthyretin-like protein 46 (2)<br>Putative cuticle collagen 90, partial (1)<br>NAC-alpha domain-containing protein 1 isoform X6 (1)<br>Uncharacterized protein LOC112587105 (1) | BP:cell wall macromolecule catabolic process; chitin metabolic process; oxidation reduction; peptidoglycan catabolic process; proteolysis; transmembrane transport<br><br>CC:extracellular region; extracellular space; membrane; mitochondrion<br><br>MF:carboxypeptidase activity; chitin binding; D-arabinono-1,4-lactone oxidase activity; FAD binding; lysozyme activity; metallocarboxypeptidase activity; metalloendopeptidase activity; oxidoreductase | Collagen alpha-1(I) chain isoform X2 (1)<br>Tartrate-resistant acid phosphatase type 5 isoform X2 (4)<br>Phosphatidylinositol 4-phosphate 5-kinase type-1 alpha-like (4)<br>BTB/POZ domain-containing protein 6 (2)<br>Papilin-like isoform X4 (2)<br>Serine/threonine-protein kinase Nek9 isoform X1 (2)<br>Uncharacterized protein LOC109462549 (2)<br>Hypothetical protein BRAFLDRAFT_126894 (2)<br>Secreted chorismate mutase (2)<br>Probable pectate lyase D (2)<br>Low quality protein: putative UDP-Glucuronosyltransferase ugt-48 (1)<br>E3 ubiquitin-protein ligase RNF13 isoform X4 (1)<br>Disintegrin and metalloproteinase domain-containing protein 12 (1)<br>Mucin-17-like (1)<br>Low quality protein: polyadenylate-binding protein 4-like (1)<br>Myophilin-like, partial (1)<br>mRNA export factor (1)<br>UDP-glucuronosyltransferase 2A1-like isoform X2 (1) | BP:cell redox homeostasis; chorismate metabolic process; intracellular protein transport; phosphatidylinositol metabolic process; positive regulation of transcription DNA-dependent; protein amino acid phosphorylation; protein arginylation; proteolysis; trehalose metabolic process<br><br>CC:extracellular region; extracellular space; nucleus<br><br>MF:alpha,alpha-trehalase activity; arginyltransferase activity; ATP binding; calcium ion binding; calcium-dependent phospholipid binding; DNA binding; |

| | Highly Upregulated (log2 change >10) | | Highly Downregulated (log2 change >10) | |
|---|---|---|---|---|
| | Protein description | GO description | Protein description | GO description |
| | Low quality protein: cathepsin D-like (1)<br>mRNA turnover protein 4 homolog (1)<br>Hypothetical protein BRAFLDRAFT_93138 (1)<br>NAC-alpha domain-containing protein 1 isoform X5 (1)<br>Aspartic protease 6-like (1)<br>Collagen alpha-1(I) chain isoform X2 (1)<br>Aspartyl protease inhibitor (1)<br>Collagen alpha-1(IX) chain-like (1)<br>Collagen alpha-1(XI) chain isoform X3 (1)<br>Proteoglycan 4-like (1)<br>Uncharacterized protein LOC106602143 (1)<br>L-gulonolactone oxidase-like (1)<br>Neurofilament heavy polypeptide (1)<br>Carboxypeptidase B (1)<br>Dimethyladenosine transferase 1, mitochondrial (1)<br>Collagen alpha-1(II) chain isoform X1 (1)<br>Lysozyme M1 (1)<br>Collagen alpha-1(XI) chain isoform X1 (1)<br>Mitotic spindle assembly checkpoint protein MAD2A (1) | activity; peptidyl-prolyl cis-trans isomerase activity; protein binding; serine-type endopeptidase inhibitor activity; small protein activating enzyme activity; structural constituent of cuticle; transcription corepressor activity; zinc ion binding | Protein FAM63A isoform X3 (1)<br>Neuronal cell adhesion molecule isoform X6 (1)<br>Homeobox containing 1 L homeolog isoform X4 (1)<br>Arginyl-tRNA--protein transferase 1 isoform X2 (1)<br>Ubiquitin-associated and SH3 domain-containing protein A, partial (1)<br>Failed axon connections (1)<br>Polyubiquitin-like (1)<br>Neuron-specific calcium-binding protein hippocalcin (1)<br>Nicotinate phosphoribosyltransferase-like (1)<br>Dual specificity protein kinase TTK-like (1)<br>Phosphatidylinositol 4-phosphate 5-kinase type-1 beta-like, partial (1)<br>Polyubiquitin-C isoform X8 (1)<br>Tartrate-resistant acid phosphatase type 5 (1)<br>DNA-directed RNA polymerase II subunit RPB1-like, partial (1)<br>General transcription factor 3C polypeptide 1 (1)<br>Peptide-N(4)-(N-acetyl-beta-glucosaminyl)asparagine amidase (1)<br>Opioid growth factor receptor-like | glucosidase activity; GTP binding; GTPase activity; hydrolase activity; metalloendopeptidase activity; nucleic acid binding; pectate lyase activity; phosphatidylinositol phosphate kinase activity; protein binding; protein kinase activity; serine-type endopeptidase inhibitor activity; transferase activity transferring hexosyl groups |

| | Highly Upregulated (log2 change >10) | | Highly Downregulated (log2 change >10) | |
|---|---|---|---|---|
| | Protein description | GO description | Protein description | GO description |
| | Filaggrin-2-like (1)<br>Serine/arginine repetitive matrix protein 1-like (1)<br>Cytochrome c oxidase assembly factor 6 homolog (1)<br>Uncharacterized protein LOC112243670 (1)<br>Beta-porphyranase A (1)<br>Vitellogenin-1 (1)<br>Heat shock factor-binding protein 1-like (1)<br>Ubiquitin-like modifier-activating enzyme 1 (1)<br>Ran-binding protein 10-like isoform X2 (1)<br>Low quality protein: uncharacterized transporter slc-17.2-like (1)<br>Ribosome-binding protein 1-like (1)<br>Peptidyl-prolyl cis-trans isomerase FKBP9 isoform X2 (1)<br>Zinc metalloproteinase nas-14-like (1)<br>Uncharacterized protein C1259.12c (1)<br>Sulfide:quinone oxidoreductase, mitochondrial (1)<br>Collagen alpha-1(VI) chain (1)<br>Reticulocyte-binding protein 2 homolog a (1)<br>Neurofilament heavy polypeptide (1) | | isoform X6 (1)<br>Annexin A11 (1)<br>OV-16 antigen-like, partial (1)<br>NF-kappa-B inhibitor-interacting Ras-like protein 2 (1)<br>Regulator of nonsense transcripts 3B-like (1)<br>Uncharacterized protein LOC111192142 (1)<br>Low quality protein: hornerin (1)<br>Transthyretin-like protein 46 (1)<br>26S protease regulatory subunit 10B (1)<br>Probable ATP-dependent RNA helicase DDX4 isoform X2 (1)<br>Uncharacterized protein LOC106587709, partial (1)<br>Trehalase-like (1)<br>Electron transfer flavoprotein subunit alpha, mitochondrial (1)<br>Tartrate-resistant acid phosphatase type 5 isoform X3 (1)<br>Sperm-specific antigen 2 (1)<br>Thaumatin-like protein 1 (1)<br>GTP-binding protein SAR1b-like (1)<br>Probable arabinogalactan endo-beta-1,4-galactanase A (1) | |
| Female | Collagen alpha-1(IV) chain-like (10) | BP:chitin metabolic | 26S protease regulatory subunit 10B | BP:cell redox |

| | Highly Upregulated (log2 change >10) | | Highly Downregulated (log2 change >10) | |
|---|---|---|---|---|
| | Protein description | GO description | Protein description | GO description |
| (compared to egg stage) | Cuticle collagen 6-like (3)<br>Ran-binding protein 10-like isoform X1 (2)<br>Low quality protein: collagen alpha-3(VI) chain (2)<br>Uncharacterized protein LOC100176471 (2)<br>Mucin-2-like (2)<br>Transthyretin-like protein 46 (2)<br>Low quality protein: uncharacterized transporter slc-17.2-like (2)<br>Putative cuticle collagen 90, partial (1)<br>NAC-alpha domain-containing protein 1 isoform X6 (1)<br>Macrophage mannose receptor 1-like (1)<br>Uncharacterized protein LOC112587105 (1)<br>Low quality protein: cathepsin D-like (1)<br>mRNA turnover protein 4 homolog (1)<br>Collagen alpha-5(IV) chain-like (1)<br>NAC-alpha domain-containing protein 1 isoform X5 (1)<br>Collagen alpha-5(IV) chain (1)<br>Proteoglycan 4-like (1)<br>Filaggrin-2-like (1)<br>Aspartic protease 6-like (1)<br>WAP four-disulfide core domain protein | process; oxidation reduction; proteolysis; transmembrane transport<br><br>CC:extracellular region; extracellular space ; membrane<br><br>MF:calcium ion binding; chitin binding; D-arabinono-1, 4-lactone oxidase activity; DNA binding; FAD binding; oxidoreductase activity; protein binding; serine-type endopeptidase activity; serine-type endopeptidase inhibitor activity; structural constituent of cuticle; transcription corepressor activity; transferase activity transferring hexosyl groups | (1)<br>Ankyrin repeat and MYND domain-containing protein 2 (1)<br>Arginyl-tRNA--protein transferase 1 isoform X2 (1)<br>Collagen alpha-1(III) chain (1)<br>Conserved oligomeric Golgi complex subunit 4 (1)<br>Disintegrin and metalloproteinase domain-containing protein 12 (1)<br>GTP-binding protein SAR1b-like (1)<br>Leucine carboxyl methyltransferase 1 isoform X2 (1)<br>Myelin regulatory factor isoform X3 (1)<br>NF-kappa-B inhibitor-interacting Ras-like protein 2 (1)<br>Peptide-N(4)-(N-acetyl-beta-glucosaminyl)asparagine amidase (1)<br>Probable ATP-dependent RNA helicase DDX4 isoform X2 (1)<br>Regulator of nonsense transcripts 3B-like (1)<br>Transthyretin-like protein 46 (1) | homeostasis; intracellular protein transport; methylation; protein arginylation; proteolysis<br><br>CC:extracellular space<br><br>MF:arginyltransferase activity; ATP binding; DNA binding; GTP binding; GTPase activity; metalloendopeptidase activity; methyltransferase activity; nucleic acid binding; protein binding; structural constituent of cuticle |

| Highly Upregulated (log2 change >10) | | Highly Downregulated (log2 change >10) | |
|---|---|---|---|
| Protein description | GO description | Protein description | GO description |
| 6A-like (1) | | | |
| Cuticle collagen 39-like (1) | | | |
| L-gulonolactone oxidase-like (1) | | | |
| Neurofilament heavy polypeptide (1) | | | |
| Dimethyladenosine transferase 1, mitochondrial (1) | | | |
| Low quality protein: putative UDP-glucuronosyltransferase ugt-48 (1) | | | |
| Low quality protein: protein kinase C and casein kinase Substrate in neurons protein 1-like (1) | | | |
| Epidermal growth factor receptor substrate 15-like 1 isoform (1) | | | |
| Chymotrypsinogen B-like isoform X1 (1) | | | |
| Serine/arginine repetitive matrix protein 1-like (1) | | | |
| Uncharacterized protein LOC112243670 (1) | | | |
| Heat shock factor-binding protein 1-like (1) | | | |
| Ribosome-binding protein 1-like (1) | | | |
| Methyltransferase-like protein 13 (1) | | | |
| Myelin regulatory factor isoform X3 (1) | | | |
| Beta-porphyranase A (1) | | | |
| Cysteine synthase, chloroplastic/chromoplastic-like (1) | | | |
| Low quality protein: F-box only protein 38-like (1) | | | |

Table S23. Differentially expressed genes (DEG) exhibiting stage-specific high up/downregulation ($log_2$change >10) in *H. schachtii*

| | Highly Upregulated (log2 change >10) | | Highly Downregulated (log2 change >10) | |
|---|---|---|---|---|
| | Protein description | GO description | Protein description | GO description |
| J2 (compared to egg stage) | Endoglucanase (3)<br>Olfactory receptor 142-like (2)<br>Papain inhibitor (2)<br>Probable pectate lyase D (2)<br>Carbonic anhydrase 4-like (1)<br>Cell wall protein IFF6-like (1)<br>Circumsporozoite protein (1)<br>Cysteine-rich secretory protein 2-like (1)<br>Cytochrome P450 3A9-like (1)<br>Dynein light chain LC6, flagellar outer arm isoform X2 (1)<br>Low quality protein: hornerin (1)<br>Mucin-17-like (1)<br>Neurofilament medium polypeptide (1)<br>Papilin-like isoform X4 (1)<br>Phosphatidylinositol 4-phosphate 5-kinase type-1 gamma (1)<br>Phosphatidylinositol 4-phosphate 5-kinase type-1 gamma-like (1)<br>Probable arabinogalactan endo-beta-1,4-galactanase A (1)<br>Secreted chorismate mutase (1)<br>Uncharacterized protein LOC106587709, partial (1) | BP:carbohydrate metabolic process; chorismate metabolic process; microtubule-based process; oxidation reduction; phosphatidylinositol metabolic process; signal transduction<br>CC:dynein complex; extracellular region; membrane<br>MF:carbohydrate binding; glucosidase activity; heme binding; hydrolase activity hydrolyzing O-glycosyl compounds; iron ion binding; oxidoreductase activity acting on paired donors with incorporation or reduction of molecular oxygen; pectate lyase activity; phosphatidylinositol phosphate kinase activity | Uncharacterized protein C1259.12c (2)<br>E3 ubiquitin-protein ligase pellino homolog 1 (1)<br>Ran-binding protein 10 isoform X2 (1)<br>Protein DDI1 homolog 2-like isoform X2 (1)<br>Transcription factor SOX-5 isoform X8 (1)<br>Protein DDI1 homolog 2 isoform X1 (1)<br>M protein, serotype 24-like isoform X1 (1)<br>DNA damage-inducible protein 1 (1)<br>Uncharacterized protein LOC109476912 (1)<br>Peroxisomal acyl-coenzyme A oxidase 1-like (1)<br>Transcription factor AP-2-epsilon (1)<br>Carboxypeptidase B-like (1)<br>Ankyrin repeat domain-containing protein 13C-like (1)<br>Hypothetical protein BRAFLDRAFT_241003 (1)<br>BTB/POZ domain-containing protein 6-B-like (1) | BP:fatty acid beta-oxidation; oxidation reduction; protein polyubiquitination; proteolysis; regulation of Toll signaling pathway<br>CC:peroxisome<br>MF:acyl-CoA oxidase activity; aspartic-type endopeptidase activity; calcium-dependent cysteine-type endopeptidase activity; carboxypeptidase activity; metallocarboxypeptidase activity; oxidoreductase activity acting on the CH-CH group of donors; protein binding; ubiquitin protein ligase activity; zinc ion binding |

| | Highly Upregulated (log2 change >10) | | Highly Downregulated (log2 change >10) | |
|---|---|---|---|---|
| | Protein description | GO description | Protein description | GO description |
| | | ; protein binding; serine-type endopeptidase inhibitor activity | | |
| Female (compared to J2 stage) | Collagen alpha-1(IV) chain-like (6)<br>Beta-porphyranase A (2)<br>Cuticle collagen 6-like (2)<br>Cuticle collagen 10 (2)<br>Transcription factor AP-2-epsilon (2)<br>Serine/arginine repetitive matrix protein 5-like (1)<br>Hydroxyacyl-coenzyme A dehydrogenase, mitochondrial (1)<br>Transthyretin-like protein 46 (1)<br>Cathepsin D (1)<br>Uncharacterized protein LOC106602143 (1)<br>Flocculation protein FLO11 (1)<br>Neurofilament heavy polypeptide (1)<br>Carbonic anhydrase 4-like (1)<br>E3 ubiquitin-protein ligase pellino homolog 1 (1)<br>Ran-binding protein 10-like isoform X1 (1)<br>Embryonic pepsinogen-like (1)<br>Uncharacterized protein LOC112243670 (1)<br>Ran-binding protein 10 isoform X2 (1) | BP:chitin metabolic process; fatty acid beta-oxidation; fatty acid metabolic process; lipid transport<br>; oxidation reduction; protein polyubiquitination; proteolysis; regulation of Toll signaling pathway<br>; transmembrane transport<br><br>CC:extracellular region; extracellular space; peroxisome<br><br>MF:3-hydroxyacyl-CoA dehydrogenase activity; acyl-CoA oxidase activity; aspartic-type endopeptidase activity; calcium-dependent cysteine-type endopeptidase activity; chitin binding; lipid | Endoglucanase (4)<br>Low quality protein: putative UDP-Glucuronosyltransferase ugt-48 (2)<br>Probable pectate lyase D (2)<br>Papain inhibitor (2)<br>Neurofilament medium polypeptide (2)<br>Olfactory receptor 142-like (1)<br>Phosphatidylinositol 4-phosphate 5-kinase type-1 gamma-like (1)<br>Papilin-like isoform X4 (1)<br>Cell wall protein IFF6-like (1)<br>Phosphatidylinositol 4-phosphate 5-kinase type-1 gamma (1)<br>Carbonic anhydrase 4-like (1)<br>Mucin-17-like (1)<br>Mucin-2-like (1)<br>Phosphatidylinositol 4-phosphate 5-kinase type-1 alpha-like Isoform X1 (1)<br>Neuronal acetylcholine receptor subunit beta-4 (1)<br>Cytochrome P450 3A9-like (1)<br>Probable arabinogalactan endo-beta-1,4-galactanase A (1)<br>Titin homolog (1)<br>Poly(A) polymerase gamma, partial | BP:carbohydrate metabolic process; ion transport; oxidation reduction; phosphatidylinositol metabolic process; RNA polyadenylation; signal transduction<br><br>CC:extracellular region; integral to membrane; membrane<br><br>MF:carbohydrate binding; extracellular ligand-gated ion channel activity; glucosidase activity; heme binding; hydrolase activity hydrolyzing O-glycosyl compounds; iron ion binding; oxidoreductase activity acting on paired donors with incorporation or reduction of molecular oxygen; pectate lyase |

| | Highly Upregulated (log2 change >10) | | Highly Downregulated (log2 change >10) | |
|---|---|---|---|---|
| | Protein description | GO description | Protein description | GO description |
| | Putative tyrosinase-like protein tyr-1 (1)<br>Aspartic protease 6-like (1)<br>Protein DDI1 homolog 2-like isoform X2 (1)<br>Ovochymase-1 (1)<br>Phospholipase A2-like protein Y52B11A.8 (1)<br>Transcription factor SOX-5 isoform X8 (1)<br>Uncharacterized protein LOC100176471 (1)<br>Peroxisomal acyl-coenzyme A oxidase 1-like (1)<br>Uncharacterized protein LOC109476912 (1)<br>Blastula protease 10-like (1)<br>Low quality protein: uncharacterized transporter slc-17.2-like (1)<br>Macrophage mannose receptor 1-like (1)<br>Vitellogenin-6-like (1) | transporter activity ; metalloendopeptidase activity; oxidoreductase activity; oxidoreductase activity acting on the CH-CH group of donors; protein binding; serine-type endopeptidase activity; structural constituent of cuticle; ubiquitin protein ligase activity | (1) | activity; phosphatidylinositol phosphate kinase activity; polynucleotide adenylyltransferase activity; serine-type endopeptidase inhibitor activity; transferase activity transferring hexosyl groups |
| Female (compared to egg stage) | Collagen alpha-1(IV) chain-like (4)<br>Cuticle collagen 6-like (2)<br>Cuticle collagen 10 (1)<br>Embryonic pepsinogen-like (1)<br>Macrophage mannose receptor 1-like (1)<br>Octapeptide-repeat protein T2-like, partial (1) | BP:chitin metabolic process; lipid transport; proteolysis<br>CC:extracellular region;<br>MF: chitin binding; lipid transporter activity; oxidoreductase activity; protein binding; serine- | 26S proteasome non-ATPase regulatory subunit 11, Hypothetical protein BRAFLDRAFT_241003 | nohit |

| | Highly Upregulated (log2 change >10) | | Highly Downregulated (log2 change >10) | |
|---|---|---|---|---|
| | Protein description | GO description | Protein description | GO description |
| | Olfactory receptor 142-like (1)<br>Putative tyrosinase-like protein tyr-1 (1)<br>Serine protease 42 (1)<br>Vitellogenin-6-like (1) | type endopeptidase activity; structural constituent of cuticle | | |

Table S24. BLAST analysis of the genes present in the mitochondrial genome of *H. trifolii*

| Protein | | | |
|---|---|---|---|
| Locus Tag | NCBI match | Description | Species |
| HT_MTp001 | YP_009110111.1 | NADH dehydrogenase subunit 1 (mitochondrion) | *Caenorhabditis tropicalis* |
| HT_MTp002 | YP_009186371.1 | ATP synthase F0 subunit 6 (mitochondrion) | *Strongyloides ratti* |
| HT_MTp003 | YP_009176853.1 | NADH dehydrogenase subunit 4L (mitochondrion) | *Strongyloides venezuelensis* |
| HT_MTp004 | YP_009240276.1 | NADH dehydrogenase subunit 5 (mitochondrion) | *Haemonchus placei* |
| HT_MTp005 | YP_009312523.1 | Cytochrome c oxidase subunit II (mitochondrion) | *Pseudoterranova bulbosa* |
| HT_MTp006 | YP_001936466.1 | NADH dehydrogenase subunit 3 (mitochondrion) | *Toxocara cati* |
| HT_MTp007 | YP_009229276.1 | Cytochrome c oxidase subunit I (mitochondrion) | *Koerneria sudhausi* |
| HT_MTp008 | YP_009176855.1 | NADH dehydrogenase subunit 2 (mitochondrion) | *Strongyloides venezuelensis* |
| HT_MTp009 | YP_009243337.1 | Cytochrome c oxidase subunit III (mitochondrion) | *Heterakis beramporia* |
| HT_MTp010 | YP_009312557.1 | NADH dehydrogenase subunit 4 (mitochondrion) | *Pseudoterranova krabbei* |
| HT_MTp011 | YP_009059437.1 | Cytochrome b (mitochondrion) | *Philometroides sanguineus* |
| HT_MTp012 | YP_003434044.1 | NADH dehydrogenase subunit 6 (mitochondrion) | *Trichostrongylus axei* |
| tRNA & rRNA | | | |
| Locus Tag | RNACentral match | Description | Species |
| HT_MTt001 | URS00001E547C_51029 | tRNA-Asn | *Heterodera glycines* (soybean cyst nematode) |
| HT_MTt002 | URS000043AD9E_334426 | tRNA-Thr | *Angiostrongylus costaricensis* |
| HT_MTr001 | URS0000503835_51029 | 16S ribosomal RNA | *Heterodera glycines* (soybean cyst nematode) |
| HT_MTr002 | URS0000333578_51029 | 12S ribosomal RNA | *Heterodera glycines* (soybean cyst nematode) |

Table S25. BLAST analysis of the genes present in the mitochondrial genome of *H. schachtii*

| Protein | | | |
|---|---|---|---|
| Locus Tag | NCBI match | Description | Species |
| HS_MTp001 | YP_817451.1 | NADH dehydrogenase subunit 1 (mitochondrion) | *Heterorhabditis bacteriophora* |

| | | | |
|---|---|---|---|
| HS_MTp002 | YP_009346420.1 | ATP synthase F0 subunit 6 (mitochondrion) | *Longidorus vineacola* |
| HS_MTp003 | YP_007183175.1 | NADH dehydrogenase subunit 5 (mitochondrion) | *Dictyocaulus eckerti* |
| HS_MTp004 | YP_009312523.1 | Cytochrome c oxidase subunit II (mitochondrion) | *Pseudoterranova bulbosa* |
| HS_MTp005 | YP_001700363.2 | NADH dehydrogenase subunit 3 (mitochondrion) | *Haemonchus contortus* |
| HS_MTp006 | YP_009391025.1 | Cytochrome c oxidase subunit I (mitochondrion) | *Caenorhabditis doughertyi* |
| HS_MTp007 | YP_009176855.1 | NADH dehydrogenase subunit 2 (mitochondrion) | *Strongyloides venezuelensis* |
| HS_MTp008 | YP_537094.1 | Cytochrome c oxidase subunit III (mitochondrion) | *Anisakis simplex* |
| HS_MTp009 | YP_009312557.1 | NADH dehydrogenase subunit 4 (mitochondrion) | *Pseudoterranova krabbei* |
| HS_MTp010 | YP_009059437.1 | Cytochrome b (mitochondrion) | *Philometroides sanguineus* |
| HS_MTp011 | YP_009113346.1 | NADH dehydrogenase subunit 6 (mitochondrion) | *Anisakis berlandi* |

tRNA & rRNA

| Locus Tag | RNACentral match | Description | Species |
|---|---|---|---|
| HS_MTt001 | URS00002B9F8E_51029 | tRNA-Met | *Heterodera glycines* (soybean cyst nematode) |
| HS_MTt002 | URS00003401C2_6313 | tRNA-Thr | *Angiostrongylus cantonensis* (rat lungworm) |
| HS_MTt003 | URS0000488DA8_51029 | tRNA-Phe | *Heterodera glycines* (soybean cyst nematode) |
| HS_MTt004 | URS0000DEF05E_1517492 | tRNA-Pro | *Globodera ellingtonae* |
| HS_MTr001 | URS0000333578_51029 | 12S ribosomal RNA | *Heterodera glycines* (soybean cyst nematode) |
| HS_MTr002 | URS0000503835_51029 | 16S ribosomal RNA | *Heterodera glycines* (soybean cyst nematode) |

Table S26. Comparison of Ka/Ks rations of *H. trifolii* and neighboring nematode mitochondrial protein coding genes (PCGs)

| **PCG** | **Species 1** | **Species 2** | **Method** | **Ka** | **Ks** | **Ka/Ks** | **p value(Fisher)** |
|---|---|---|---|---|---|---|---|
| ATP6 | *H.schachtii* | *Caenorhabditis-sp* | GMYN | 0.67 | 2.82 | 0.24 | 2.82697E-08 |
| ATP6 | *H.schachtii* | *H.glycines* | GMYN | 0.10 | 1.13 | 0.09 | 2.81063E-19 |
| ATP6 | *H.schachtii* | *H.trifolii* | GMYN | 0.07 | 1.34 | 0.05 | 1.24287E-19 |
| ATP6 | *H.schachtii* | *K.sudhausi* | GMYN | 0.68 | 2.91 | 0.23 | 2.07481E-07 |
| ATP6 | *H.schachtii* | *O.columbianum* | GMYN | 0.67 | 2.92 | 0.23 | 1.36986E-09 |

| | | | | | | | |
|---|---|---|---|---|---|---|---|
| ATP6 | *H.schachtii* | *S.stercoralis* | GMYN | 0.54 | 2.60 | 0.21 | 6.2964E-07 |
| ATP6 | *H.schachtii* | *U.sanguinis-haplo45* | GMYN | 0.62 | 2.08 | 0.30 | 1.41706E-05 |
| ATP6 | *H.trifolii* | *Caenorhabditis-sp* | GMYN | 0.68 | 2.61 | 0.26 | 0 |
| ATP6 | *H.trifolii* | *H.glycines* | GMYN | 0.12 | 2.65 | 0.04 | 0 |
| ATP6 | *H.trifolii* | *K.sudhausi* | GMYN | 0.74 | 2.77 | 0.27 | 0 |
| ATP6 | *H.trifolii* | *Litoditis-aff.marina-Pml* | GMYN | 0.67 | 2.67 | 0.25 | 0 |
| ATP6 | *H.trifolii* | *O.columbianum* | GMYN | 0.71 | 2.78 | 0.26 | 3.19175E-12 |
| ATP6 | *H.trifolii* | *S.stercoralis* | GMYN | 0.55 | 2.43 | 0.23 | 8.09934E-05 |
| ATP6 | *H.trifolii* | *U.sanguinis-haplo45* | GMYN | 0.64 | 2.91 | 0.22 | 2.5845E-06 |
| ATP6 | *H.glycines* | *Caenorhabditis-sp* | GMYN | 0.66 | 2.76 | 0.24 | 1.03249E-09 |
| ATP6 | *H.glycines* | *K.sudhausi* | GMYN | 0.71 | 2.73 | 0.26 | 0 |
| ATP6 | *H.glycines* | *Litoditis-aff.marina-Pml* | GMYN | 0.70 | 2.61 | 0.27 | 0 |
| ATP6 | *H.glycines* | *O.columbianum* | GMYN | 0.72 | 2.85 | 0.25 | 0 |
| ATP6 | *H.glycines* | *S.stercoralis* | GMYN | 0.52 | 4.19 | 0.12 | 2.58614E-06 |
| ATP6 | *H.glycines* | *U.sanguinis-haplo45* | GMYN | 0.70 | 3.00 | 0.23 | 2.2073E-09 |
| ATP6 | *O.columbianum* | *Caenorhabditis-sp* | GMYN | 0.09 | 3.00 | 0.03 | 0 |
| ATP6 | *O.columbianum* | *K.sudhausi* | GMYN | 0.13 | 3.19 | 0.04 | 4.77534E-45 |
| ATP6 | *O.columbianum* | *Litoditis-aff.marina-Pml* | GMYN | 0.08 | 2.99 | 0.03 | 1.85778E-38 |
| ATP6 | *O.columbianum* | *S.stercoralis* | GMYN | 0.30 | 2.78 | 0.11 | 0 |
| ATP6 | *O.columbianum* | *U.sanguinis-haplo45* | GMYN | 0.10 | 2.50 | 0.04 | 9.24161E-30 |
| ATP6 | *K.sudhausi* | *Caenorhabditis-sp* | GMYN | 0.12 | 3.04 | 0.04 | 1.3004E-41 |
| ATP6 | *K.sudhausi* | *S.stercoralis* | GMYN | 0.30 | 2.74 | 0.11 | 0 |
| ATP6 | *K.sudhausi* | *U.sanguinis-haplo45* | GMYN | 0.15 | 3.22 | 0.05 | 1.66944E-30 |
| ATP6 | *Caenorhabditis-sp* | *Litoditis-aff.marina-Pml* | GMYN | 0.03 | 2.82 | 0.01 | 0 |
| ATP6 | *Caenorhabditis-sp* | *S.stercoralis* | GMYN | 0.29 | 2.47 | 0.12 | 0 |
| ATP6 | *Caenorhabditis-sp* | *U.sanguinis-haplo45* | GMYN | 0.11 | 3.14 | 0.04 | 0 |
| ATP6 | *U.sanguinis-haplo45* | *Litoditis-aff.marina-Pml* | GMYN | 0.11 | 3.14 | 0.03 | 1.93593E-40 |
| ATP6 | *U.sanguinis-haplo45* | *S.stercoralis* | GMYN | 0.31 | 2.76 | 0.11 | 0 |
| ATP6 | *Litoditis-aff.marina-Pml* | *S.stercoralis* | GMYN | 0.29 | 2.50 | 0.11 | 0 |
| COX1 | *H.schachtii* | *Caenorhabditis-sp* | GMYN | 0.29 | 3.62 | 0.08 | 0 |
| COX1 | *H.schachtii* | *G.ellingtonae-chr1* | GMYN | 0.16 | 4.02 | 0.04 | 0 |
| COX1 | *H.schachtii* | *H.cardiolata* | GMYN | 0.13 | 3.48 | 0.04 | 0 |
| COX1 | *H.schachtii* | *H.glycines* | GMYN | 0.01 | 1.74 | 0.01 | 1.78423E-85 |

| | | | | | | | |
|---|---|---|---|---|---|---|---|
| COX1 | *H.schachtii* | *H.trifolii* | GMYN | 0.02 | 3.84 | 0.00 | 3.1549E-145 |
| COX1 | *H.schachtii* | *K.sudhausi* | GMYN | 0.30 | 3.68 | 0.08 | 0 |
| COX1 | *H.schachtii* | *Litoditis-aff.marina-Pml* | GMYN | 0.30 | 3.59 | 0.08 | 0 |
| COX1 | *H.schachtii* | *O.columbianum* | GMYN | 0.31 | 3.72 | 0.08 | 0 |
| COX1 | *H.schachtii* | *P.chalcoensis* | GMYN | 0.11 | 3.64 | 0.03 | 0 |
| COX1 | *H.schachtii* | *S.stercoralis* | GMYN | 0.27 | 3.49 | 0.08 | 0 |
| COX1 | *H.schachtii* | *U.sanguinis-haplo45* | GMYN | 0.30 | 3.65 | 0.08 | 0 |
| COX1 | *H.trifolii* | *Caenorhabditis-sp* | GMYN | 0.30 | 3.62 | 0.08 | 0 |
| COX1 | *H.trifolii* | *G.ellingtonae-chr1* | GMYN | 0.16 | 4.01 | 0.04 | 0 |
| COX1 | *H.trifolii* | *H.cardiolata* | GMYN | 0.13 | 3.47 | 0.04 | 0 |
| COX1 | *H.trifolii* | *H.glycines* | GMYN | 0.01 | 3.58 | 0.00 | 0 |
| COX1 | *H.trifolii* | *K.sudhausi* | GMYN | 0.31 | 3.69 | 0.08 | 0 |
| COX1 | *H.trifolii* | *Litoditis-aff.marina-Pml* | GMYN | 0.31 | 3.60 | 0.09 | 0 |
| COX1 | *H.trifolii* | *O.columbianum* | GMYN | 0.32 | 3.72 | 0.09 | 0 |
| COX1 | *H.trifolii* | *P.chalcoensis* | GMYN | 0.12 | 3.65 | 0.03 | 0 |
| COX1 | *H.trifolii* | *S.stercoralis* | GMYN | 0.27 | 3.49 | 0.08 | 0 |
| COX1 | *H.trifolii* | *U.sanguinis-haplo45* | GMYN | 0.31 | 3.65 | 0.09 | 0 |
| COX1 | *H.glycines* | *Caenorhabditis-sp* | GMYN | 0.30 | 3.56 | 0.08 | 0 |
| COX1 | *H.glycines* | *G.ellingtonae-chr1* | GMYN | 0.16 | 3.98 | 0.04 | 0 |
| COX1 | *H.glycines* | *H.cardiolata* | GMYN | 0.13 | 3.41 | 0.04 | 0 |
| COX1 | *H.glycines* | *K.sudhausi* | GMYN | 0.31 | 3.65 | 0.08 | 0 |
| COX1 | *H.glycines* | *Litoditis-aff.marina-Pml* | GMYN | 0.30 | 3.54 | 0.09 | 0 |
| COX1 | *H.glycines* | *O.columbianum* | GMYN | 0.31 | 3.67 | 0.08 | 0 |
| COX1 | *H.glycines* | *P.chalcoensis* | GMYN | 0.11 | 3.55 | 0.03 | 0 |
| COX1 | *H.glycines* | *S.stercoralis* | GMYN | 0.27 | 3.42 | 0.08 | 0 |
| COX1 | *H.glycines* | *U.sanguinis-haplo45* | GMYN | 0.31 | 3.59 | 0.09 | 0 |
| COX1 | *H.cardiolata* | *Caenorhabditis-sp* | GMYN | 0.29 | 3.49 | 0.08 | 0 |
| COX1 | *H.cardiolata* | *G.ellingtonae-chr1* | GMYN | 0.16 | 3.94 | 0.04 | 0 |
| COX1 | *H.cardiolata* | *K.sudhausi* | GMYN | 0.30 | 3.59 | 0.08 | 0 |
| COX1 | *H.cardiolata* | *Litoditis-aff.marina-Pml* | GMYN | 0.30 | 3.46 | 0.09 | 0 |
| COX1 | *H.cardiolata* | *O.columbianum* | GMYN | 0.29 | 3.59 | 0.08 | 0 |
| COX1 | *H.cardiolata* | *P.chalcoensis* | GMYN | 0.13 | 3.53 | 0.04 | 0 |
| COX1 | *H.cardiolata* | *S.stercoralis* | GMYN | 0.29 | 3.33 | 0.09 | 0 |
| COX1 | *H.cardiolata* | *U.sanguinis-haplo45* | GMYN | 0.27 | 3.49 | 0.08 | 0 |
| COX1 | *P.chalcoensis* | *Caenorhabditis-sp* | GMYN | 0.28 | 3.58 | 0.08 | 0 |
| COX1 | *P.chalcoensis* | *G.ellingtonae-chr1* | GMYN | 0.14 | 4.05 | 0.03 | 0 |

| | | | | | | | |
|---|---|---|---|---|---|---|---|
| COX1 | *P.chalcoensis* | *K.sudhausi* | GMYN | 0.31 | 3.67 | 0.09 | 0 |
| COX1 | *P.chalcoensis* | *Litoditis-aff.marina-Pml* | GMYN | 0.29 | 3.52 | 0.08 | 0 |
| COX1 | *P.chalcoensis* | *O.columbianum* | GMYN | 0.30 | 3.69 | 0.08 | 0 |
| COX1 | *P.chalcoensis* | *S.stercoralis* | GMYN | 0.28 | 3.45 | 0.08 | 0 |
| COX1 | *P.chalcoensis* | *U.sanguinis-haplo45* | GMYN | 0.28 | 3.60 | 0.08 | 0 |
| COX1 | *O.columbianum* | *Caenorhabditis-sp* | GMYN | 0.05 | 3.85 | 0.01 | 0 |
| COX1 | *O.columbianum* | *G.ellingtonae-chr1* | GMYN | 0.32 | 4.08 | 0.08 | 0 |
| COX1 | *O.columbianum* | *K.sudhausi* | GMYN | 0.06 | 3.93 | 0.01 | 1.1372E-127 |
| COX1 | *O.columbianum* | *Litoditis-aff.marina-Pml* | GMYN | 0.05 | 3.85 | 0.01 | 6.2603E-123 |
| COX1 | *O.columbianum* | *S.stercoralis* | GMYN | 0.13 | 3.65 | 0.04 | 0 |
| COX1 | *O.columbianum* | *U.sanguinis-haplo45* | GMYN | 0.03 | 3.93 | 0.01 | 1.2448E-160 |
| COX1 | *K.sudhausi* | *Caenorhabditis-sp* | GMYN | 0.05 | 3.75 | 0.01 | 0 |
| COX1 | *K.sudhausi* | *G.ellingtonae-chr1* | GMYN | 0.34 | 4.08 | 0.08 | 0 |
| COX1 | *K.sudhausi* | *Litoditis-aff.marina-Pml* | GMYN | 0.05 | 3.70 | 0.01 | 0 |
| COX1 | *K.sudhausi* | *S.stercoralis* | GMYN | 0.13 | 3.65 | 0.04 | 0 |
| COX1 | *K.sudhausi* | *U.sanguinis-haplo45* | GMYN | 0.06 | 3.85 | 0.02 | 0 |
| COX1 | *Caenorhabditis-sp* | *G.ellingtonae-chr1* | GMYN | 0.30 | 4.01 | 0.08 | 0 |
| COX1 | *Caenorhabditis-sp* | *Litoditis-aff.marina-Pml* | GMYN | 0.03 | 3.68 | 0.01 | 0 |
| COX1 | *Caenorhabditis-sp* | *S.stercoralis* | GMYN | 0.13 | 3.54 | 0.04 | 0 |
| COX1 | *Caenorhabditis-sp* | *U.sanguinis-haplo45* | GMYN | 0.05 | 3.82 | 0.01 | 0 |
| COX1 | *U.sanguinis-haplo45* | *G.ellingtonae-chr1* | GMYN | 0.32 | 4.02 | 0.08 | 0 |
| COX1 | *U.sanguinis-haplo45* | *Litoditis-aff.marina-Pml* | GMYN | 0.05 | 3.74 | 0.01 | 5.7149E-131 |
| COX1 | *U.sanguinis-haplo45* | *S.stercoralis* | GMYN | 0.13 | 3.59 | 0.04 | 0 |
| COX1 | *Litoditis-aff.marina-Pml* | *G.ellingtonae-chr1* | GMYN | 0.32 | 4.02 | 0.08 | 0 |
| COX1 | *Litoditis-aff.marina-Pml* | *S.stercoralis* | GMYN | 0.13 | 3.53 | 0.04 | 0 |
| COX1 | *G.ellingtonae-chr1* | *S.stercoralis* | GMYN | 0.32 | 3.89 | 0.08 | 0 |
| COX2 | *H.schachtii* | *Caenorhabditis-sp* | GMYN | 0.41 | 2.96 | 0.14 | 0 |
| COX2 | *H.schachtii* | *G.ellingtonae-chr1* | GMYN | 0.23 | 3.38 | 0.07 | 0 |
| COX2 | *H.schachtii* | *H.cardiolata* | GMYN | 0.12 | 2.40 | 0.05 | 0 |
| COX2 | *H.schachtii* | *H.glycines* | GMYN | 0.01 | 1.14 | 0.01 | 9.82732E-37 |
| COX2 | *H.schachtii* | *H.trifolii* | GMYN | 0.03 | 3.07 | 0.01 | 2.44423E-59 |
| COX2 | *H.schachtii* | *K.sudhausi* | GMYN | 0.39 | 3.14 | 0.12 | 3.129E-27 |

| | | | | | | | |
|---|---|---|---|---|---|---|---|
| COX2 | *H.schachtii* | *Litoditis-aff.marina-Pml* | GMYN | 0.38 | 2.98 | 0.13 | 0 |
| COX2 | *H.schachtii* | *O.columbianum* | GMYN | 0.40 | 3.03 | 0.13 | 0 |
| COX2 | *H.schachtii* | *P.chalcoensis* | GMYN | 0.13 | 2.43 | 0.05 | 0 |
| COX2 | *H.schachtii* | *S.stercoralis* | GMYN | 0.39 | 2.83 | 0.14 | 0 |
| COX2 | *H.schachtii* | *U.sanguinis-haplo45* | GMYN | 0.39 | 3.04 | 0.13 | 0 |
| COX2 | *H.trifolii* | *Caenorhabditis-sp* | GMYN | 0.44 | 2.86 | 0.15 | 0 |
| COX2 | *H.trifolii* | *G.ellingtonae-chr1* | GMYN | 0.24 | 3.38 | 0.07 | 0 |
| COX2 | *H.trifolii* | *H.cardiolata* | GMYN | 0.13 | 2.38 | 0.05 | 0 |
| COX2 | *H.trifolii* | *H.glycines* | GMYN | 0.03 | 3.08 | 0.01 | 7.08902E-52 |
| COX2 | *H.trifolii* | *K.sudhausi* | GMYN | 0.41 | 3.07 | 0.14 | 0 |
| COX2 | *H.trifolii* | *Litoditis-aff.marina-Pml* | GMYN | 0.41 | 2.85 | 0.14 | 0 |
| COX2 | *H.trifolii* | *O.columbianum* | GMYN | 0.44 | 2.93 | 0.15 | 0 |
| COX2 | *H.trifolii* | *P.chalcoensis* | GMYN | 0.14 | 2.38 | 0.06 | 0 |
| COX2 | *H.trifolii* | *S.stercoralis* | GMYN | 0.40 | 2.77 | 0.14 | 0 |
| COX2 | *H.trifolii* | *U.sanguinis-haplo45* | GMYN | 0.42 | 2.94 | 0.14 | 0 |
| COX2 | *H.glycines* | *Caenorhabditis-sp* | GMYN | 0.40 | 2.91 | 0.14 | 0 |
| COX2 | *H.glycines* | *G.ellingtonae-chr1* | GMYN | 0.23 | 3.40 | 0.07 | 1.99862E-50 |
| COX2 | *H.glycines* | *H.cardiolata* | GMYN | 0.13 | 2.44 | 0.05 | 0 |
| COX2 | *H.glycines* | *K.sudhausi* | GMYN | 0.40 | 3.14 | 0.13 | 3.88428E-24 |
| COX2 | *H.glycines* | *Litoditis-aff.marina-Pml* | GMYN | 0.37 | 2.93 | 0.13 | 0 |
| COX2 | *H.glycines* | *O.columbianum* | GMYN | 0.40 | 3.02 | 0.13 | 0 |
| COX2 | *H.glycines* | *P.chalcoensis* | GMYN | 0.13 | 2.43 | 0.05 | 0 |
| COX2 | *H.glycines* | *S.stercoralis* | GMYN | 0.38 | 2.76 | 0.14 | 0 |
| COX2 | *H.glycines* | *U.sanguinis-haplo45* | GMYN | 0.39 | 3.02 | 0.13 | 0 |
| COX2 | *H.cardiolata* | *Caenorhabditis-sp* | GMYN | 0.33 | 2.32 | 0.14 | 0 |
| COX2 | *H.cardiolata* | *G.ellingtonae-chr1* | GMYN | 0.17 | 2.79 | 0.06 | 0 |
| COX2 | *H.cardiolata* | *K.sudhausi* | GMYN | 0.32 | 2.49 | 0.13 | 0 |
| COX2 | *H.cardiolata* | *Litoditis-aff.marina-Pml* | GMYN | 0.34 | 2.45 | 0.14 | 0 |
| COX2 | *H.cardiolata* | *O.columbianum* | GMYN | 0.33 | 2.35 | 0.14 | 0 |
| COX2 | *H.cardiolata* | *P.chalcoensis* | GMYN | 0.12 | 2.17 | 0.06 | 0 |
| COX2 | *H.cardiolata* | *S.stercoralis* | GMYN | 0.35 | 2.14 | 0.16 | 0 |
| COX2 | *P.chalcoensis* | *Caenorhabditis-sp* | GMYN | 0.28 | 2.26 | 0.12 | 0 |
| COX2 | *P.chalcoensis* | *G.ellingtonae-chr1* | GMYN | 0.11 | 2.77 | 0.04 | 0 |
| COX2 | *P.chalcoensis* | *Litoditis-aff.marina-Pml* | GMYN | 0.27 | 2.32 | 0.12 | 0 |
| COX2 | *P.chalcoensis* | *O.columbianum* | GMYN | 0.27 | 2.22 | 0.12 | 0 |
| COX2 | *P.chalcoensis* | *S.stercoralis* | GMYN | 0.31 | 2.17 | 0.14 | 0 |

| | | | | | | | |
|---|---|---|---|---|---|---|---|
| COX2 | *P.chalcoensis* | *U.sanguinis-haplo45* | GMYN | 0.29 | 2.44 | 0.12 | 0 |
| COX2 | *O.columbianum* | *Caenorhabditis-sp* | GMYN | 0.10 | 3.18 | 0.03 | 8.39697E-52 |
| COX2 | *O.columbianum* | *G.ellingtonae-chr1* | GMYN | 0.40 | 3.42 | 0.12 | 3.81895E-29 |
| COX2 | *O.columbianum* | *K.sudhausi* | GMYN | 0.12 | 3.19 | 0.04 | 0 |
| COX2 | *O.columbianum* | *Litoditis-aff.marina-Pml* | GMYN | 0.07 | 3.17 | 0.02 | 0 |
| COX2 | *O.columbianum* | *S.stercoralis* | GMYN | 0.16 | 2.93 | 0.05 | 0 |
| COX2 | *O.columbianum* | *U.sanguinis-haplo45* | GMYN | 0.04 | 3.24 | 0.01 | 5.40224E-67 |
| COX2 | *K.sudhausi* | *Caenorhabditis-sp* | GMYN | 0.10 | 3.12 | 0.03 | 0 |
| COX2 | *K.sudhausi* | *G.ellingtonae-chr1* | GMYN | 0.43 | 3.51 | 0.12 | 6.33492E-26 |
| COX2 | *K.sudhausi* | *Litoditis-aff.marina-Pml* | GMYN | 0.08 | 3.08 | 0.03 | 0 |
| COX2 | *K.sudhausi* | *S.stercoralis* | GMYN | 0.21 | 3.10 | 0.07 | 0 |
| COX2 | *K.sudhausi* | *U.sanguinis-haplo45* | GMYN | 0.12 | 3.26 | 0.04 | 0 |
| COX2 | *Caenorhabditis-sp* | *G.ellingtonae-chr1* | GMYN | 0.40 | 3.37 | 0.12 | 0 |
| COX2 | *Caenorhabditis-sp* | *Litoditis-aff.marina-Pml* | GMYN | 0.06 | 3.08 | 0.02 | 9.11038E-59 |
| COX2 | *Caenorhabditis-sp* | *S.stercoralis* | GMYN | 0.19 | 2.94 | 0.06 | 0 |
| COX2 | *Caenorhabditis-sp* | *U.sanguinis-haplo45* | GMYN | 0.10 | 3.15 | 0.03 | 0 |
| COX2 | *U.sanguinis-haplo45* | *G.ellingtonae-chr1* | GMYN | 0.42 | 3.46 | 0.12 | 1.98059E-31 |
| COX2 | *U.sanguinis-haplo45* | *Litoditis-aff.marina-Pml* | GMYN | 0.08 | 3.12 | 0.02 | 0 |
| COX2 | *U.sanguinis-haplo45* | *S.stercoralis* | GMYN | 0.18 | 3.02 | 0.06 | 0 |
| COX2 | *Litoditis-aff.marina-Pml* | *G.ellingtonae-chr1* | GMYN | 0.43 | 3.41 | 0.13 | 5.35132E-39 |
| COX2 | *Litoditis-aff.marina-Pml* | *S.stercoralis* | GMYN | 0.18 | 2.94 | 0.06 | 0 |
| COX2 | *G.ellingtonae-chr1* | *S.stercoralis* | GMYN | 0.35 | 3.20 | 0.11 | 0 |
| COX3 | *H.schachtii* | *Caenorhabditis-sp* | GMYN | 0.45 | 2.96 | 0.15 | 0 |
| COX3 | *H.schachtii* | *G.ellingtonae-chr1* | GMYN | 0.29 | 3.26 | 0.09 | 0 |
| COX3 | *H.schachtii* | *H.cardiolata* | GMYN | 0.24 | 2.93 | 0.08 | 0 |
| COX3 | *H.schachtii* | *H.glycines* | GMYN | 0.04 | 0.72 | 0.06 | 1.88799E-23 |
| COX3 | *H.schachtii* | *H.trifolii* | GMYN | 0.07 | 1.96 | 0.03 | 1.86869E-33 |
| COX3 | *H.schachtii* | *K.sudhausi* | GMYN | 0.43 | 2.96 | 0.14 | 0 |
| COX3 | *H.schachtii* | *Litoditis-aff.marina-Pml* | GMYN | 0.48 | 2.89 | 0.17 | 0 |
| COX3 | *H.schachtii* | *O.columbianum* | GMYN | 0.45 | 2.93 | 0.15 | 0 |
| COX3 | *H.schachtii* | *P.chalcoensis* | GMYN | 0.26 | 2.89 | 0.09 | 0 |
| COX3 | *H.schachtii* | *S.stercoralis* | GMYN | 0.36 | 2.84 | 0.13 | 0 |

| | | | | | | | |
|---|---|---|---|---|---|---|---|
| COX3 | *H.schachtii* | *U.sanguinis-haplo45* | GMYN | 0.46 | 3.02 | 0.15 | 0 |
| COX3 | *H.trifolii* | *Caenorhabditis-sp* | GMYN | 0.45 | 3.00 | 0.15 | 0 |
| COX3 | *H.trifolii* | *G.ellingtonae-chr1* | GMYN | 0.29 | 3.24 | 0.09 | 0 |
| COX3 | *H.trifolii* | *H.cardiolata* | GMYN | 0.24 | 2.87 | 0.09 | 0 |
| COX3 | *H.trifolii* | *H.glycines* | GMYN | 0.05 | 2.74 | 0.02 | 3.96938E-44 |
| COX3 | *H.trifolii* | *K.sudhausi* | GMYN | 0.43 | 2.98 | 0.14 | 0 |
| COX3 | *H.trifolii* | *Litoditis-aff.marina-Pml* | GMYN | 0.48 | 2.94 | 0.16 | 0 |
| COX3 | *H.trifolii* | *O.columbianum* | GMYN | 0.46 | 2.98 | 0.15 | 0 |
| COX3 | *H.trifolii* | *P.chalcoensis* | GMYN | 0.25 | 2.91 | 0.09 | 0 |
| COX3 | *H.trifolii* | *S.stercoralis* | GMYN | 0.37 | 2.81 | 0.13 | 0 |
| COX3 | *H.trifolii* | *U.sanguinis-haplo45* | GMYN | 0.47 | 3.09 | 0.15 | 0 |
| COX3 | *H.glycines* | *Caenorhabditis-sp* | GMYN | 0.45 | 2.94 | 0.15 | 0 |
| COX3 | *H.glycines* | *G.ellingtonae-chr1* | GMYN | 0.29 | 3.21 | 0.09 | 0 |
| COX3 | *H.glycines* | *H.cardiolata* | GMYN | 0.22 | 2.83 | 0.08 | 0 |
| COX3 | *H.glycines* | *K.sudhausi* | GMYN | 0.43 | 2.96 | 0.14 | 0 |
| COX3 | *H.glycines* | *Litoditis-aff.marina-Pml* | GMYN | 0.47 | 2.90 | 0.16 | 0 |
| COX3 | *H.glycines* | *O.columbianum* | GMYN | 0.45 | 2.94 | 0.15 | 0 |
| COX3 | *H.glycines* | *P.chalcoensis* | GMYN | 0.26 | 2.80 | 0.09 | 0 |
| COX3 | *H.glycines* | *S.stercoralis* | GMYN | 0.37 | 2.81 | 0.13 | 0 |
| COX3 | *H.glycines* | *U.sanguinis-haplo45* | GMYN | 0.46 | 2.99 | 0.16 | 0 |
| COX3 | *H.cardiolata* | *Caenorhabditis-sp* | GMYN | 0.39 | 2.75 | 0.14 | 0 |
| COX3 | *H.cardiolata* | *G.ellingtonae-chr1* | GMYN | 0.29 | 3.16 | 0.09 | 0 |
| COX3 | *H.cardiolata* | *K.sudhausi* | GMYN | 0.39 | 2.85 | 0.14 | 0 |
| COX3 | *H.cardiolata* | *Litoditis-aff.marina-Pml* | GMYN | 0.41 | 2.69 | 0.15 | 0 |
| COX3 | *H.cardiolata* | *O.columbianum* | GMYN | 0.41 | 2.76 | 0.15 | 0 |
| COX3 | *H.cardiolata* | *P.chalcoensis* | GMYN | 0.21 | 2.60 | 0.08 | 0 |
| COX3 | *H.cardiolata* | *S.stercoralis* | GMYN | 0.32 | 2.54 | 0.13 | 0 |
| COX3 | *H.cardiolata* | *U.sanguinis-haplo45* | GMYN | 0.38 | 2.84 | 0.14 | 0 |
| COX3 | *P.chalcoensis* | *G.ellingtonae-chr1* | GMYN | 0.21 | 3.24 | 0.07 | 0 |
| COX3 | *P.chalcoensis* | *K.sudhausi* | GMYN | 0.34 | 3.00 | 0.11 | 0 |
| COX3 | *P.chalcoensis* | *Litoditis-aff.marina-Pml* | GMYN | 0.35 | 2.87 | 0.12 | 0 |
| COX3 | *P.chalcoensis* | *O.columbianum* | GMYN | 0.35 | 2.97 | 0.12 | 0 |
| COX3 | *O.columbianum* | *Caenorhabditis-sp* | GMYN | 0.06 | 3.24 | 0.02 | 0 |
| COX3 | *O.columbianum* | *G.ellingtonae-chr1* | GMYN | 0.47 | 3.36 | 0.14 | 0 |
| COX3 | *O.columbianum* | *K.sudhausi* | GMYN | 0.09 | 3.26 | 0.03 | 0 |
| COX3 | *O.columbianum* | *Litoditis-aff.marina-Pml* | GMYN | 0.07 | 3.23 | 0.02 | 5.4594E-62 |

| | | | | | | | |
|---|---|---|---|---|---|---|---|
| COX3 | *O.columbianum* | *S.stercoralis* | GMYN | 0.24 | 2.86 | 0.08 | 0 |
| COX3 | *O.columbianum* | *U.sanguinis-haplo45* | GMYN | 0.04 | 3.35 | 0.01 | 6.94592E-89 |
| COX3 | *K.sudhausi* | *Caenorhabditis-sp* | GMYN | 0.07 | 3.07 | 0.02 | 0 |
| COX3 | *K.sudhausi* | *G.ellingtonae-chr1* | GMYN | 0.42 | 3.32 | 0.13 | 0 |
| COX3 | *K.sudhausi* | *Litoditis-aff.marina-Pml* | GMYN | 0.08 | 3.16 | 0.02 | 0 |
| COX3 | *K.sudhausi* | *S.stercoralis* | GMYN | 0.22 | 2.86 | 0.08 | 0 |
| COX3 | *K.sudhausi* | *U.sanguinis-haplo45* | GMYN | 0.09 | 3.31 | 0.03 | 0 |
| COX3 | *Caenorhabditis-sp* | *G.ellingtonae-chr1* | GMYN | 0.44 | 3.32 | 0.13 | 0 |
| COX3 | *Caenorhabditis-sp* | *Litoditis-aff.marina-Pml* | GMYN | 0.05 | 3.16 | 0.01 | 3.31124E-62 |
| COX3 | *Caenorhabditis-sp* | *S.stercoralis* | GMYN | 0.23 | 2.80 | 0.08 | 0 |
| COX3 | *Caenorhabditis-sp* | *U.sanguinis-haplo45* | GMYN | 0.07 | 3.25 | 0.02 | 0 |
| COX3 | *U.sanguinis-haplo45* | *G.ellingtonae-chr1* | GMYN | 0.45 | 3.42 | 0.13 | 8.5733E-42 |
| COX3 | *U.sanguinis-haplo45* | *Litoditis-aff.marina-Pml* | GMYN | 0.08 | 3.31 | 0.02 | 3.71667E-62 |
| COX3 | *U.sanguinis-haplo45* | *S.stercoralis* | GMYN | 0.23 | 2.93 | 0.08 | 0 |
| COX3 | *Litoditis-aff.marina-Pml* | *G.ellingtonae-chr1* | GMYN | 0.46 | 3.31 | 0.14 | 0 |
| COX3 | *Litoditis-aff.marina-Pml* | *S.stercoralis* | GMYN | 0.21 | 2.75 | 0.08 | 0 |
| COX3 | *G.ellingtonae-chr1* | *S.stercoralis* | GMYN | 0.36 | 2.96 | 0.12 | 0 |
| CYTB | *H.schachtii* | *Caenorhabditis-sp* | GMYN | 0.39 | 3.34 | 0.12 | 0 |
| CYTB | *H.schachtii* | *G.ellingtonae-chr1* | GMYN | 0.23 | 3.77 | 0.06 | 0 |
| CYTB | *H.schachtii* | *H.glycines* | GMYN | 0.03 | 0.63 | 0.05 | 2.57853E-35 |
| CYTB | *H.schachtii* | *H.trifolii* | GMYN | 0.05 | 1.22 | 0.04 | 4.6446E-53 |
| CYTB | *H.schachtii* | *K.sudhausi* | GMYN | 0.41 | 3.41 | 0.12 | 0 |
| CYTB | *H.schachtii* | *Litoditis-aff.marina-Pml* | GMYN | 0.41 | 3.33 | 0.12 | 0 |
| CYTB | *H.schachtii* | *O.columbianum* | GMYN | 0.39 | 3.31 | 0.12 | 0 |
| CYTB | *H.schachtii* | *P.chalcoensis* | GMYN | 0.15 | 3.01 | 0.05 | 0 |
| CYTB | *H.schachtii* | *S.stercoralis* | GMYN | 0.34 | 3.20 | 0.11 | 0 |
| CYTB | *H.schachtii* | *U.sanguinis-haplo45* | GMYN | 0.40 | 3.31 | 0.12 | 0 |
| CYTB | *H.trifolii* | *Caenorhabditis-sp* | GMYN | 0.39 | 3.27 | 0.12 | 0 |
| CYTB | *H.trifolii* | *G.ellingtonae-chr1* | GMYN | 0.22 | 3.69 | 0.06 | 0 |
| CYTB | *H.trifolii* | *H.glycines* | GMYN | 0.05 | 1.79 | 0.03 | 5.1676E-59 |
| CYTB | *H.trifolii* | *K.sudhausi* | GMYN | 0.41 | 3.33 | 0.12 | 0 |
| CYTB | *H.trifolii* | *Litoditis-aff.marina-Pml* | GMYN | 0.41 | 3.25 | 0.13 | 0 |

| | | | | | | | |
|---|---|---|---|---|---|---|---|
| CYTB | *H.trifolii* | *O.columbianum* | GMYN | 0.41 | 3.30 | 0.13 | 0 |
| CYTB | *H.trifolii* | *P.chalcoensis* | GMYN | 0.16 | 2.92 | 0.05 | 0 |
| CYTB | *H.trifolii* | *S.stercoralis* | GMYN | 0.35 | 3.13 | 0.11 | 0 |
| CYTB | *H.trifolii* | *U.sanguinis-haplo45* | GMYN | 0.41 | 3.24 | 0.13 | 0 |
| CYTB | *H.glycines* | *Caenorhabditis-sp* | GMYN | 0.38 | 3.31 | 0.12 | 0 |
| CYTB | *H.glycines* | *G.ellingtonae-chr1* | GMYN | 0.23 | 3.76 | 0.06 | 5.53526E-75 |
| CYTB | *H.glycines* | *K.sudhausi* | GMYN | 0.39 | 3.38 | 0.11 | 0 |
| CYTB | *H.glycines* | *Litoditis-aff.marina-Pml* | GMYN | 0.41 | 3.30 | 0.12 | 0 |
| CYTB | *H.glycines* | *O.columbianum* | GMYN | 0.39 | 3.31 | 0.12 | 0 |
| CYTB | *H.glycines* | *P.chalcoensis* | GMYN | 0.16 | 3.03 | 0.05 | 1.36265E-29 |
| CYTB | *H.glycines* | *S.stercoralis* | GMYN | 0.34 | 3.21 | 0.11 | 0 |
| CYTB | *H.glycines* | *U.sanguinis-haplo45* | GMYN | 0.40 | 3.31 | 0.12 | 0 |
| CYTB | *P.chalcoensis* | *Caenorhabditis-sp* | GMYN | 0.37 | 2.84 | 0.13 | 0 |
| CYTB | *P.chalcoensis* | *G.ellingtonae-chr1* | GMYN | 0.18 | 3.33 | 0.05 | 0 |
| CYTB | *P.chalcoensis* | *K.sudhausi* | GMYN | 0.38 | 2.89 | 0.13 | 0 |
| CYTB | *P.chalcoensis* | *Litoditis-aff.marina-Pml* | GMYN | 0.37 | 2.74 | 0.14 | 0 |
| CYTB | *P.chalcoensis* | *O.columbianum* | GMYN | 0.37 | 2.84 | 0.13 | 0 |
| CYTB | *P.chalcoensis* | *S.stercoralis* | GMYN | 0.33 | 2.48 | 0.13 | 0 |
| CYTB | *P.chalcoensis* | *U.sanguinis-haplo45* | GMYN | 0.35 | 2.82 | 0.12 | 0 |
| CYTB | *O.columbianum* | *Caenorhabditis-sp* | GMYN | 0.13 | 3.60 | 0.04 | 0 |
| CYTB | *O.columbianum* | *G.ellingtonae-chr1* | GMYN | 0.44 | 3.68 | 0.12 | 9.41013E-47 |
| CYTB | *O.columbianum* | *K.sudhausi* | GMYN | 0.13 | 3.67 | 0.04 | 1.01134E-89 |
| CYTB | *O.columbianum* | *Litoditis-aff.marina-Pml* | GMYN | 0.09 | 3.62 | 0.02 | 5.59138E-74 |
| CYTB | *O.columbianum* | *S.stercoralis* | GMYN | 0.24 | 3.19 | 0.08 | 0 |
| CYTB | *O.columbianum* | *U.sanguinis-haplo45* | GMYN | 0.09 | 3.47 | 0.03 | 4.3462E-67 |
| CYTB | *K.sudhausi* | *Caenorhabditis-sp* | GMYN | 0.12 | 3.59 | 0.03 | 0 |
| CYTB | *K.sudhausi* | *G.ellingtonae-chr1* | GMYN | 0.46 | 3.71 | 0.12 | 1.62448E-54 |
| CYTB | *K.sudhausi* | *Litoditis-aff.marina-Pml* | GMYN | 0.11 | 3.60 | 0.03 | 0 |
| CYTB | *K.sudhausi* | *S.stercoralis* | GMYN | 0.24 | 3.36 | 0.07 | 0 |
| CYTB | *K.sudhausi* | *U.sanguinis-haplo45* | GMYN | 0.14 | 3.67 | 0.04 | 3.08176E-90 |
| CYTB | *Caenorhabditis-sp* | *G.ellingtonae-chr1* | GMYN | 0.44 | 3.65 | 0.12 | 0 |
| CYTB | *Caenorhabditis-sp* | *Litoditis-aff.marina-Pml* | GMYN | 0.08 | 3.50 | 0.02 | 0 |
| CYTB | *Caenorhabditis-sp* | *S.stercoralis* | GMYN | 0.23 | 3.24 | 0.07 | 0 |
| CYTB | *Caenorhabditis-sp* | *U.sanguinis-haplo45* | GMYN | 0.15 | 3.61 | 0.04 | 1.65357E-76 |

| | | | | | | | |
|---|---|---|---|---|---|---|---|
| CYTB | *U.sanguinis-haplo45* | *G.ellingtonae-chr1* | GMYN | 0.43 | 3.66 | 0.12 | 0 |
| CYTB | *U.sanguinis-haplo45* | *Litoditis-aff.marina-Pml* | GMYN | 0.11 | 3.59 | 0.03 | 3.50843E-78 |
| CYTB | *U.sanguinis-haplo45* | *S.stercoralis* | GMYN | 0.25 | 3.25 | 0.08 | 0 |
| CYTB | *Litoditis-aff.marina-Pml* | *G.ellingtonae-chr1* | GMYN | 0.43 | 3.64 | 0.12 | 0 |
| CYTB | *Litoditis-aff.marina-Pml* | *S.stercoralis* | GMYN | 0.25 | 3.23 | 0.08 | 0 |
| CYTB | *G.ellingtonae-chr1* | *S.stercoralis* | GMYN | 0.37 | 3.45 | 0.11 | 0 |
| ND1 | *H.schachtii* | *Caenorhabditis-sp* | GMYN | 0.51 | 3.10 | 0.16 | 0 |
| ND1 | *H.schachtii* | *H.glycines* | GMYN | 0.08 | 3.46 | 0.02 | 2.32223E-39 |
| ND1 | *H.schachtii* | *H.trifolii* | GMYN | 0.07 | 1.59 | 0.05 | 1.80686E-38 |
| ND1 | *H.schachtii* | *K.sudhausi* | GMYN | 0.56 | 3.23 | 0.17 | 0 |
| ND1 | *H.schachtii* | *Litoditis-aff.marina-Pml* | GMYN | 0.56 | 3.06 | 0.18 | 0 |
| ND1 | *H.schachtii* | *O.columbianum* | GMYN | 0.52 | 3.10 | 0.17 | 0 |
| ND1 | *H.schachtii* | *S.stercoralis* | GMYN | 0.43 | 2.94 | 0.15 | 0 |
| ND1 | *H.schachtii* | *U.sanguinis-haplo45* | GMYN | 0.52 | 3.18 | 0.16 | 0 |
| ND1 | *H.trifolii* | *Caenorhabditis-sp* | GMYN | 0.53 | 3.12 | 0.17 | 3.19212E-23 |
| ND1 | *H.trifolii* | *H.glycines* | GMYN | 0.12 | 3.19 | 0.04 | 2.0347E-41 |
| ND1 | *H.trifolii* | *K.sudhausi* | GMYN | 0.56 | 3.20 | 0.18 | 1.5201E-28 |
| ND1 | *H.trifolii* | *Litoditis-aff.marina-Pml* | GMYN | 0.56 | 3.05 | 0.18 | 1.843E-23 |
| ND1 | *H.trifolii* | *O.columbianum* | GMYN | 0.51 | 3.04 | 0.17 | 0 |
| ND1 | *H.trifolii* | *S.stercoralis* | GMYN | 0.45 | 2.84 | 0.16 | 0 |
| ND1 | *H.trifolii* | *U.sanguinis-haplo45* | GMYN | 0.55 | 3.19 | 0.17 | 7.95134E-19 |
| ND1 | *H.glycines* | *Caenorhabditis-sp* | GMYN | 0.49 | 3.08 | 0.16 | 0 |
| ND1 | *H.glycines* | *K.sudhausi* | GMYN | 0.55 | 3.10 | 0.18 | 2.86883E-23 |
| ND1 | *H.glycines* | *Litoditis-aff.marina-Pml* | GMYN | 0.53 | 2.96 | 0.18 | 0 |
| ND1 | *H.glycines* | *O.columbianum* | GMYN | 0.52 | 3.07 | 0.17 | 0 |
| ND1 | *H.glycines* | *S.stercoralis* | GMYN | 0.44 | 2.76 | 0.16 | 0 |
| ND1 | *H.glycines* | *U.sanguinis-haplo45* | GMYN | 0.52 | 3.08 | 0.17 | 0 |
| ND1 | *O.columbianum* | *Caenorhabditis-sp* | GMYN | 0.15 | 3.41 | 0.05 | 8.55589E-55 |
| ND1 | *O.columbianum* | *K.sudhausi* | GMYN | 0.16 | 3.39 | 0.05 | 1.09865E-62 |
| ND1 | *O.columbianum* | *Litoditis-aff.marina-Pml* | GMYN | 0.13 | 3.42 | 0.04 | 4.63399E-46 |
| ND1 | *O.columbianum* | *S.stercoralis* | GMYN | 0.22 | 3.17 | 0.07 | 0 |
| ND1 | *O.columbianum* | *U.sanguinis-haplo45* | GMYN | 0.09 | 0.78 | 0.11 | 4.92637E-28 |
| ND1 | *K.sudhausi* | *Caenorhabditis-sp* | GMYN | 0.15 | 3.40 | 0.04 | 3.81295E-53 |

| | | | | | | | |
|---|---|---|---|---|---|---|---|
| ND1 | *K.sudhausi* | *Litoditis-aff.marina-Pml* | GMYN | 0.14 | 3.35 | 0.04 | 2.00354E-57 |
| ND1 | *K.sudhausi* | *S.stercoralis* | GMYN | 0.22 | 3.25 | 0.07 | 0 |
| ND1 | *K.sudhausi* | *U.sanguinis-haplo45* | GMYN | 0.19 | 3.53 | 0.05 | 3.17015E-53 |
| ND1 | *Caenorhabditis-sp* | *Litoditis-aff.marina-Pml* | GMYN | 0.09 | 3.18 | 0.03 | 0 |
| ND1 | *Caenorhabditis-sp* | *S.stercoralis* | GMYN | 0.20 | 3.07 | 0.07 | 0 |
| ND1 | *Caenorhabditis-sp* | *U.sanguinis-haplo45* | GMYN | 0.14 | 3.45 | 0.04 | 1.39704E-66 |
| ND1 | *U.sanguinis-haplo45* | *Litoditis-aff.marina-Pml* | GMYN | 0.13 | 3.43 | 0.04 | 1.00956E-59 |
| ND1 | *U.sanguinis-haplo45* | *S.stercoralis* | GMYN | 0.22 | 3.27 | 0.07 | 0 |
| ND1 | *Litoditis-aff.marina-Pml* | *S.stercoralis* | GMYN | 0.22 | 3.13 | 0.07 | 0 |
| ND2 | *H.schachtii* | *Caenorhabditis-sp* | GMYN | 0.65 | 2.94 | 0.22 | 4.33723E-19 |
| ND2 | *H.schachtii* | *G.ellingtonae-chr1* | GMYN | 0.35 | 2.29 | 0.15 | 1.26784E-18 |
| ND2 | *H.schachtii* | *H.cardiolata* | GMYN | 0.23 | 2.88 | 0.08 | 1.91557E-17 |
| ND2 | *H.schachtii* | *H.glycines* | GMYN | 0.06 | 0.98 | 0.06 | 8.70763E-22 |
| ND2 | *H.schachtii* | *H.trifolii* | GMYN | 0.05 | 0.73 | 0.06 | 8.74168E-25 |
| ND2 | *H.schachtii* | *K.sudhausi* | GMYN | 0.72 | 2.97 | 0.24 | 1.27007E-08 |
| ND2 | *H.schachtii* | *Litoditis-aff.marina-Pml* | GMYN | 0.66 | 3.02 | 0.22 | 1.53111E-09 |
| ND2 | *H.schachtii* | *O.columbianum* | GMYN | 0.70 | 3.02 | 0.23 | 5.24686E-09 |
| ND2 | *H.schachtii* | *P.chalcoensis* | GMYN | 0.33 | 1.37 | 0.24 | 1.62112E-06 |
| ND2 | *H.schachtii* | *S.stercoralis* | GMYN | 0.49 | 2.68 | 0.18 | 0 |
| ND2 | *H.trifolii* | *Caenorhabditis-sp* | GMYN | 0.68 | 2.97 | 0.23 | 1.16616E-13 |
| ND2 | *H.trifolii* | *G.ellingtonae-chr1* | GMYN | 0.36 | 3.49 | 0.10 | 7.87097E-22 |
| ND2 | *H.trifolii* | *H.cardiolata* | GMYN | 0.24 | 2.82 | 0.09 | 6.97699E-17 |
| ND2 | *H.trifolii* | *H.glycines* | GMYN | 0.07 | 1.55 | 0.04 | 2.00479E-31 |
| ND2 | *H.trifolii* | *Litoditis-aff.marina-Pml* | GMYN | 0.64 | 3.05 | 0.21 | 3.73521E-09 |
| ND2 | *H.trifolii* | *O.columbianum* | GMYN | 0.71 | 2.49 | 0.29 | 2.80683E-06 |
| ND2 | *H.trifolii* | *P.chalcoensis* | GMYN | 0.32 | 1.02 | 0.32 | 4.55093E-05 |
| ND2 | *H.trifolii* | *S.stercoralis* | GMYN | 0.50 | 2.75 | 0.18 | 0 |
| ND2 | *H.trifolii* | *U.sanguinis-haplo45* | GMYN | 0.67 | 4.03 | 0.17 | 9.50997E-08 |
| ND2 | *H.glycines* | *Caenorhabditis-sp* | GMYN | 0.64 | 3.11 | 0.21 | 1.32509E-10 |
| ND2 | *H.glycines* | *G.ellingtonae-chr1* | GMYN | 0.39 | 1.53 | 0.26 | 3.50068E-13 |
| ND2 | *H.glycines* | *H.cardiolata* | GMYN | 0.27 | 1.87 | 0.15 | 6.54392E-10 |
| ND2 | *H.glycines* | *K.sudhausi* | GMYN | 0.74 | 2.96 | 0.25 | 3.72258E-13 |
| ND2 | *H.glycines* | *Litoditis-aff.marina-Pml* | GMYN | 0.74 | 0.78 | 0.95 | 0.788344 |
| ND2 | *H.glycines* | *O.columbianum* | GMYN | 0.67 | 3.04 | 0.22 | 1.21295E-07 |

| | | | | | | | |
|---|---|---|---|---|---|---|---|
| ND2 | *H.glycines* | *S.stercoralis* | GMYN | 0.52 | 2.70 | 0.19 | 0 |
| ND2 | *H.glycines* | *U.sanguinis-haplo45* | GMYN | 0.70 | 1.73 | 0.40 | 6.51584E-05 |
| ND2 | *H.cardiolata* | *Caenorhabditis-sp* | GMYN | 0.66 | 2.76 | 0.24 | 8.97185E-12 |
| ND2 | *H.cardiolata* | *G.ellingtonae-chr1* | GMYN | 0.40 | 1.59 | 0.25 | 5.50819E-12 |
| ND2 | *H.cardiolata* | *K.sudhausi* | GMYN | 0.67 | 1.35 | 0.49 | 0.00160914 |
| ND2 | *H.cardiolata* | *Litoditis-aff.marina-Pml* | GMYN | 0.65 | 1.77 | 0.37 | 0.000159098 |
| ND2 | *H.cardiolata* | *O.columbianum* | GMYN | 0.65 | 3.05 | 0.21 | 0.00141443 |
| ND2 | *H.cardiolata* | *P.chalcoensis* | GMYN | 0.29 | 1.36 | 0.21 | 1.06771E-05 |
| ND2 | *H.cardiolata* | *S.stercoralis* | GMYN | 0.46 | 2.32 | 0.20 | 0 |
| ND2 | *H.cardiolata* | *U.sanguinis-haplo45* | GMYN | 0.64 | 1.70 | 0.38 | 0.000156924 |
| ND2 | *P.chalcoensis* | *Caenorhabditis-sp* | GMYN | 0.65 | 2.97 | 0.22 | 1.54254E-15 |
| ND2 | *P.chalcoensis* | *G.ellingtonae-chr1* | GMYN | 0.37 | 1.30 | 0.28 | 7.10984E-09 |
| ND2 | *P.chalcoensis* | *K.sudhausi* | GMYN | 0.72 | 2.82 | 0.25 | 0 |
| ND2 | *P.chalcoensis* | *Litoditis-aff.marina-Pml* | GMYN | 0.68 | 2.92 | 0.23 | 4.71243E-11 |
| ND2 | *P.chalcoensis* | *O.columbianum* | GMYN | 0.70 | 3.00 | 0.23 | 7.14998E-12 |
| ND2 | *P.chalcoensis* | *S.stercoralis* | GMYN | 0.51 | 2.78 | 0.18 | 6.15479E-10 |
| ND2 | *P.chalcoensis* | *U.sanguinis-haplo45* | GMYN | 0.70 | 1.59 | 0.44 | 7.38463E-05 |
| ND2 | *O.columbianum* | *Caenorhabditis-sp* | GMYN | 0.36 | 0.60 | 0.59 | 0.00930693 |
| ND2 | *O.columbianum* | *G.ellingtonae-chr1* | GMYN | 0.77 | 1.33 | 0.58 | 0.029801 |
| ND2 | *O.columbianum* | *K.sudhausi* | GMYN | 0.42 | 1.00 | 0.42 | 4.78964E-05 |
| ND2 | *O.columbianum* | *Litoditis-aff.marina-Pml* | GMYN | 0.30 | 1.95 | 0.15 | 1.06266E-14 |
| ND2 | *O.columbianum* | *S.stercoralis* | GMYN | 0.51 | 2.20 | 0.23 | 2.16655E-09 |
| ND2 | *O.columbianum* | *U.sanguinis-haplo45* | GMYN | 0.19 | 0.62 | 0.30 | 1.9592E-07 |
| ND2 | *K.sudhausi* | *Caenorhabditis-sp* | GMYN | 0.42 | 2.57 | 0.16 | 5.81464E-10 |
| ND2 | *K.sudhausi* | *G.ellingtonae-chr1* | GMYN | 0.72 | 1.98 | 0.37 | 3.22894E-07 |
| ND2 | *K.sudhausi* | *Litoditis-aff.marina-Pml* | GMYN | 0.45 | 1.93 | 0.23 | 1.42171E-07 |
| ND2 | *K.sudhausi* | *S.stercoralis* | GMYN | 0.49 | 3.16 | 0.16 | 1.99385E-21 |
| ND2 | *K.sudhausi* | *U.sanguinis-haplo45* | GMYN | 0.44 | 1.20 | 0.36 | 4.2253E-06 |
| ND2 | *Caenorhabditis-sp* | *G.ellingtonae-chr1* | GMYN | 0.59 | 3.42 | 0.17 | 3.19013E-13 |
| ND2 | *Caenorhabditis-sp* | *Litoditis-aff.marina-Pml* | GMYN | 0.20 | 3.23 | 0.06 | 3.5777E-49 |
| ND2 | *Caenorhabditis-sp* | *S.stercoralis* | GMYN | 0.42 | 3.04 | 0.14 | 0 |
| ND2 | *Caenorhabditis-sp* | *U.sanguinis-haplo45* | GMYN | 0.34 | 1.43 | 0.24 | 9.61387E-10 |
| ND2 | *U.sanguinis-haplo45* | *G.ellingtonae-chr1* | GMYN | 0.68 | 1.07 | 0.63 | 0.0274594 |

| | | | | | | | |
|---|---|---|---|---|---|---|---|
| ND2 | *U.sanguinis-haplo45* | *Litoditis-aff.marina-Pml* | GMYN | 0.30 | 4.83 | 0.06 | 2.07024E-22 |
| ND2 | *U.sanguinis-haplo45* | *S.stercoralis* | GMYN | 0.50 | 1.78 | 0.28 | 6.32236E-09 |
| ND2 | *Litoditis-aff.marina-Pml* | *G.ellingtonae-chr1* | GMYN | 0.72 | 1.80 | 0.40 | 8.80827E-07 |
| ND2 | *Litoditis-aff.marina-Pml* | *S.stercoralis* | GMYN | 0.47 | 3.17 | 0.15 | 4.25268E-28 |
| ND2 | *G.ellingtonae-chr1* | *S.stercoralis* | GMYN | 0.55 | 3.11 | 0.18 | 0 |
| ND3 | *H.schachtii* | *Caenorhabditis-sp* | GMYN | 0.60 | 2.21 | 0.27 | 0 |
| ND3 | *H.schachtii* | *G.ellingtonae-chr1* | GMYN | 0.50 | 0.77 | 0.65 | 0.149781 |
| ND3 | *H.schachtii* | *H.cardiolata* | GMYN | 0.49 | 0.91 | 0.53 | 0.0485815 |
| ND3 | *H.schachtii* | *H.glycines* | GMYN | 0.04 | 0.39 | 0.11 | 9.46708E-07 |
| ND3 | *H.schachtii* | *H.trifolii* | GMYN | 0.11 | 0.35 | 0.30 | 0.000311382 |
| ND3 | *H.schachtii* | *K.sudhausi* | GMYN | 0.55 | 2.35 | 0.23 | 0 |
| ND3 | *H.schachtii* | *Litoditis-aff.marina-Pml* | GMYN | 0.60 | 2.07 | 0.29 | 0 |
| ND3 | *H.schachtii* | *O.columbianum* | GMYN | 0.65 | 2.32 | 0.28 | 0.000201315 |
| ND3 | *H.schachtii* | *P.chalcoensis* | GMYN | 0.29 | 2.20 | 0.13 | 2.10831E-06 |
| ND3 | *H.schachtii* | *S.stercoralis* | GMYN | 0.52 | 2.56 | 0.20 | 7.89774E-09 |
| ND3 | *H.schachtii* | *U.sanguinis-haplo45* | GMYN | 0.59 | 2.36 | 0.25 | 0 |
| ND3 | *H.trifolii* | *Caenorhabditis-sp* | GMYN | 0.58 | 2.14 | 0.27 | 0 |
| ND3 | *H.trifolii* | *G.ellingtonae-chr1* | GMYN | 0.48 | 0.91 | 0.53 | 0.142532 |
| ND3 | *H.trifolii* | *H.cardiolata* | GMYN | 0.48 | 1.11 | 0.43 | 0.0620751 |
| ND3 | *H.trifolii* | *H.glycines* | GMYN | 0.13 | 0.26 | 0.51 | 0.0327653 |
| ND3 | *H.trifolii* | *K.sudhausi* | GMYN | 0.51 | 2.22 | 0.23 | 0 |
| ND3 | *H.trifolii* | *Litoditis-aff.marina-Pml* | GMYN | 0.57 | 2.18 | 0.26 | 0 |
| ND3 | *H.trifolii* | *O.columbianum* | GMYN | 0.61 | 2.38 | 0.26 | 1.14253E-08 |
| ND3 | *H.trifolii* | *P.chalcoensis* | GMYN | 0.33 | 1.30 | 0.25 | 0.00130559 |
| ND3 | *H.trifolii* | *S.stercoralis* | GMYN | 0.58 | 1.35 | 0.43 | 0.00339049 |
| ND3 | *H.trifolii* | *U.sanguinis-haplo45* | GMYN | 0.62 | 1.79 | 0.34 | 0.000866989 |
| ND3 | *H.glycines* | *Caenorhabditis-sp* | GMYN | 0.58 | 1.94 | 0.30 | 0 |
| ND3 | *H.glycines* | *G.ellingtonae-chr1* | GMYN | 0.50 | 0.96 | 0.52 | 0.0999577 |
| ND3 | *H.glycines* | *H.cardiolata* | GMYN | 0.46 | 2.46 | 0.19 | 0.030023 |
| ND3 | *H.glycines* | *K.sudhausi* | GMYN | 0.53 | 2.12 | 0.25 | 0 |
| ND3 | *H.glycines* | *Litoditis-aff.marina-Pml* | GMYN | 0.58 | 1.86 | 0.31 | 0 |
| ND3 | *H.glycines* | *O.columbianum* | GMYN | 0.61 | 2.09 | 0.29 | 0 |
| ND3 | *H.glycines* | *S.stercoralis* | GMYN | 0.52 | 2.48 | 0.21 | 6.66676E-07 |
| ND3 | *H.glycines* | *U.sanguinis-haplo45* | GMYN | 0.58 | 2.13 | 0.27 | 0 |
| ND3 | *H.cardiolata* | *Caenorhabditis-sp* | GMYN | 0.60 | 1.99 | 0.30 | 6.00029E-05 |

| | | | | | | | |
|---|---|---|---|---|---|---|---|
| ND3 | *H.cardiolata* | *G.ellingtonae-chr1* | GMYN | 0.50 | 2.70 | 0.18 | 9.15913E-08 |
| ND3 | *H.cardiolata* | *K.sudhausi* | GMYN | 0.60 | 2.08 | 0.29 | 0.000687201 |
| ND3 | *H.cardiolata* | *Litoditis-aff.marina-Pml* | GMYN | 0.58 | 1.89 | 0.30 | 0 |
| ND3 | *H.cardiolata* | *O.columbianum* | GMYN | 0.61 | 0.76 | 0.81 | 0.67213 |
| ND3 | *H.cardiolata* | *P.chalcoensis* | GMYN | 0.39 | 1.87 | 0.21 | 0 |
| ND3 | *H.cardiolata* | *S.stercoralis* | GMYN | 0.52 | 0.50 | 1.03 | 0.710178 |
| ND3 | *H.cardiolata* | *U.sanguinis-haplo45* | GMYN | 0.59 | 2.30 | 0.26 | 0.00034205 |
| ND3 | *P.chalcoensis* | *Caenorhabditis-sp* | GMYN | 0.43 | 2.47 | 0.18 | 1.03923E-11 |
| ND3 | *P.chalcoensis* | *G.ellingtonae-chr1* | GMYN | 0.38 | 0.79 | 0.47 | 0.0311522 |
| ND3 | *P.chalcoensis* | *K.sudhausi* | GMYN | 0.46 | 2.54 | 0.18 | 0 |
| ND3 | *P.chalcoensis* | *Litoditis-aff.marina-Pml* | GMYN | 0.41 | 2.27 | 0.18 | 0 |
| ND3 | *P.chalcoensis* | *O.columbianum* | GMYN | 0.47 | 2.65 | 0.18 | 2.43772E-06 |
| ND3 | *P.chalcoensis* | *S.stercoralis* | GMYN | 0.49 | 2.43 | 0.20 | 1.28921E-05 |
| ND3 | *P.chalcoensis* | *U.sanguinis-haplo45* | GMYN | 0.42 | 2.45 | 0.17 | 0 |
| ND3 | *O.columbianum* | *G.ellingtonae-chr1* | GMYN | 0.57 | 2.03 | 0.28 | 2.10868E-05 |
| ND3 | *O.columbianum* | *K.sudhausi* | GMYN | 0.21 | 0.86 | 0.24 | 3.53408E-05 |
| ND3 | *O.columbianum* | *Litoditis-aff.marina-Pml* | GMYN | 0.20 | 2.59 | 0.08 | 1.16896E-13 |
| ND3 | *O.columbianum* | *S.stercoralis* | GMYN | 0.37 | 2.14 | 0.17 | 8.24256E-07 |
| ND3 | *K.sudhausi* | *Caenorhabditis-sp* | GMYN | 0.23 | 4.18 | 0.06 | 9.31844E-13 |
| ND3 | *K.sudhausi* | *G.ellingtonae-chr1* | GMYN | 0.44 | 2.95 | 0.15 | 7.83014E-12 |
| ND3 | *K.sudhausi* | *Litoditis-aff.marina-Pml* | GMYN | 0.19 | 2.53 | 0.07 | 7.73928E-16 |
| ND3 | *K.sudhausi* | *S.stercoralis* | GMYN | 0.38 | 2.65 | 0.14 | 1.87768E-08 |
| ND3 | *K.sudhausi* | *U.sanguinis-haplo45* | GMYN | 0.27 | 0.74 | 0.36 | 0.00050705 |
| ND3 | *Caenorhabditis-sp* | *G.ellingtonae-chr1* | GMYN | 0.50 | 2.90 | 0.17 | 7.13633E-12 |
| ND3 | *Caenorhabditis-sp* | *Litoditis-aff.marina-Pml* | GMYN | 0.11 | 2.41 | 0.04 | 9.83584E-21 |
| ND3 | *Caenorhabditis-sp* | *U.sanguinis-haplo45* | GMYN | 0.19 | 1.88 | 0.10 | 8.00415E-11 |
| ND3 | *U.sanguinis-haplo45* | *G.ellingtonae-chr1* | GMYN | 0.57 | 1.68 | 0.34 | 7.73402E-05 |
| ND3 | *U.sanguinis-haplo45* | *Litoditis-aff.marina-Pml* | GMYN | 0.17 | 1.99 | 0.08 | 5.802E-13 |
| ND3 | *U.sanguinis-haplo45* | *S.stercoralis* | GMYN | 0.34 | 1.58 | 0.22 | 1.05458E-06 |
| ND3 | *Litoditis-aff.marina-Pml* | *G.ellingtonae-chr1* | GMYN | 0.46 | 2.80 | 0.16 | 3.00611E-10 |
| ND3 | *Litoditis-aff.marina-Pml* | *S.stercoralis* | GMYN | 0.34 | 2.56 | 0.13 | 2.93167E-09 |
| ND3 | *G.ellingtonae-chr1* | *S.stercoralis* | GMYN | 0.55 | 2.70 | 0.20 | 2.07131E-12 |

| | | | | | | | |
|---|---|---|---|---|---|---|---|
| ND4 | *H.schachtii* | *Caenorhabditis-sp* | GMYN | 0.50 | 3.52 | 0.14 | 1.44649E-31 |
| ND4 | *H.schachtii* | *G.ellingtonae-chr1* | GMYN | 0.26 | 3.75 | 0.07 | 1.28292E-61 |
| ND4 | *H.schachtii* | *H.cardiolata* | GMYN | 0.19 | 3.01 | 0.06 | 7.90811E-24 |
| ND4 | *H.schachtii* | *H.glycines* | GMYN | 0.06 | 1.92 | 0.03 | 2.44186E-38 |
| ND4 | *H.schachtii* | *H.trifolii* | GMYN | 0.06 | 1.53 | 0.04 | 3.2531E-50 |
| ND4 | *H.schachtii* | *K.sudhausi* | GMYN | 0.47 | 3.62 | 0.13 | 6.28712E-31 |
| ND4 | *H.schachtii* | *Litoditis-aff.marina-Pml* | GMYN | 0.48 | 3.46 | 0.14 | 8.69503E-42 |
| ND4 | *H.schachtii* | *O.columbianum* | GMYN | 0.53 | 3.55 | 0.15 | 3.39087E-29 |
| ND4 | *H.schachtii* | *P.chalcoensis* | GMYN | 0.25 | 3.51 | 0.07 | 1.66426E-39 |
| ND4 | *H.schachtii* | *S.stercoralis* | GMYN | 0.38 | 3.38 | 0.11 | 0 |
| ND4 | *H.schachtii* | *U.sanguinis-haplo45* | GMYN | 0.50 | 3.56 | 0.14 | 3.28182E-31 |
| ND4 | *H.trifolii* | *Caenorhabditis-sp* | GMYN | 0.50 | 3.37 | 0.15 | 1.66703E-28 |
| ND4 | *H.trifolii* | *G.ellingtonae-chr1* | GMYN | 0.25 | 3.60 | 0.07 | 0 |
| ND4 | *H.trifolii* | *H.cardiolata* | GMYN | 0.18 | 2.81 | 0.06 | 4.24464E-22 |
| ND4 | *H.trifolii* | *H.glycines* | GMYN | 0.09 | 3.45 | 0.03 | 2.11248E-61 |
| ND4 | *H.trifolii* | *K.sudhausi* | GMYN | 0.47 | 3.52 | 0.13 | 1.44683E-31 |
| ND4 | *H.trifolii* | *Litoditis-aff.marina-Pml* | GMYN | 0.46 | 3.21 | 0.14 | 0 |
| ND4 | *H.trifolii* | *O.columbianum* | GMYN | 0.51 | 3.44 | 0.15 | 2.16974E-26 |
| ND4 | *H.trifolii* | *P.chalcoensis* | GMYN | 0.25 | 3.25 | 0.08 | 0 |
| ND4 | *H.trifolii* | *S.stercoralis* | GMYN | 0.36 | 3.26 | 0.11 | 0 |
| ND4 | *H.trifolii* | *U.sanguinis-haplo45* | GMYN | 0.47 | 3.43 | 0.14 | 3.17029E-28 |
| ND4 | *H.glycines* | *Caenorhabditis-sp* | GMYN | 0.50 | 3.37 | 0.15 | 1.62164E-21 |
| ND4 | *H.glycines* | *G.ellingtonae-chr1* | GMYN | 0.29 | 3.66 | 0.08 | 5.05889E-55 |
| ND4 | *H.glycines* | *H.cardiolata* | GMYN | 0.18 | 2.84 | 0.06 | 2.91749E-25 |
| ND4 | *H.glycines* | *K.sudhausi* | GMYN | 0.48 | 3.54 | 0.14 | 3.40991E-24 |
| ND4 | *H.glycines* | *Litoditis-aff.marina-Pml* | GMYN | 0.46 | 3.26 | 0.14 | 0 |
| ND4 | *H.glycines* | *O.columbianum* | GMYN | 0.52 | 3.41 | 0.15 | 6.95167E-34 |
| ND4 | *H.glycines* | *P.chalcoensis* | GMYN | 0.27 | 3.30 | 0.08 | 1.86373E-40 |
| ND4 | *H.glycines* | *S.stercoralis* | GMYN | 0.36 | 3.20 | 0.11 | 0 |
| ND4 | *H.glycines* | *U.sanguinis-haplo45* | GMYN | 0.48 | 3.35 | 0.14 | 2.87411E-38 |
| ND4 | *H.cardiolata* | *Caenorhabditis-sp* | GMYN | 0.50 | 1.61 | 0.31 | 1.38867E-06 |
| ND4 | *H.cardiolata* | *G.ellingtonae-chr1* | GMYN | 0.22 | 3.17 | 0.07 | 3.77664E-30 |
| ND4 | *H.cardiolata* | *K.sudhausi* | GMYN | 0.53 | 0.90 | 0.59 | 0.0433976 |
| ND4 | *H.cardiolata* | *Litoditis-aff.marina-Pml* | GMYN | 0.43 | 1.94 | 0.22 | 1.6937E-06 |
| ND4 | *H.cardiolata* | *O.columbianum* | GMYN | 0.52 | 2.94 | 0.18 | 3.57812E-09 |
| ND4 | *H.cardiolata* | *P.chalcoensis* | GMYN | 0.20 | 2.83 | 0.07 | 4.34648E-20 |
| ND4 | *H.cardiolata* | *S.stercoralis* | GMYN | 0.40 | 2.62 | 0.15 | 1.21416E-15 |

| | | | | | | | |
|---|---|---|---|---|---|---|---|
| ND4 | *H.cardiolata* | *U.sanguinis-haplo45* | GMYN | 0.53 | 0.94 | 0.57 | 0.0303706 |
| ND4 | *P.chalcoensis* | *Caenorhabditis-sp* | GMYN | 0.49 | 3.24 | 0.15 | 0 |
| ND4 | *P.chalcoensis* | *G.ellingtonae-chr1* | GMYN | 0.23 | 3.77 | 0.06 | 1.85978E-55 |
| ND4 | *P.chalcoensis* | *K.sudhausi* | GMYN | 0.54 | 3.47 | 0.16 | 4.54246E-20 |
| ND4 | *P.chalcoensis* | *Litoditis-aff.marina-Pml* | GMYN | 0.51 | 3.13 | 0.16 | 0 |
| ND4 | *P.chalcoensis* | *O.columbianum* | GMYN | 0.52 | 3.31 | 0.16 | 0 |
| ND4 | *P.chalcoensis* | *S.stercoralis* | GMYN | 0.43 | 3.16 | 0.14 | 0 |
| ND4 | *P.chalcoensis* | *U.sanguinis-haplo45* | GMYN | 0.50 | 3.34 | 0.15 | 1.00351E-33 |
| ND4 | *O.columbianum* | *Caenorhabditis-sp* | GMYN | 0.17 | 3.67 | 0.05 | 5.84823E-68 |
| ND4 | *O.columbianum* | *G.ellingtonae-chr1* | GMYN | 0.56 | 3.68 | 0.15 | 0 |
| ND4 | *O.columbianum* | *K.sudhausi* | GMYN | 0.22 | 3.70 | 0.06 | 2.99493E-65 |
| ND4 | *O.columbianum* | *Litoditis-aff.marina-Pml* | GMYN | 0.15 | 3.59 | 0.04 | 1.07514E-63 |
| ND4 | *O.columbianum* | *S.stercoralis* | GMYN | 0.37 | 3.50 | 0.11 | 0 |
| ND4 | *O.columbianum* | *U.sanguinis-haplo45* | GMYN | 0.10 | 1.80 | 0.06 | 2.83165E-61 |
| ND4 | *K.sudhausi* | *Caenorhabditis-sp* | GMYN | 0.16 | 3.83 | 0.04 | 2.04909E-58 |
| ND4 | *K.sudhausi* | *G.ellingtonae-chr1* | GMYN | 0.56 | 3.79 | 0.15 | 8.14627E-31 |
| ND4 | *K.sudhausi* | *Litoditis-aff.marina-Pml* | GMYN | 0.13 | 3.56 | 0.04 | 0 |
| ND4 | *K.sudhausi* | *S.stercoralis* | GMYN | 0.39 | 3.59 | 0.11 | 3.68751E-47 |
| ND4 | *K.sudhausi* | *U.sanguinis-haplo45* | GMYN | 0.23 | 3.76 | 0.06 | 9.03639E-54 |
| ND4 | *Caenorhabditis-sp* | *G.ellingtonae-chr1* | GMYN | 0.56 | 3.62 | 0.16 | 1.08053E-48 |
| ND4 | *Caenorhabditis-sp* | *Litoditis-aff.marina-Pml* | GMYN | 0.08 | 3.48 | 0.02 | 0 |
| ND4 | *Caenorhabditis-sp* | *S.stercoralis* | GMYN | 0.32 | 3.39 | 0.09 | 0 |
| ND4 | *Caenorhabditis-sp* | *U.sanguinis-haplo45* | GMYN | 0.19 | 3.71 | 0.05 | 1.3095E-50 |
| ND4 | *U.sanguinis-haplo45* | *G.ellingtonae-chr1* | GMYN | 0.55 | 3.70 | 0.15 | 5.8363E-44 |
| ND4 | *U.sanguinis-haplo45* | *Litoditis-aff.marina-Pml* | GMYN | 0.16 | 3.56 | 0.04 | 0 |
| ND4 | *U.sanguinis-haplo45* | *S.stercoralis* | GMYN | 0.36 | 3.47 | 0.10 | 0 |
| ND4 | *Litoditis-aff.marina-Pml* | *G.ellingtonae-chr1* | GMYN | 0.57 | 3.68 | 0.15 | 1.08025E-49 |
| ND4 | *Litoditis-aff.marina-Pml* | *S.stercoralis* | GMYN | 0.33 | 3.36 | 0.10 | 0 |
| ND4 | *G.ellingtonae-chr1* | *S.stercoralis* | GMYN | 0.45 | 3.38 | 0.13 | 0 |
| ND4L | *H.trifolii* | *Caenorhabditis-sp* | GMYN | 0.64 | 1.73 | 0.37 | 0 |
| ND4L | *H.trifolii* | *G.ellingtonae-chr1* | GMYN | 0.41 | 2.36 | 0.17 | 1.91589E-06 |
| ND4L | *H.trifolii* | *H.glycines* | GMYN | 0.67 | 0.44 | 1.50 | 0.336278 |

| | | | | | | | |
|---|---|---|---|---|---|---|---|
| ND4L | *H.trifolii* | *K.sudhausi* | GMYN | 0.71 | 1.98 | 0.36 | 0.0105059 |
| ND4L | *H.trifolii* | *Litoditis-aff.marina-Pml* | GMYN | 0.63 | 1.68 | 0.38 | 0 |
| ND4L | *H.trifolii* | *O.columbianum* | GMYN | 0.68 | 2.14 | 0.32 | 0 |
| ND4L | *H.trifolii* | *S.stercoralis* | GMYN | 0.59 | 1.75 | 0.34 | 0 |
| ND4L | *H.trifolii* | *U.sanguinis-haplo45* | GMYN | 0.74 | 1.98 | 0.37 | 0 |
| ND4L | *H.glycines* | *Caenorhabditis-sp* | GMYN | 0.91 | 1.16 | 0.79 | 0.999996 |
| ND4L | *H.glycines* | *G.ellingtonae-chr1* | GMYN | 1.91 | 0.33 | 5.75 | 1.0815E-06 |
| ND4L | *H.glycines* | *K.sudhausi* | GMYN | 1.32 | 0.62 | 2.14 | 0.00819365 |
| ND4L | *H.glycines* | *Litoditis-aff.marina-Pml* | GMYN | 0.79 | 1.71 | 0.46 | 0 |
| ND4L | *H.glycines* | *O.columbianum* | GMYN | 0.88 | 0.57 | 1.54 | 0.38611 |
| ND4L | *H.glycines* | *S.stercoralis* | GMYN | 0.95 | 0.53 | 1.78 | 0.284317 |
| ND4L | *H.glycines* | *U.sanguinis-haplo45* | GMYN | 1.63 | 1.33 | 1.23 | 0.42875 |
| ND4L | *O.columbianum* | *Caenorhabditis-sp* | GMYN | 0.15 | 1.14 | 0.13 | 1.31719E-08 |
| ND4L | *O.columbianum* | *G.ellingtonae-chr1* | GMYN | 0.63 | 2.02 | 0.31 | 0.000624844 |
| ND4L | *O.columbianum* | *K.sudhausi* | GMYN | 0.29 | 2.64 | 0.11 | 2.51335E-11 |
| ND4L | *O.columbianum* | *Litoditis-aff.marina-Pml* | GMYN | 0.11 | 2.04 | 0.06 | 8.55278E-10 |
| ND4L | *O.columbianum* | *S.stercoralis* | GMYN | 0.41 | 2.17 | 0.19 | 9.57415E-06 |
| ND4L | *O.columbianum* | *U.sanguinis-haplo45* | GMYN | 0.06 | 1.35 | 0.05 | 1.443E-15 |
| ND4L | *K.sudhausi* | *Caenorhabditis-sp* | GMYN | 0.27 | 1.92 | 0.14 | 4.01283E-06 |
| ND4L | *K.sudhausi* | *G.ellingtonae-chr1* | GMYN | 0.72 | 1.70 | 0.42 | 0.0428971 |
| ND4L | *K.sudhausi* | *Litoditis-aff.marina-Pml* | GMYN | 0.20 | 2.41 | 0.08 | 1.82261E-11 |
| ND4L | *K.sudhausi* | *S.stercoralis* | GMYN | 0.49 | 2.24 | 0.22 | 0 |
| ND4L | *Caenorhabditis-sp* | *G.ellingtonae-chr1* | GMYN | 0.60 | 0.95 | 0.63 | 0.358797 |
| ND4L | *Caenorhabditis-sp* | *Litoditis-aff.marina-Pml* | GMYN | 0.09 | 2.16 | 0.04 | 1.72972E-12 |
| ND4L | *Caenorhabditis-sp* | *S.stercoralis* | GMYN | 0.42 | 1.92 | 0.22 | 1.06453E-05 |
| ND4L | *Caenorhabditis-sp* | *U.sanguinis-haplo45* | GMYN | 0.15 | 1.60 | 0.09 | 7.56013E-07 |
| ND4L | *U.sanguinis-haplo45* | *G.ellingtonae-chr1* | GMYN | 0.70 | 1.13 | 0.62 | 0.210899 |
| ND4L | *U.sanguinis-haplo45* | *Litoditis-aff.marina-Pml* | GMYN | 0.16 | 1.71 | 0.09 | 2.13556E-08 |
| ND4L | *U.sanguinis-haplo45* | *S.stercoralis* | GMYN | 0.41 | 2.07 | 0.20 | 0 |
| ND4L | *Litoditis-aff.marina-Pml* | *G.ellingtonae-chr1* | GMYN | 0.69 | 0.70 | 0.98 | 1 |
| ND4L | *G.ellingtonae-chr1* | *S.stercoralis* | GMYN | 0.54 | 2.32 | 0.23 | 0.000207292 |
| ND5 | *H.schachtii* | *Caenorhabditis-sp* | GMYN | 0.51 | 3.41 | 0.15 | 0 |

| | | | | | | | | |
|---|---|---|---|---|---|---|---|---|
| ND5 | *H.schachtii* | *G.ellingtonae-chr1* | GMYN | 0.29 | 3.81 | 0.08 | 0 |
| ND5 | *H.schachtii* | *H.glycines* | GMYN | 0.08 | 1.47 | 0.05 | 1.25535E-48 |
| ND5 | *H.schachtii* | *H.trifolii* | GMYN | 0.06 | 2.05 | 0.03 | 2.34908E-73 |
| ND5 | *H.schachtii* | *K.sudhausi* | GMYN | 0.51 | 3.39 | 0.15 | 0 |
| ND5 | *H.schachtii* | *Litoditis-aff.marina-Pml* | GMYN | 0.50 | 3.16 | 0.16 | 0 |
| ND5 | *H.schachtii* | *O.columbianum* | GMYN | 0.52 | 3.35 | 0.15 | 0 |
| ND5 | *H.schachtii* | *S.stercoralis* | GMYN | 0.36 | 3.11 | 0.11 | 0 |
| ND5 | *H.schachtii* | *U.sanguinis-haplo45* | GMYN | 0.49 | 3.34 | 0.15 | 0 |
| ND5 | *H.trifolii* | *Caenorhabditis-sp* | GMYN | 0.50 | 3.33 | 0.15 | 0 |
| ND5 | *H.trifolii* | *G.ellingtonae-chr1* | GMYN | 0.29 | 3.75 | 0.08 | 0 |
| ND5 | *H.trifolii* | *H.glycines* | GMYN | 0.09 | 3.48 | 0.03 | 3.83543E-54 |
| ND5 | *H.trifolii* | *K.sudhausi* | GMYN | 0.48 | 3.35 | 0.14 | 0 |
| ND5 | *H.trifolii* | *Litoditis-aff.marina-Pml* | GMYN | 0.48 | 3.08 | 0.15 | 0 |
| ND5 | *H.trifolii* | *O.columbianum* | GMYN | 0.50 | 3.27 | 0.15 | 0 |
| ND5 | *H.trifolii* | *S.stercoralis* | GMYN | 0.37 | 3.04 | 0.12 | 0 |
| ND5 | *H.trifolii* | *U.sanguinis-haplo45* | GMYN | 0.46 | 3.26 | 0.14 | 0 |
| ND5 | *H.glycines* | *Caenorhabditis-sp* | GMYN | 0.50 | 3.22 | 0.16 | 0 |
| ND5 | *H.glycines* | *G.ellingtonae-chr1* | GMYN | 0.28 | 3.73 | 0.08 | 0 |
| ND5 | *H.glycines* | *K.sudhausi* | GMYN | 0.52 | 3.28 | 0.16 | 0 |
| ND5 | *H.glycines* | *Litoditis-aff.marina-Pml* | GMYN | 0.53 | 2.98 | 0.18 | 0 |
| ND5 | *H.glycines* | *O.columbianum* | GMYN | 0.52 | 3.36 | 0.16 | 0 |
| ND5 | *H.glycines* | *S.stercoralis* | GMYN | 0.38 | 2.87 | 0.13 | 0 |
| ND5 | *H.glycines* | *U.sanguinis-haplo45* | GMYN | 0.48 | 3.24 | 0.15 | 0 |
| ND5 | *O.columbianum* | *Caenorhabditis-sp* | GMYN | 0.21 | 3.92 | 0.05 | 0 |
| ND5 | *O.columbianum* | *G.ellingtonae-chr1* | GMYN | 0.60 | 3.91 | 0.15 | 1.16875E-42 |
| ND5 | *O.columbianum* | *K.sudhausi* | GMYN | 0.24 | 3.98 | 0.06 | 4.23879E-84 |
| ND5 | *O.columbianum* | *Litoditis-aff.marina-Pml* | GMYN | 0.17 | 3.78 | 0.04 | 0 |
| ND5 | *O.columbianum* | *S.stercoralis* | GMYN | 0.36 | 3.55 | 0.10 | 0 |
| ND5 | *O.columbianum* | *U.sanguinis-haplo45* | GMYN | 0.11 | 1.61 | 0.07 | 7.9705E-63 |
| ND5 | *K.sudhausi* | *Caenorhabditis-sp* | GMYN | 0.21 | 3.84 | 0.05 | 0 |
| ND5 | *K.sudhausi* | *G.ellingtonae-chr1* | GMYN | 0.58 | 3.90 | 0.15 | 1.32925E-59 |
| ND5 | *K.sudhausi* | *Litoditis-aff.marina-Pml* | GMYN | 0.18 | 3.77 | 0.05 | 1.98946E-95 |
| ND5 | *K.sudhausi* | *S.stercoralis* | GMYN | 0.32 | 3.55 | 0.09 | 0 |
| ND5 | *K.sudhausi* | *U.sanguinis-haplo45* | GMYN | 0.24 | 3.93 | 0.06 | 3.45759E-75 |
| ND5 | *Caenorhabditis-sp* | *G.ellingtonae-chr1* | GMYN | 0.58 | 3.78 | 0.15 | 0 |

| | | | | | | | |
|---|---|---|---|---|---|---|---|
| ND5 | *Caenorhabditis-sp* | *Litoditis-aff.marina-Pml* | GMYN | 0.11 | 3.72 | 0.03 | 0 |
| ND5 | *Caenorhabditis-sp* | *S.stercoralis* | GMYN | 0.30 | 3.46 | 0.09 | 0 |
| ND5 | *Caenorhabditis-sp* | *U.sanguinis-haplo45* | GMYN | 0.22 | 1.22 | 0.18 | 5.69091E-41 |
| ND5 | *U.sanguinis-haplo45* | *G.ellingtonae-chr1* | GMYN | 0.52 | 3.81 | 0.14 | 0 |
| ND5 | *U.sanguinis-haplo45* | *Litoditis-aff.marina-Pml* | GMYN | 0.16 | 3.78 | 0.04 | 0 |
| ND5 | *U.sanguinis-haplo45* | *S.stercoralis* | GMYN | 0.31 | 3.50 | 0.09 | 0 |
| ND5 | *Litoditis-aff.marina-Pml* | *G.ellingtonae-chr1* | GMYN | 0.55 | 3.69 | 0.15 | 0 |
| ND5 | *Litoditis-aff.marina-Pml* | *S.stercoralis* | GMYN | 0.31 | 3.41 | 0.09 | 0 |
| ND5 | *G.ellingtonae-chr1* | *S.stercoralis* | GMYN | 0.42 | 3.60 | 0.12 | 0 |
| ND6 | *H.schachtii* | *Caenorhabditis-sp* | GMYN | 0.64 | 2.38 | 0.27 | 0 |
| ND6 | *H.schachtii* | *G.ellingtonae-chr1* | GMYN | 0.45 | 2.33 | 0.19 | 3.26393E-07 |
| ND6 | *H.schachtii* | *H.glycines* | GMYN | 0.09 | 2.55 | 0.03 | 7.72903E-22 |
| ND6 | *H.schachtii* | *H.trifolii* | GMYN | 0.09 | 2.60 | 0.03 | 5.39764E-21 |
| ND6 | *H.schachtii* | *K.sudhausi* | GMYN | 0.73 | 2.58 | 0.28 | 0 |
| ND6 | *H.schachtii* | *Litoditis-aff.marina-Pml* | GMYN | 0.61 | 2.40 | 0.26 | 0.000479941 |
| ND6 | *H.schachtii* | *O.columbianum* | GMYN | 0.65 | 2.38 | 0.27 | 0 |
| ND6 | *H.schachtii* | *S.stercoralis* | GMYN | 0.51 | 1.19 | 0.43 | 0.0375921 |
| ND6 | *H.schachtii* | *U.sanguinis-haplo45* | GMYN | 0.70 | 1.31 | 0.53 | 0.081906 |
| ND6 | *H.trifolii* | *Caenorhabditis-sp* | GMYN | 0.50 | 2.30 | 0.22 | 0 |
| ND6 | *H.trifolii* | *G.ellingtonae-chr1* | GMYN | 0.52 | 1.32 | 0.39 | 0.00108198 |
| ND6 | *H.trifolii* | *H.glycines* | GMYN | 0.09 | 2.68 | 0.03 | 1.73707E-21 |
| ND6 | *H.trifolii* | *K.sudhausi* | GMYN | 0.64 | 2.48 | 0.26 | 0 |
| ND6 | *H.trifolii* | *Litoditis-aff.marina-Pml* | GMYN | 0.66 | 2.53 | 0.26 | 6.38994E-05 |
| ND6 | *H.trifolii* | *O.columbianum* | GMYN | 0.50 | 2.01 | 0.25 | 0 |
| ND6 | *H.trifolii* | *S.stercoralis* | GMYN | 0.42 | 2.09 | 0.20 | 0 |
| ND6 | *H.trifolii* | *U.sanguinis-haplo45* | GMYN | 0.58 | 2.15 | 0.27 | 0 |
| ND6 | *H.glycines* | *Caenorhabditis-sp* | GMYN | 0.52 | 2.25 | 0.23 | 0 |
| ND6 | *H.glycines* | *G.ellingtonae-chr1* | GMYN | 0.48 | 2.24 | 0.21 | 3.22101E-07 |
| ND6 | *H.glycines* | *K.sudhausi* | GMYN | 0.63 | 2.61 | 0.24 | 0 |
| ND6 | *H.glycines* | *Litoditis-aff.marina-Pml* | GMYN | 0.65 | 2.47 | 0.26 | 0.000426001 |
| ND6 | *H.glycines* | *O.columbianum* | GMYN | 0.55 | 2.12 | 0.26 | 0 |
| ND6 | *H.glycines* | *S.stercoralis* | GMYN | 0.50 | 1.48 | 0.33 | 0.0266766 |
| ND6 | *H.glycines* | *U.sanguinis-haplo45* | GMYN | 0.63 | 2.30 | 0.27 | 0 |

| | | | | | | | |
|---|---|---|---|---|---|---|---|
| ND6 | *O.columbianum* | *Caenorhabditis-sp* | GMYN | 0.28 | 2.50 | 0.11 | 3.3966E-12 |
| ND6 | *O.columbianum* | *G.ellingtonae-chr1* | GMYN | 0.56 | 2.81 | 0.20 | 3.18751E-12 |
| ND6 | *O.columbianum* | *K.sudhausi* | GMYN | 0.36 | 2.72 | 0.13 | 0 |
| ND6 | *O.columbianum* | *Litoditis-aff.marina-Pml* | GMYN | 0.26 | 2.91 | 0.09 | 6.3735E-16 |
| ND6 | *O.columbianum* | *S.stercoralis* | GMYN | 0.40 | 2.41 | 0.17 | 0 |
| ND6 | *O.columbianum* | *U.sanguinis-haplo45* | GMYN | 0.18 | 0.73 | 0.24 | 1.58125E-08 |
| ND6 | *K.sudhausi* | *Caenorhabditis-sp* | GMYN | 0.37 | 2.58 | 0.14 | 0 |
| ND6 | *K.sudhausi* | *G.ellingtonae-chr1* | GMYN | 0.69 | 3.08 | 0.22 | 3.45818E-10 |
| ND6 | *K.sudhausi* | *Litoditis-aff.marina-Pml* | GMYN | 0.31 | 2.53 | 0.12 | 0 |
| ND6 | *K.sudhausi* | *S.stercoralis* | GMYN | 0.40 | 2.53 | 0.16 | 0 |
| ND6 | *K.sudhausi* | *U.sanguinis-haplo45* | GMYN | 0.37 | 2.78 | 0.13 | 0 |
| ND6 | *Caenorhabditis-sp* | *G.ellingtonae-chr1* | GMYN | 0.63 | 2.98 | 0.21 | 1.10785E-08 |
| ND6 | *Caenorhabditis-sp* | *Litoditis-aff.marina-Pml* | GMYN | 0.21 | 2.65 | 0.08 | 0 |
| ND6 | *Caenorhabditis-sp* | *S.stercoralis* | GMYN | 0.36 | 2.45 | 0.15 | 0 |
| ND6 | *Caenorhabditis-sp* | *U.sanguinis-haplo45* | GMYN | 0.31 | 2.81 | 0.11 | 8.62143E-15 |
| ND6 | *U.sanguinis-haplo45* | *G.ellingtonae-chr1* | GMYN | 0.66 | 2.71 | 0.24 | 0.00203342 |
| ND6 | *U.sanguinis-haplo45* | *Litoditis-aff.marina-Pml* | GMYN | 0.27 | 2.78 | 0.10 | 1.23825E-18 |
| ND6 | *U.sanguinis-haplo45* | *S.stercoralis* | GMYN | 0.48 | 2.40 | 0.20 | 0 |
| ND6 | *Litoditis-aff.marina-Pml* | *G.ellingtonae-chr1* | GMYN | 0.75 | 3.06 | 0.24 | 8.39312E-07 |
| ND6 | *Litoditis-aff.marina-Pml* | *S.stercoralis* | GMYN | 0.42 | 2.42 | 0.17 | 0 |
| ND6 | *G.ellingtonae-chr1* | *S.stercoralis* | GMYN | 0.42 | 2.96 | 0.14 | 1.53048E-10 |

## Supplementary figures

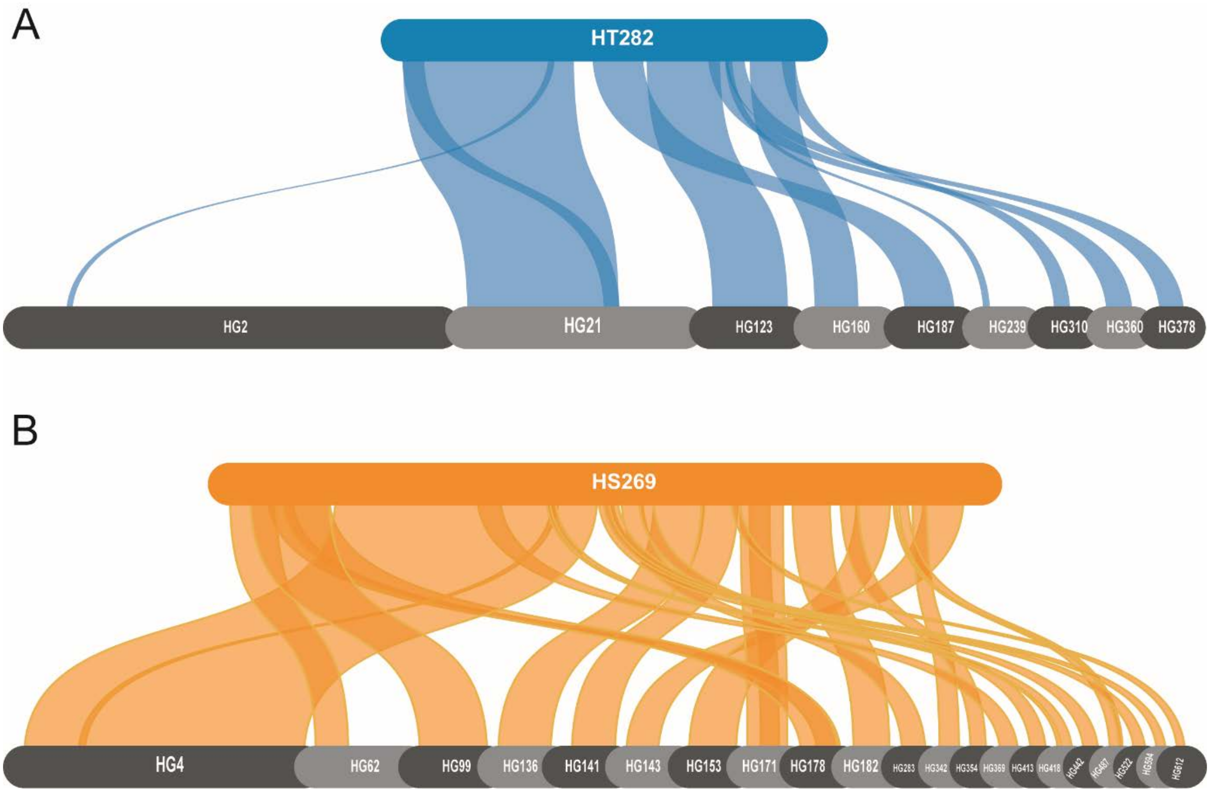


**Figure S1.** Largest collinear blocks in nematodes HT (A) and HS (B) and their corresponding syntenic blocks in genome of nematode HG

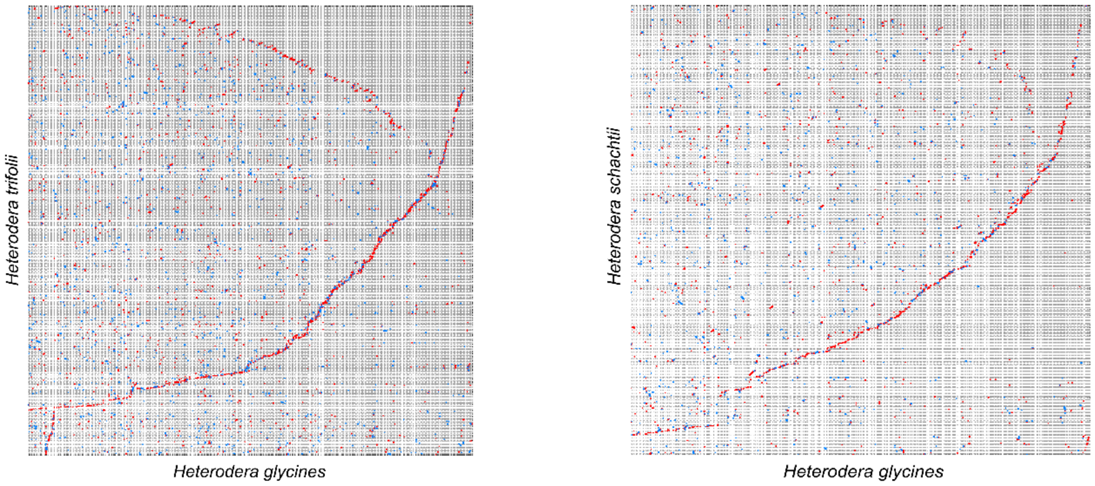


**A** **B**

**Figure S2.** Dot plot of syntenic genes in HT (A) and HS (B) plotted against HG as a reference genome confirming global duplication pattern in HT genome

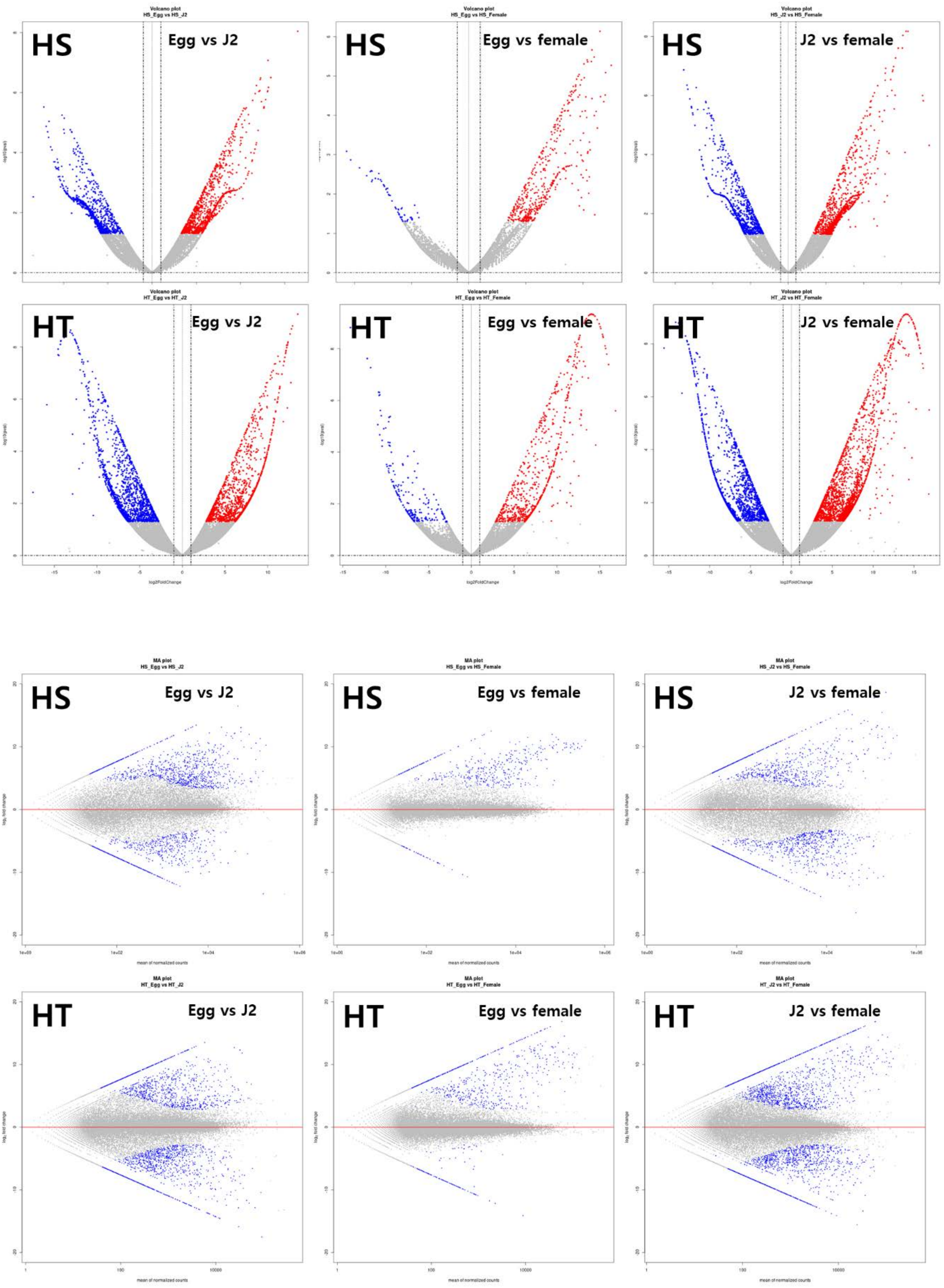


**Figure S3.** Volcano charts of Differentially expressed genes (DEG) across various life stages of HS and HT

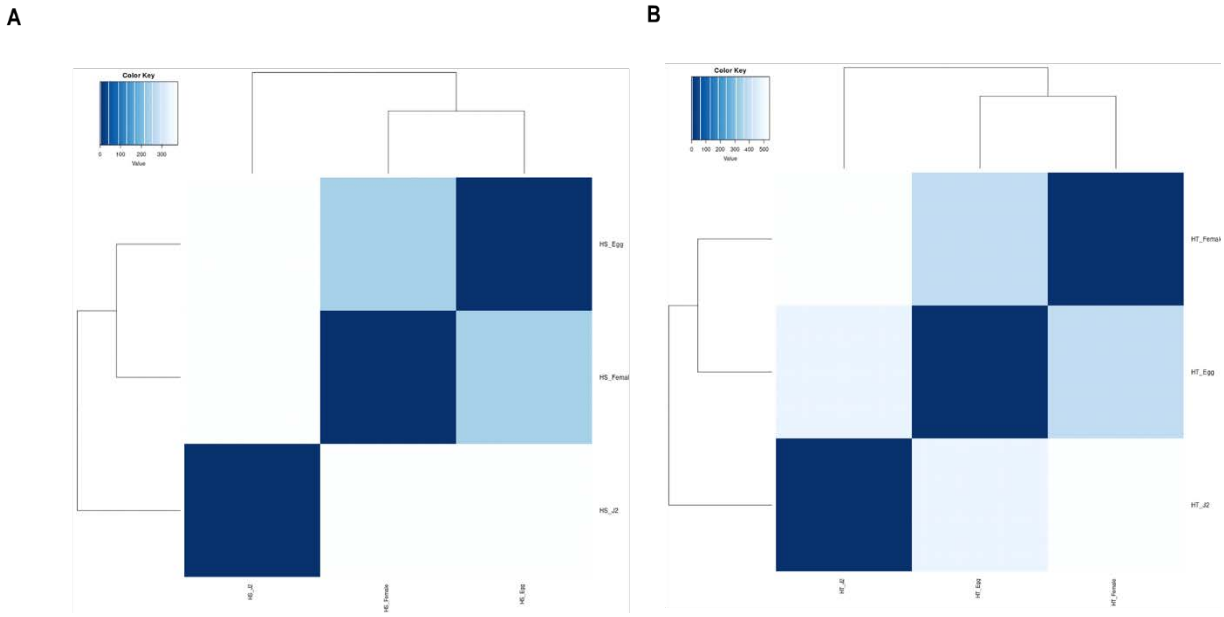


**Figure S4.** Correlation analysis of Differentially expressed genes (DEG) across various life stages of HS and HT

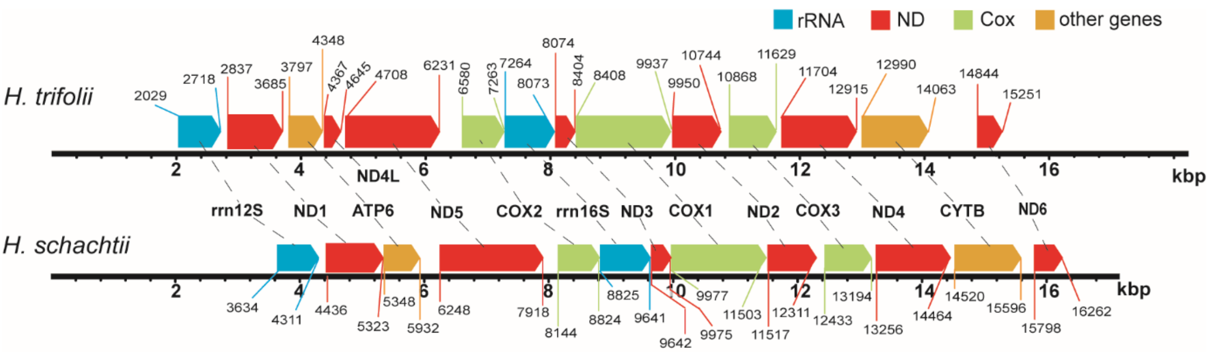


**Figure S5.** Structure and order of protein coding genes (PCGs) present in mitochondrial genomes of cyst nematodes HT and HS
